# Metformin induces pyroptosis in leptin receptor-defective hepatocytes via overactivation of the AMPK axis

**DOI:** 10.1038/s41419-023-05623-4

**Published:** 2023-02-03

**Authors:** Bingli Liu, Jingyuan Xu, Linyao Lu, Lili Gao, Shengjuan Zhu, Yi Sui, Ting Cao, Tao Yang

**Affiliations:** 1 Department of Orthopedics, Shanghai Pudong New Area People’s Hospital, Shanghai, 201299 China; 2 Department of Gastroenterology, Shanghai Pudong Hospital, Fudan University Pudong Medical Center, Shanghai, 201399 China; 3 Center for Medical Research and Innovation, Shanghai Pudong Hospital, Fudan University Pudong Medical Center, Shanghai, 201399 China; 4 Department of Nutrition, The First Affiliated Hospital of Sun Yat-sen University, Guangzhou, 510080 China

**Keywords:** Non-alcoholic fatty liver disease, Cell death

## Abstract

Metformin is the biguanide of hepatic insulin sensitizer for patients with non-alcohol fatty liver disease (NAFLD). Findings regarding its efficacy in restoring blood lipids and liver histology have been contradictory. In this study, we explore metformin’s preventive effects on NAFLD in leptin-insensitive individuals. We used liver tissue, serum exosomes and isolated hepatocytes from high-fat diet (HFD)-induced Zucker diabetic fatty (ZDF) rats and leptin receptor (Lepr) knockout rats to investigate the correlation between hepatic Lepr defective and liver damage caused by metformin. Through immunostaining, RT-PCR and glucose uptake monitoring, we showed that metformin treatment activates adenosine monophosphate (AMP)-activated protein kinase (AMPK) and its downstream cytochrome C oxidase (CCO). This leads to overactivation of glucose catabolism-related genes, excessive energy repertoire consumption, and subsequent hepatocyte pyroptosis. Single-cell RNA sequencing further confirmed the hyper-activation of glucose catabolism after metformin treatment. Altogether, we showed that functional Lepr is necessary for metformin treatment to be effective, and that long-term metformin treatment might promote NAFLD progression in leptin-insensitive individuals. This provides important insight into the clinical application of metformin.

## Background

Non-alcoholic fatty liver disease (NAFLD) is a primary consequence of insulin resistance. It can progress into non-alcoholic steatohepatitis (NASH), fibrosis, cirrhosis or hepatocellular carcinoma. This is becoming an increasingly prevalent global health problem [1]. Although it can be treated through diet and lifestyle intervention, the long-term success rate is low [2]. In addition to liver damage, NAFLD also increases the risk of other metabolic syndromes, including type 2 diabetes (T2D) [3].

Metformin, a biguanide that improves hepatic insulin resistance and hyperglycemia, is the first-line therapeutic agent for T2D patients, especially those with concomitant hyperlipidemia and obesity [4, 5]. In recent years, studies have examined the molecular mechanism of metformin in inhibiting hepatic gluconeogenesis and lowering blood glucose levels [6]. Some have suggested that metformin could stimulate adenosine monophosphate (AMP)-activated protein kinase (AMPK), an enzyme that responds to the natural depletion of cellular energy [7]. AMPK has been implicated in both glucose uptake stimulation in skeletal muscles and the inhibition of hepatic gluconeogenesis [7]. Therefore, metformin might regulate these metabolic pathways by interacting with AMPK, mediating its beneficial effect in lowing blood glucose. In addition, metformin has also been evaluated as a candidate therapeutic drug for NAFLD treatment, as some patients have shown short-term improvement in liver function after treatment [8–11]. It has been suggested that short-term metformin administration could benefit patients by lowering their blood lipid levels, thus protecting hepatocytes from lipid accumulation [5, 12, 13]. On the other hand, studies have also associated metformin with hepatotoxicity including acute hepatitis and cholestasis [14–18] in T2D patients. In all cases, hepatic enzyme concentration was elevated in patients after several weeks of metformin administration, suggesting inflammation or other liver cell damage [14–18]. These adverse events can be the impetus behind the discontinuation of metformin treatment. Furthermore, the mechanism underlying metformin-induced hepatotoxicity also remains uncertain.

Leptin is a polypeptide hormone produced primarily by adipocytes. It provides catabolic signals to the central nervous system (CNS) when body fat increases and/or nutrition is excessive [19–22]. Numerous studies have shown the importance of leptin in the pathogenesis and progression of hepatic steatosis [23–25]. By interacting with the leptin receptor (Lepr), a member of the class I cytokine receptors, leptin reduces appetite and promotes energy expenditure [26–28]. Lepr defect is the purest form of leptin resistance, and it directly causes obesity and insulin resistance [29]. Like leptin deficiency, in humans and rodents, Lepr b deficiency shows a significant decrease in metabolic rates, and an increase in body fat and insulin resistance [29]. Lepr is expressed in both CNS and peripheral tissues (for instance, the liver). Upon leptin stimulation, Lepr in the liver increases in expression level, and subsequently produces soluble Lepr, which can inhibit the amount of circulating leptin [30]. The mechanism underlying metformin-induced hepatotoxicity also remains unclear, and we speculate that its adverse effects might be associated with leptin deficiency or malfunctioning Lepr. In leptin-deficient mice, insufficient activation of AMPK, the molecular target of metformin, promotes insulin resistance and obesity [20, 21]. However, Lepr deficiency might also have health consequences [31, 32]. Studies have shown that Lepr functionality is critical to diabetes control, and may be a factor in liver complications, as leptin resistance has been associated with the pathogenesis and progression of T2D, hepatic steatosis and fibrosis [22, 23]. Therefore, metformin may have different effects among NAFLD patients preconditioned with different genetic defectives in Lepr. Thus, more precautions are needed when the drug is prescribed. It has also been suggested that leptin and its receptor can promote inflammation and fibrosis in the liver [31, 32]. Our study showed that in Zucker diabetic fatty (ZDF) rats which are characterized by homozygous mutation in *Lepr* gene, Lepr defective can offset metformin’s therapeutic effect on liver steatosis and weight gain, and instead lead to liver function damage. To assess the potential for a relationship between Lepr signaling pathway and liver damage caused by metformin administration, we selected Lepr as the hypothetical acting gene, and conducted an association analysis based on Lepr defective, metformin administration and liver function. Then, we analyzed the relationship between influence of Lepr deficiency on downstream signaling pathway and liver pathological phenotype. Owing to an inherent mutation of the Lepr, adult ZDF rats develop hyperlipidemia, hyperglycemia, hyperinsulinemia, insulin-resistance, fatty liver and hepatocyte degeneration under the influence of a high-fat diet (HFD) [33–35]. In this study, we used HFD-fed ZDF rats as diabetic rat models, and compared them with their healthy counterparts (Zucker lean rats) [34, 35]. Additionally, we explored leptin resistance using a homogenous Lepr-deficient (lepr^-/-^) rat model. To investigate the long-term effects of metformin treatment on the development of hyperlipidemia and fatty liver disease, we treated young ZDF rats with metformin for 6 months. Then, we extracted exosomes containing lipids, RNA, and protein from rat blood, and co-cultured them with rat liver cell line BRL 3 A. To assess the potential adverse effects of Lepr defective during metformin administration, we utilized isolated hepatocytes. The aim of the present study is to elucidate the potential effect of hepatic Lepr expression on metformin treatment for NAFLD. This could inform treatment selection, and help screen out inappropriate NAFLD populations which are not ideal candidates for metformin treatment.

## Methods

### Study animals

Male rats [ZDF, Zucker lean, and Sprague Dawley (SD) rats] at 8 weeks of age were caged in pairs at the Laboratory Animal Services Center at room temperature (23 ± 1 °C) with a 12/12 h dark/light cycle. All animals received water *ad libitum*. The use of animals in this study was approved by the Fudan University Animal Experimentation Ethics Committee in accordance with the Animals (Control of Experiments) Ordinance of China.

### Preparing Lepr knockout (Lepr^−/−^) rats

The *Lepr* knockout (*Lepr*^−/−^) rats were established via the clustered regularly interspaced short palindromic repeats (CRISPR)/CRISPR-associated 9 (Cas9) system. Two pairs of gRNAs (TAG GCA AAT CAT CTA TAA CTT C & AAA CGA AGT TAT AGA TGA TTT G; TAG GCT GAA AGC TGT CTT TCA G & AAA CCT GAA AGA CAG CTT TCA G) targeting Lepr exon 4 were transcribed in vitro. A mixture of Cas9 mRNA (20 ng/μl) and gRNA (10 ng/μl) was microinjected into a zygote obtained from Sprague Dawley (SD) rats, producing nine pups. The rats’ genotype was determined by PCR, the *Lepr* knockout was validated by TA clone and sequencing analysis, and the Lepr protein expression was verified by Western blotting (see below). We selected *Lepr*^-/-^ pups as the founder generation to establish a colony, and used 2-month-old offspring rats (*Lepr*^−/−^) in the subsequent experiments, annotated as Lepr-KO rats.

### Experimental groups

We divided the rats randomly into four groups (ZDF, Zucker lean, SD/Lepr WT, Lepr-KO). For each breed, the normal chow diet (NCD) rats were fed a standard laboratory rat diet (5001 Rodent Diet, LabDiet, St Louis, MO, USA), while the high-fat diet (HFD) rats were fed a fat-rich diet containing 84.5% standard diet chow, 10% yolk powder, 5% lard oil and 0.5% porcine bile salt to induce NAFLD [36]. Twelve rats were included in each subgroup.

### Metformin administration

The metformin dosages were based on a previous study [36]. Each subgroup of rats (*n* = 12) was treated with metformin (met) at 50 mg per kg body weight with distilled water as the vehicle (10 ml per kg), or vehicle only (10 ml per kg), for 6 months. All treatments were orally administered once a day. After treatment for 6 months, we monitored body weight, and performed oral glucose tolerance tests (OGTT) by means of dextrose gavage (2.5 g/kg body weight; Sigma, St. Louis, MO, US) following 8 h (h) of fasting. The rats were sacrificed in a fasting state. Fasting blood samples were taken to measure blood lipids and liver function. 200 g liver tissue from the right lobe of the liver was harvested for western blotting analysis. Freshly collected samples were also subjected to hepatocyte dissociation procedures or fixed in 10% neutral formaldehyde for histological analysis.

### Hepatocyte dissociation

Animals were anesthetized by 3% halothane inhalation, and were maintained on 1.5% halothane in 70% nitrous oxide and 30% oxygen. Preheated perfusate containing 142 mM NaCl, 6.7 mM KCl, 10 mM HEPES and 5.5 mM NaOH was infused into each liver via the portal vein at a velocity of 6 ml/min for 15 min (on the condition that the postcava was cut), followed by perfusion with 0.05% collagenase IV (Sigma) at the velocity of 6 ml/min for 15 min. After harvesting the livers, we cut the Glisson’s Capsule, and washed the discrete hepatocytes with solution containing 142 mM NaCl, 6.7 mM KCl, 1.2 mM CaCl_2_, 10 mM HEPES and 5.5 mM NaOH, followed by cell collection via a 40 μm cell strainer (cat# 352340, BD Biosciences, CA, USA). Hepatocytes isolated from the livers of ZDF rats, Zucker lean rats, Lepr-KO rats, and controls were seeded onto 6-well plates at 2 × 10^5^/well, respectively. Cells were cultured in the medium either with or without metformin (10 μM), or AMPK inhibitor Dorsomorphin (0.5 μm; cat# 171260; Sigma) for 96 h, before they were harvested and subjected to Western blotting analysis. Meanwhile, in a subset of the experiment, cells were pre-treated with cytochrome C oxidase (CCO) inhibitor Daunorubicin (1 μM; cat# 251800; Millipore, Billerica, MA, US) for 24 h, before switching to medium supplemented with metformin (10 μM) and leptin (10 μM; Sigma) for 72 h. After that, cells were harvested and subjected to Western blotting analysis.

### Cell culture

The rat liver cell line BRL 3 A (CRL-1442, American Type Culture Collection (ATCC), Manassas, VA, USA) was cultured in Eagle’s Minimum Essential Medium (EMEM; cat# 30-2003, ATCC) supplemented with 7% fetal bovine serum (FBS; Invitrogen). Cell was authenticated and tested for mycoplasma contamination.

### Histopathological examination and immunocytochemical staining

Liver tissues fixed in neutral formaldehyde were embedded in paraffin. Sections (4 μm) were subject to hematoxylin & eosin (Sigma) staining for routine structural examination according to the manufacturer’s instructions. For immunocytochemical staining of Lepr, liver tissue sections or dissociated hepatocytes seeded onto the coverslips were stained with anti-Lepr (1:100; Rabbit mAb, cat# PIMA532685, Thermo Fisher Scientific, Waltham, MA, US) overnight. Then the biotinylated goat anti-rabbit IgG (1:100; Vector, Burlingame, CA, USA) was applied to sections or cells. After rinsing them, streptavidin-horse radish peroxidase (Vector) was added, and immunoreactivity was visualized by using diaminobenzidine (Vector), followed by counterstaining with hematoxylin. For immunofluorescence study, dissociated hepatocytes at 1 × 10^5^ were cyto-spun onto each cytosmear, which was subject to incubation with the following primary antibodies: Alexa Fluor 488-conjugated mouse anti-CCO (1:100; cat# ab198593, Abcam, Cambridge, MA, US), rabbit anti-phosphorylated AMPK (p-AMPK, 1:100; cat# 07-681, Millipore), goat anti-glucose-6-phosphate dehydrogenase X-linked (G6PDX, 1:100; cat# ab106810, Abcam). Rinsed cells were sequentially stained with Cy^TM^5-conjugated goat anti-rabbit IgG (1:100; cat# 45-001-212, Thermo Fisher Scientific) and Cy3-conjugate donkey anti-goat IgG (1:100; cat# AP180C, Millipore), with a thorough wash before each staining batch, followed by counterstaining with 4’,6-diamidino-2-phenylindole (DAPI; Sigma). Stained sections or cells were examined with a Zeiss Axioplan 2 imaging microscope (Carl Zeiss, Hamburg, Germany), and representative images were captured using a SPOT digital camera (Diagnostic Instruments Inc, Sterling Heights, MI, USA). The hepatocytes’ diameters were detected with Image J (developed by the National Institutes of Health, Bethesda, MD, USA).

### Lepr overexpression

The recombinant adenovirus was constructed containing the Lepr cDNA which encodes the 304-amino acid intracellular domain. Total RNA was extracted from the hypothalami of normal SD rats, reversely transcribed, and PCR-amplified by using oligonucleotides encompassing the C-terminal portion of the Lepr (amino acids 711-1162), with the sequences of the primers as 5′-TTC TGG CCA TCA ATT CCA TCG GTG C-3′ and 5′-GTC GAC TTA CAC AGT TAA GTC ACA CAT CTT-3′). This fragment was digested with *Eco*RV (Clontech, Mountain view, CA, USA) and *Sal*I (Clontech), and subcloned into an *Eco*RV/*Sal*I-digested expression vector. The construct containing *Lepr* cDNA was then ligated into the adenovirus vector pACCMV pLpA (Amaxa Biosystems, San Francisco, CA, USA). The recombinant adenovirus containing the *Lepr* cDNA under control of the cytomegalovirus (CMV) promoter (AdCMV-*lepr*) was then applied to dissociated hepatocytes, with the construct containing β-galactosidase cDNA (AdCMV-β-gal) serving as a negative control. The viral plaques were expanded into stocks containing 1–5 ×10^8^ plaque forming units/ml, and stored in EMEM supplemented with 7% FBS. Hepatocytes were transfected after cell attachment to 12-well plates by incubation with stocks of either AdCMV-Lepr or AdCMV-β-gal, for 1 h at a multiplicity of infection of 5.

### Exosome extraction and co-cultivation

We collected 50 ml blood from each subgroup of rats, both with and without metformin administration. Exosomes were extracted from sera using an extraction kit (Ribobio Co., Ltd., Guangzhou, China) according to the manufacturer’s instructions. Exosomes were then cocultured with BRL 3 A cells for 48 h or 2 weeks with exosome-supplemented medium replaced every other day. Cell lysates were collected and quantified for subsequent Western blotting analysis.

### Western blotting analysis

Cell or tissue lysates were collected and quantified following standard protocols. 20 μg protein samples were separated by 12% sodium dodecyl sulfate–polyacrylamide gel electrophoresis, and transferred to a nitrocellulose membrane. The membrane was blocked for 1.5 h with Tris-buffered saline containing Tween 20 (TBST) and 1% bovine serum albumin, at room temperature. This was followed by overnight incubation at 4 °C with the following primary antibodies, respectively: AMPK (1:1,000, rabbit mAb, cat# 5382 S, clone D63G4; 64KD; Cell Signaling Technology, Beverly, MA, US), phosphorylated AMPK (p-AMPK; 1:1,000, rabbit mAb, cat# 2537 S, clone 45F5; 62KD; Cell Signaling Technology), CCO subunit VIc (1:500, rabbit mAb, clone EPR9938, cat# ab150422; 12KD; Abcam), Lepr (1:2,000, rabbit pAb, cat# ab5593; 100KD; Abcam), cleaved Caspase-1 (1:1,000, rabbit mAb, clone E2G2I, cat# 89332; 22KD; Cell signaling technology), cleaved Caspase-3 (1:1,000, rabbit pAb, cat# pc679, 17KD; Millipore), Caspase-5 (1:1,000, rabbit pAb, cat# NB026564, 45KD; Thermo Fisher Scientific), Caspase-11 (1:1,000, rabbit pAb, cat# ab18741, 36KD/45KD; Abcam), cleaved Caspase-8 (1:1,000, rabbit pAb, cat# NB10056116, 17KD; Thermo Fisher Scientific), cleaved Caspase-9 (1:1,000, rabbit pAb, cat# 9507, 38KD; Cell Signaling Technology), interleukin (IL)-1β (1:1,000, mouse mAb, cat# MAB5011, clone 38123; 17KD; R&D Systems, Minneapolis, MN, US), IL-18 (1:1,000, mouse mAb, cat# MAB521, clone 69604; 18KD; R&D Systems), Gasdermin-D (GSDMD, 1:1,000, rabbit mAb, cat# ab219800, clone EPR20859; 53KD; Abcam), Gasdermin-E N-terminal (GSDME-N, 1:1,000, rabbit mAb, cat# ab215191, clone EPR19859; 55KD; Abcam), Bcl-2 (1:1,000, mouse mAb, cat# MAB8272, clone 625509; 24KD; R&D Systems), and β-actin (1:1,000, mouse mAb, cat# MAB8929, clone 937215; R&D Systems). Each membrane was washed three times with Tris-buffered saline Tween, then incubated with the corresponding horseradish peroxidase-conjugated goat anti-rabbit or anti-mouse IgG or IgM (1:2,000; Millipore) for 1 h at room temperature, followed by several washes with TBST (Sigma). The chemiluminescence signal was detected using ECL (GE Healthcare Bio-Sciences, Pittsburgh, PA, USA), and developed on X-ray film. Band intensities were quantified by Image Lab (Bio-Rad, Hercules, CA, USA), and the protein expression level was normalized to the β-actin level. Data are presented as the fold change in the logarithmic scale, in expression relative to control.

### Biochemical and quantitative analysis of rat blood samples

Rat blood from the tail vein was used to detect blood glucose levels with a blood glucose meter (Onetouch Ultra 2 Meter, LifeScan Inc., Indianapolis, IN, US). Total cholesterol (cat# MAK043) and triglycerides (cat# TR0100), alanine aminotransferase (ALT; cat# MAK052), aspartate aminotransferase (AST; MAK055), total protein and (cat# T1949) albumin content (cat# MAK124) were measured using quantitation kits (all from Sigma), according to the manufacturer’s instructions.

### Quantitative real-time reverse transcription-polymerase chain reaction (RT-PCR)

We isolated total RNA from cells using Trizol (Invitrogen), and reversely transcribed them to produce cDNA using an RNeasy Extraction Kit (Qiagen, Valencia, CA, US), according to the manufacturer’s instructions. The quantitative real-time RT-PCR was performed in an ABI Prism 7000 Sequence Detector (Applied Biosystems, Foster City, CA, US) using SYBR Green PCR Master Mix reagent as the detector, according to the manufacturer’s instructions. Rat primer sequences were as follows: *Lepr*, (forward) GAG TGA CTG GAG TTT ACC TCA A, (reverse) AAA AGG AAG CAT TGG ATC CAA C; *CCO*, (forward) AGT GCG AAG TGA TTT CAG AAT G, (reverse) ATA GTT CAG GAA CAC AGG TCA G; *Caspase-3*, (forward) GAT CCC GTG TAT TGT GTC AAT G, (reverse) CTG ACA GTT TTC TCA TTT GGC A; *Bcl-2*, (forward) TCG CGA CTT TGC AGA GAT GT, (reverse) CAA TCC TCC CCC AGT TCA CC; *β-actin*, (forward) CCA CCC GCG AGT ACA ACC TT, (reverse) CCC ACG ATG GAG GGG AAG AC. The target gene expression levels were normalized to the *β-actin* level using the comparative C_T_ method. Data are presented on a logarithmic scale as fold changes in expression relative to the control.

### Single-cell RNA sequencing

Total RNA was isolated from single hepatocytes isolated from each subgroup of rats. To generate complementary DNA (cDNA), RNA samples were mixed with reverse transcription master mix containing 0.05 μl RNase inhibitor and 0.33 μl SuperScript III Reverse Transcriptase per 1× volume, and incubated on a thermocycler at 50 °C for 30 min and 70 °C for 15 min. A 3′-poly-A tail was added to the cDNA in each sample by incubating it in a master mix (0.6 μl 10× PCR Buffer II, 0.36 μl 25 mM MgCl_2_, 0.18 μl 100 mM dATP, 0.3 μl Terminal Transferase, 0.3 μl RNase H, and 4.26 μl H_2_O per 1× volume) at 37 °C for 15 min, and inactivated at 70 °C for 10 min. Each sample was divided in 4, and a second round of PCR amplification was conducted for 9 cycles of 98 °C for 3 min, 67 °C for 1 min, followed by 72 °C for 6 min, with master mix (9 μl 10× High Fidelity PCR Buffer, 3.6 μl 50 mM MgSO_4_, 13.5 μl 2.5 mM each dNTP, 1.8 μl Platinum Taq DNA Polymerase, and 59.1 μl H_2_O per 1× volume). Samples were pooled and purified using Agencourt AMPure XP beads (Beckman Coulter Life Sciences, Indianapolis, IN, US) and eluted in 40 μl 1× low TE buffer. To shear the DNA using the Covaris S2 System (Gene Co. Ltd., Hong Kong, China), 1× low TE buffer and 1.2 μl shear buffer was added to each sample. Then, samples were end-polished at room temperature for 30 min with a master mix (40 μl 5× Reaction Buffer, 8 μl 10 mM dNTP, 8 μl End Polish Enzyme 1, 10 μl End Polish Enzyme 2, and 14 μl H_2_O per 1× volume). Only products passing quality control qPCR for β-actin were subjected to library construction. The products were then eluted in 36 μl low TE buffer. A dA-tail was added to each size-selected DNA by treating it with master mix (10 μl 5× Reaction Buffer, 1 μl 10 mM dATP, and 5 μl A-Tailing Enzyme I per 1× volume), and incubating it at 68 °C for 30 min and cooling it to room temperature. To label and distinguish each DNA sample for sequencing, barcode adaptors (ABI 5500XL 4464405, Applied Biosystems) were ligated to DNA using the SOLiD Fragment Library Enzyme Module (ABI 5500XL 4464413). Then, samples were purified twice using the Agencourt AMPure XP beads after barcoding, and eluted in 22 μl low TE buffer. Lastly, to quantify the amount of ligated DNA, we utilized a SOLiD Library TaqMan Quantitation Kit for quantitative PCR. Completed barcoded libraries were then subjected to emulsion PCR with template bead preparation, and sequenced on the ABI 5500XL. For clustering during analysis, a minimum of 1 was added to the matrix to eliminate any zeros, and the results were then log transformed and median polished. Then, we clustered them using agglomerative hierarchical clustering with average linkage with distance metric equal to 1 minus the Pearson correlation coefficient. For each row representing 8 subtypes of isolated hepatocytes, we calculated an FDR q-value and a normalized fold-change. We did this with the DEGexp function in the Bioconductor DEGseq package version 1.10.0, with q-values calculated using the Benjamini-Hochberg method [30]. RNA sequencing and digital gene expression profiling yielded an average of 4 to 5 million uniquely aligned reads per sample. Determination of reads per million (RPM) and log10 (RPM) were performed as previously described [8]. Data was deposited into the NCBI Sequence Read Archive (SRA) database (Accession Number: PRJNA926697).

### Measuring glucose uptake

To measure glucose uptake, upon isolation from HFD-fed Lepr WT rats and Lepr-KO rats, the hepatocytes were subject to *Lepr* transfection. At 30 min post transfection, in the presence of metformin, hepatocytes were incubated with 100 μg/mL 2-deoxy-2-[(7-nitro-2,1,3-benzoxadiazol-4-yl) amino]-D-glucose (2-NBDG; Cat# ab235976, Abcam) in glucose-free medium for 1 h, and the fluorescence was measured at excitation and emission wavelengths of 485 nm and 535 nm, respectively, according to the manufacturer’s instructions.

### Cytokine detection

Dissociated hepatocytes were cultured in 12-well plates, and the original medium in each of the 12-well plate’s wells was 1 ml. To detect IL-1β and IL-18, culture medium was collected and centrifuged to remove any cell debris, and the enzyme-linked immunosorbent assay (ELISA) kits acquired from Invitrogen were used to measure IL-1β (Cat# 88-6010-22) and IL-18 (Cat# KRC2341) concentrations according to the manufacturer’s instructions.

### Detecting telomerase activity and telomere length

The telomerase activity was evaluated by quantitative PCR for cell populations derived from each group. Derived cells were homogenized in CHAPS buffer (Calbiochem, San Diego, CA, USA), and centrifuged at 4 °C. Cell extracts were incubated in a solution containing reverse transcriptase reaction mix and Taq polymerase (TRAPEZE RT Telomerase Detection Kit; Millipore) at 30 °C for 30 min. HeLa cells were used as positive control, and serial dilutions of control template TSR8 were used for quantitation. CHAPS buffer in the absence of protein lysates was applied as a negative control. PCR cycling conditions were as follows: 1 cycle of 95 °C for 2 min, followed by 40 cycles of 94 °C for 15 s and 59 °C for 60 s. Data were collected at the 59 °C stage of each cycle. Quantitative measurements of telomere length were conducted by flow-fluorescence in situ hybridization (flow-FISH) for cell populations as indicated. Calibration of the flow cytometer, cell fixation, staining protocol and normalization were conducted using mouse lymphoma cells with known telomere length. Then, we washed 5 × 10^5^ cells (to be detected) and rat lymphoma cells with long telomeres in hybridization buffer, and resuspended them in hybridization solution containing formamide and 0.3 μg/ml FITC-conjugated pentose nucleic acid (PNA) probe. Control samples were incubated in hybridization solution in the absence of PNA probe. Lymphoma cells were distinguished from cell derivatives by immunostaining with CD45 antibody (Millipore). DNA content was established by propidium iodide staining. Cells were gated at G0/G1based on DNA content, and the telomeres’ fluorescence intensity was calculated as previously described [37]. Detections were conducted on the FACSCanto flow cytometer (Becton Dickinson; Franklin Lakes, NJ, US). Data are presented as the fold change in expression relative to the hepatocytes isolated from HFD-fed Lepr WT rats with control vector transfection in the absence of metformin.

### Statistical analysis

Data were expressed as mean ± standard deviation. All statistical analyses were performed on SPSS 23.0 (Statistics Package for the Social Sciences 23.0, Chicago, IL). One-way ANOVA followed by LSD tests was used to assess any statistical differences in the biochemical parameters among all groups. A two-tailed *p* value of less than 0.05 was considered statistically significant.

## Results

### Metformin reduced HFD-induced hepatic steatosis in Zucker lean rats and SD rats, but not in ZDF rats

**Fig. 1:**
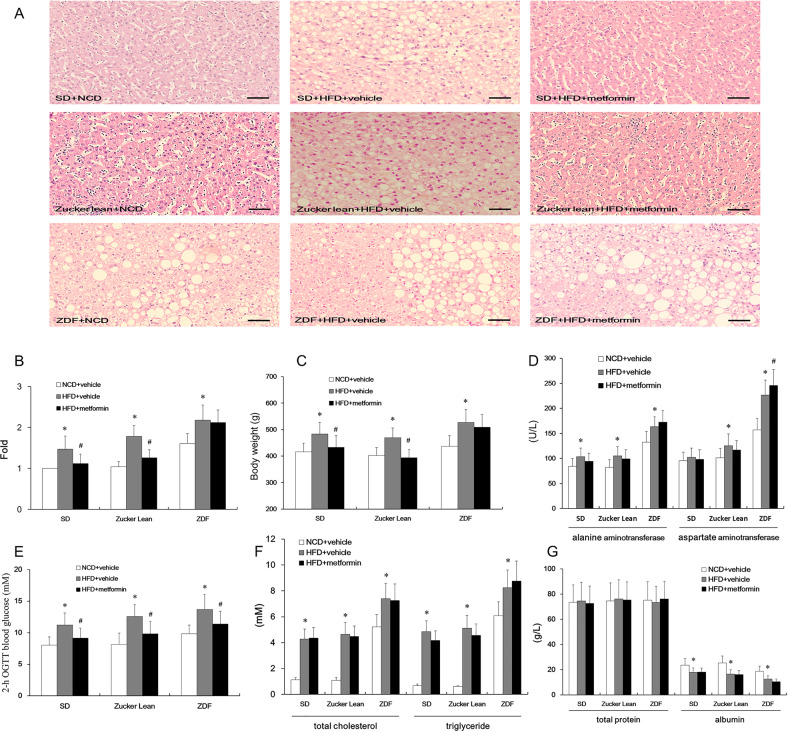
Liver histology and function assessment in Zucker diabetic fatty (ZDF) rats and controls (with and without 6 months of metformin administration). **A** HE staining. Scale bars, 25 μm. **B** Hepatocytes’ diameter multiples in high-fat diet induced NAFLD. *n* = 12. *p < 0.05 vs. normal chow diet (NCD) + vehicle; ^#^*p* < 0.05 vs. high-fat diet (HFD) + vehicle. **C** Body weight. *n* = 12. *p < 0.05 vs. NCD + vehicle; ^#^*p* < 0.05 vs. HDF + vehicle. **D** Detection of alanine aminotransferase (ALT) and aspartate aminotransferase (AST). *n* = 12. *p < 0.05 vs. NCD + vehicle; ^#^*p* < 0.05 vs. HFD + vehicle. **E** Detection of 2-h OGTT blood glucose. *n* = 12. *p < 0.05 vs. NCD + vehicle; ^#^*p* < 0.05 vs. HFD + vehicle. **F** Detection of blood total cholesterol and triglycerides. *n* = 12. *p < 0.05 vs. NCD + vehicle. **G** Quantitation of total protein and albumin in the peripheral blood. *n* = 12. *p < 0.05 vs. NCD + vehicle; ^#^*p* < 0.05 vs. HFD + vehicle.

We scrutinized the biochemical profiles of blood and histological samples by comparing the responses of ZDF rats and their healthy counterparts with HFD-induced hepatic steatosis to metformin treatment. Healthy rats fed an HFD diet also developed liver dysfunction at 8 months old, as reflected by a decrease in serum albumin and elevated serum alanine aminotransferase (ALT) and aspartate transaminase (AST) (Fig. 1A, D). Compared with Zucker lean rats and SD rats, only untreated ZDF rats showed patches of ballooning degeneration and fatty change with cytoplasmic microvacuolation present on hepatocytes (Fig. 1A). These changes became more pronounced in HFD-fed ZDF rats, where patches of the degenerated cells were surrounded by condensed hepatocytes (Fig. 1A). Although the glucose intolerance can be improved in HFD-fed rats, metformin resulted in no significant change in either their total blood cholesterol or their triglycerides (Fig. 1E, F). Likewise, the hepatic dysfunction was not affected by metformin treatment (Fig. 1D, G). As for HFD-induced NAFLD in Zucker lean rats and SD rats, metformin administration decreased the weight gain, as well as the diameter of the hepatocytes (Fig. 1A–C). In contrast, in ZDF rats, metformin treatment manifested neither weight nor liver pathology benefits, but did increase AST (Fig. 1A–D). These results indicated that long period of metformin treatment improved neither NAFLD status nor glucose metabolism in ZDF rats, as it had done in counterpart models.

### AMPK activation and hepatocyte death increased in Lepr-defective ZDF rats, and might have been exacerbated by metformin administration

**Fig. 2:**
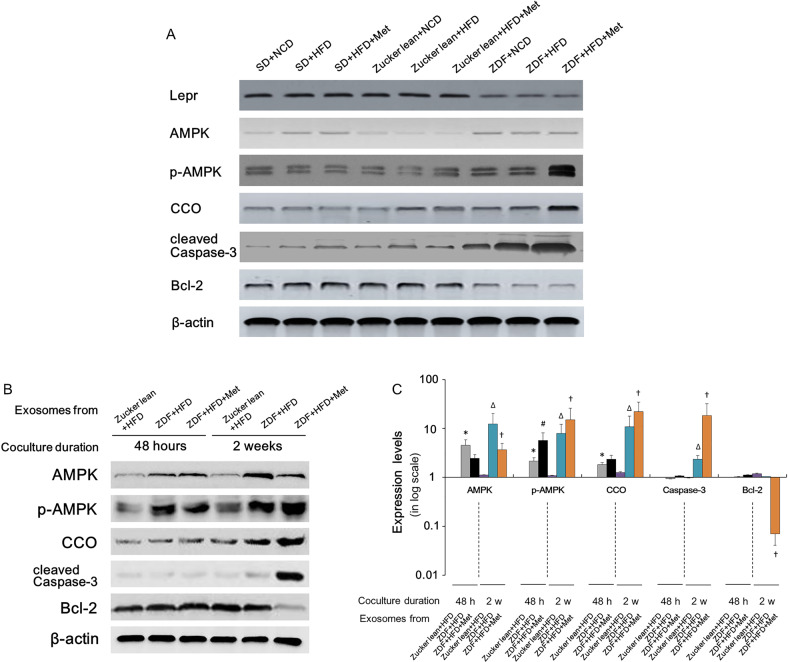
Western blotting analysis for proteins in rat livers and BRL 3 A cells co-cultured with blood-derived exosomes from ZDF and Zucker lean rats. **A** Western blotting analysis for the livers of SD, Zucker lean and ZDF rats at 6 months post different treatments, (as indicated). **B** BRL 3 A cells were subject to Western blotting analysis at 48 h and 2 weeks post exosome co-culture. **C** Quantitative analysis of **B**. Except for AMPK (*n* = 10) and p-AMPK (*n* = 10), *n* = 12. Band intensities in **B** were quantified via Image Lab (Bio-Rad, Hercules, CA, USA) and the protein expression level was normalized to the level of the β-actin. Data are presented on a logarithmic scale as the fold change in expression relative to controls (after coculture with exosomes extracted from 48 h HFD-fed Zucker lean rats in the absence of metformin; Zucker lean+HFD). *p < 0.05 vs. controls; ^#^*p* < 0.05 vs. cultures after coculture with exosomes extracted from 48 h HFD-fed ZDF rats in the absence of metformin; ZDF + HFD; ^Δ^*p* < 0.05 vs. cultures with exosomes extracted from 2-week HFD-fed Zucker lean rats in the absence of metformin (Zucker lean + HFD); ^†^*p* < 0.05 vs. cultures with exosomes extracted from 2-week HFD-fed ZDF rats in the absence of metformin (ZDF + HFD).

*Lepr* levels were quantified by Western blotting in each group, demonstrating that ZDF rats showed obvious *Lepr* defective, when compared with their healthy counterparts (Fig. 2A) (see the Supplementary Materials for the uncropped original Western blotting images mentioned in this article). In view of the distinctive effects of metformin on ZDF rats, this suggests that *Lepr* dysfunction be considered. After 6 months of metformin treatment, the expression of phosphorylated AMPK (p-AMPK) and cytochrome C oxidase (CCO, a mitochondrial enzyme involved in the electron transport chain that promotes blood glucose and lipid metabolism [38]) increased significantly in the ZDF rats, while the Zucker lean rats and SD rats did not show such an increase. This indicated that metformin could bring about AMPK and CCO overreaction in the absence of *Lepr* (Fig. 2A). Moreover, Caspase-3 activation led to the destruction of structural proteins in hepatocytes, resulting in cell death [39]. On the other hand, the overexpression of anti-apoptotic factor Bcl-2 has a protective effect against liver cell injury [39]. We assessed both Caspase-3 and Bcl-2 levels to investigate whether *Lepr* deficiency would affect programmed cell death in hepatocytes. ZDF rats exhibited higher expression levels of Caspase-3 and lower levels of Bcl-2; whereas after metformin treatment, hepatocyte death increased significantly in ZDF rats, but not in the other two counterparts with normal *Lepr* expression. This further suggests causality between *Lepr* deficiency and liver damage after metformin treatment (Fig. 2A).

To assess how liver cell contents from ZDF rats and control models could regulate metabolic pathways in liver cell lines, we co-cultured exosomes extracted from ZDF rats and controls with rat hepatic cell line BRL 3 A. During the first 48 h, AMPK was obviously phosphorylated when rat liver BRL 3 A cells were co-cultured with exosomes derived from HFD-fed Zucker lean rats (Fig. 2). Of note, the p-AMPK levels increased significantly in BRL 3 A cells when BRL 3 A cells were co-cultured with exosomes derived from HFD-fed ZDF rats, at both 48 h and 2 weeks post co-culture (Fig. 2B, C). In marked comparison, BRL 3 A cells showed AMPK over-phosphorylation when co-cultured with exosomes derived from HFD-fed metformin-administered ZDF rats. This suggested AMPK over-activation, in which process similar patterns were revealed when the co-culturing lasted for 48 h and 2 weeks (Fig. 2B, C). After co-culture for 48 h, exosomes derived from HFD-fed metformin-treated ZDF rats could induce CCO overexpression in BRL 3 A cells (Fig. 2B, C). Moreover, 2 weeks of co-culture with exosomes derived from HFD-fed metformin-treated ZDF rats resulted in higher CCO upregulation in BRL 3 A cells (Fig. 2B, C). These results suggested that the hypoglycemic and lipid-lowering effects of metformin were not immediate, and needed at least 2 weeks to develop in diabetic rats. On the other hand, the overexpression of pro-survival factor Bcl-2 has a protective effect against liver cell injury [28]. We assessed both the Caspase-3 and Bcl-2 levels to investigate whether metformin would affect programmed cell death. ZDF rat-derived exosomes, regardless of metformin administration to rats, did not induce the expression of Caspase-3 and Bcl-2 in BRL 3 A cells at 48 h post co-culture (Fig. 2B, C). When co-cultured with serum exosomes derived from HFD-fed metformin-treated ZDF rats for 2 weeks, BRL 3 A cells exhibited a significant increase in Caspase-3 expression and a decrease in Bcl-2 level. This suggests that *Lepr* deficiency combined with metformin treatment played a pivotal role in increasing pro-apoptotic factors in exosomes. As expected, the exosomes derived from HFD-fed metformin-treated Zucker lean rats did not promote programmed cell death in BRL 3 A cells (Fig. 2B, C). These results suggest that metformin promotes cell death in ZDF rat livers after 2 weeks of treatment, thus confirming metformin’s unfavorable effects on hepatocytes in the milieu of Lepr deficiency.

### Lepr deletion mitigates metformin’s benefits, while inducing hepatic cell death and liver damage

**Fig. 3:**
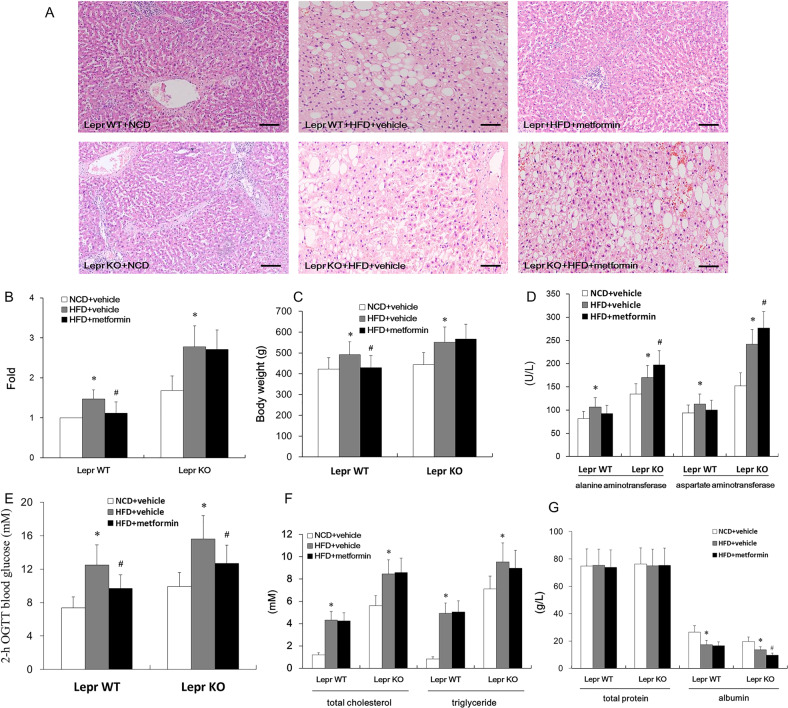
Liver histology and function assessment in leptin receptor (Lepr)-knockout rats with and without 6 months of metformin administration under the influence of a high-fat diet (HFD). **A** HE staining. Scale bars, 25 μm. **B** Diameter of the hepatocytes in **A**. *n* = 12. **C** Body weight. *n* = 12. **D** Quantitation of alanine aminotransferase (ALT) and aspartate aminotransferase (AST) in serum. *n* = 12. **E** Quantitation of 2-h OGTT blood glucose. *n* = 12. **F** Detection of blood total cholesterol and triglycerides. *n* = 12. **G** Quantitation of total protein and albumin in the peripheral blood. *n* = 12. *p < 0.05 vs. NCD + vehicle; ^#^*p* < 0.05 vs. HFD + vehicle.

In order to explore whether the hepatic injury in HFD-fed metformin-treated ZDF rats is directly affected by *Lepr* deficiency, we analyzed the serological indexes and liver pathology of *Lepr* wild type (WT) rats and *Lepr* knock-out (KO) rats with HFD-induced NAFLD, with and without metformin administration. In all rats, without metformin treatment, HFD led to higher body weights, higher levels of fat deposits, liver enzymes, blood glucose, cholesterol and triglycerides (Fig. 3). No significant difference due to 6 months of metformin treatment was noted in the levels of total cholesterol, triglycerides or total proteins, in any of the rats with HFD (Fig. 3F, G). In addition to the blood glucose-lowering effects of metformin, histology of liver tissues showed that metformin could reduce hepatocyte diameter and body weight in *Lepr* WT rats with NAFLD phenotypes (Fig. 3A–C, E). However, the beneficial effects such as improved liver steatosis and weight gain dropped when *Lepr* was deleted (Fig. 3A–C). Furthermore, *Lepr* knockout nearly eliminated metformin’s benefits on liver steatosis, and even induced an increase in ALT and AST levels, and a decrease in albumin. We did not observe this deterioration in liver enzyme levels in metformin-treated *Lepr* WT rats (Fig. 3D, G). This indicated that the *Lepr* deficiency was indeed responsible for the liver damage caused by long-term metformin administration. In other words, functional Lepr is necessary for metformin to be beneficial in NAFLD treatment.

**Fig. 4:**
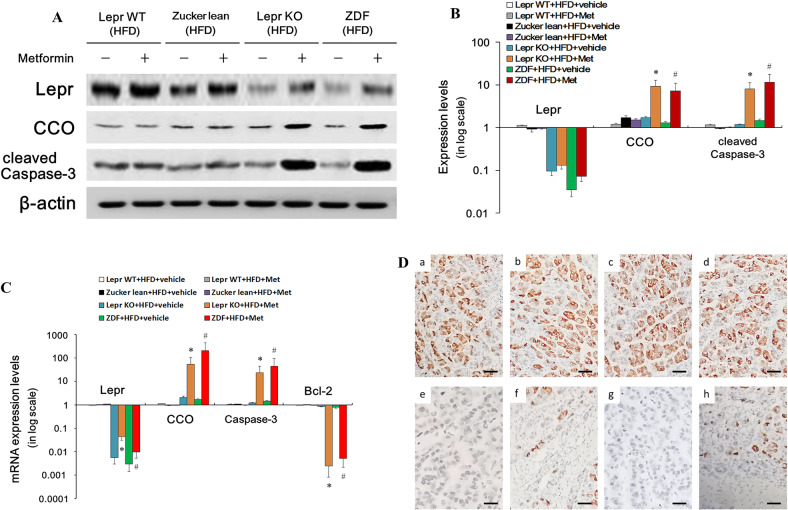
Protein and mRNA levels of Lepr, cleaved Caspase-3, Bcl-2 and CCO in the livers of HFD-fed Lepr-WT, Zucker lean, Lepr-KO and ZDF rats, with and without metformin treatment. **A** Western blotting for proteins derived from freshly harvested liver tissue. **B** Quantitative analysis of **A**. *n* = 12. Band intensities in **A** were quantified in Image Lab (Bio-Rad) and the protein expression level was normalized to the β-actin level. Data are presented on a logarithmic scale as the fold change in expression relative to Zucker lean + HFD. **C** Quantitative real-time RT-PCR analysis for mRNA of the liver tissues (as indicated). *n* = 12. **D** Immunohistochemical analysis for Lepr. a Lepr WT + HFD + vehicle; b Lepr WT + HFD + Met; c Zucker lean + HFD + vehicle; d Zucker lean+HFD + Met; e Lepr-KO + HFD + vehicle; f LeprKO+HFD + Met; g ZDF + HFD + vehicle; h ZDF + HFD + Met. Scale bars 30 μm. *p < 0.05 vs. Lepr-KO + HDF + vehicle; ^#^*p* < 0.05 vs. ZDF + HDF + vehicle.

We investigated whether leptin sensitivity could alter the effects of 6-month administration of metformin on metabolic pathways and the programmed cell death process. To do so, protein and mRNA level of *Lepr*, cleaved Caspase-3 and CCO were measured in HFD-fed *Lepr*-WT rats, *Lepr-*KO rats, Zucker lean rats and ZDF rats, with and without metformin administration (Fig. 4). Both mitochondrial metabolic marker CCO and cell death marker cleaved Caspase-3 were upregulated in *Lepr*-deficient rats (including *Lepr*-KO rats and ZDF rats) after 6 months of metformin treatment (Fig. 4A, B). Similar expression profiles were confirmed on the transcriptional level (Fig. 4C). In addition, the mRNA level of pro-survival factor Bcl-2 was downregulated in *Lepr*-deficient rats after metformin treatment (Fig. 4C). *Lepr*’s expression status in the livers of *Lepr*-KO rats, Lepr WT rats, ZDF rats and Zucker lean rats was also visualized via immunohistochemical staining (Fig. 4D). ZDF rats and *Lepr-*KO rats showed similar, lower-than-normal expression profile for *Lepr*, verifying that ZDF rats also had defective *Lepr* defectives (Fig. 4A–D). This finding confirmed our inference that *Lepr* is necessary for metformin to be beneficial to NAFLD treatment. Taken together, these results revealed that *Lepr* defective promotes hepatic cell death, resulting in liver injury when such individuals with NAFLD are treated with metformin.

### Metformin’s pyroptotic effect on hepatocytes is associated with overactivation of AMPK and CCO during Lepr defective

**Fig. 5:**
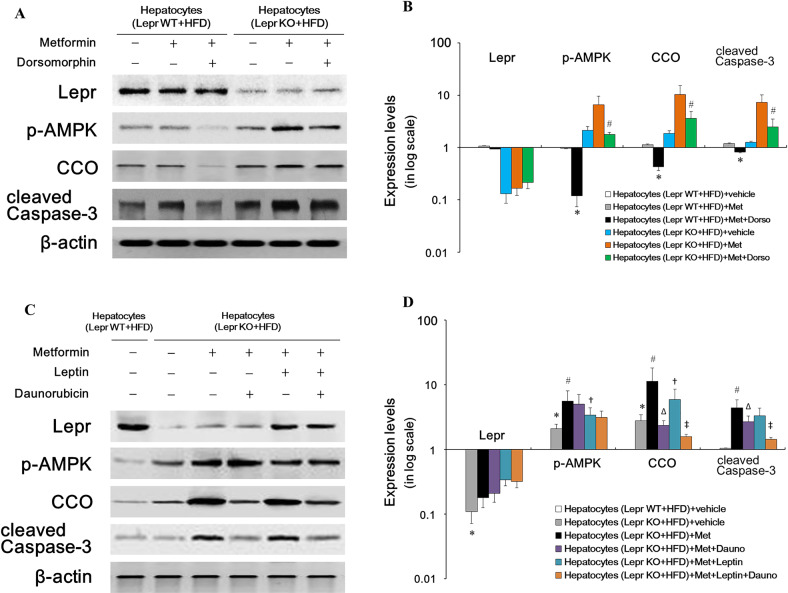
The p-AMPK and CCO are downstream effectors of *Lepr*. Purified hepatocytes were cultured in the medium either with or without metformin (10 μM), or AMPK inhibitor Dorsomorphin for 96 h, before they were harvested and subjected to Western blotting analysis. Meanwhile, in a subset of the experiment, hepatocytes were pre-treated with cytochrome C oxidase (CCO) inhibitor Daunorubicin for 24 h, before switching to medium supplemented with metformin (10 μM) and leptin (10 μM; Sigma) for 72 h. After that, hepatocytes were harvested and subjected to Western blotting analysis. **A** Western blotting images of hepatocytes, with and without metformin/or dorsomorphin. **B** Quantitative analysis of **A**. Except for p-AMPK (*n* = 10), *n* = 12. Band intensities in **A** were quantified in Image Lab (Bio-Rad), and the protein expression level was normalized to the β-actin level. Data are presented on a logarithmic scale as the fold change in expression relative to the hepatocytes derived from HFD-fed Lepr WT rats in the absence of metformin and dorsomorphin. *p < 0.05 vs. hepatocytes derived from HFD-fed Lepr WT rats in the absence of metformin or dorsomorphin; ^#^*p* < 0.05 vs. hepatocytes derived from HFD-fed Lepr-KO rats in the absence of dorsomorphin. **C** Western blotting images of hepatocytes, with and without metformin, leptin or daunorubicin. **D** Quantitative analysis of **C**. Except p-AMPK (*n* = 10), *n* = 12. Band intensities in **C** were quantified in Image Lab (Bio-Rad), and the protein expression level was normalized to the β-actin level. Data are presented on a logarithmic scale as the fold change in expression relative to hepatocytes derived from HFD-fed Lepr WT rats in the absence of metformin, leptin or daunorubicin. *p < 0.05 vs. hepatocytes derived from HFD-fed Lepr WT rats in the absence of metformin, leptin or daunorubicin; ^#^*p* < 0.05 vs. hepatocytes derived from HFD-fed Lepr-KO rats in the absence of metformin, leptin or daunorubicin; ^Δ^*p* < 0.05 vs. hepatocytes derived from HFD-fed Lepr-KO rats in the absence of leptin or daunorubicin; ^†^*p* < 0.05 vs. hepatocytes derived from HFD-fed Lepr-KO rats in the absence of leptin; ^‡^*p* < 0.05 vs. hepatocytes derived from HFD-fed Lepr-KO rats in the absence of daunorubicin.

To further explore the *Lepr* signal transduction pathway involving both AMPK and CCO in relation to metformin, we used inhibitors for the two enzymes. AMPK was activated in purified hepatocytes derived from HFD-fed *Lepr*-KO rats in the presence of metformin, and expression levels of p-AMPK, cleaved Caspase-3 and CCO showed the same trend (Fig. 5A, B). Dorsomorphin, an AMPK inhibitor, inhibited not only the overexpression of p-AMPK, but also that of CCO and cleaved Caspase-3 mediated by metformin, but with minimal effect on *Lepr* expression; Even in hepatocytes derived from Lepr WT rats, dorsomorphin can lower the p-AMPK and CCO levels in the presence of metformin. This indicates that p-AMPK and CCO are downstream effectors of *Lepr* (Fig. 5A, B). Meanwhile, in hepatocytes isolated from the livers of *Lepr-KO* rats, in the presence of metformin, CCO inhibitor daunorubicin at 1 μM could decrease expression levels of CCO and cleaved Caspase-3, with minimal influence on p-AMPK. This suggests that p-AMPK acted upstream of CCO (Fig. 5C, D). Furthermore, the simultaneous decrease in cleaved Caspase-3 expression also implied that there was a positive correlation between CCO and cleaved Caspase-3 (*r* = 0.972, *p* < 0.05). In the presence of metformin and the absence of daunorubicin, leptin, which binds to Lepr, slightly increased Lepr expression, subsequently downregulating p-AMPK and CCO levels. Meanwhile, in the presence of both metformin and daunorubicin, p-AMPK downregulation mediated by leptin was minimally affected, and CCO level was further decreased by daunorubicin (Fig. 5C, D). Therefore, we were able to pave a pathway underlying hepatocyte damage, during which process, Lepr defective caused metformin-mediated AMPK phosphorylation and subsequent CCO activation. This resulted in Caspase-3 activation and programmed cell death.

### Metformin administration led to an overexpression of genes involved in glucose catabolism in Lepr-defective rat-derived hepatocytes

**Fig. 6:**
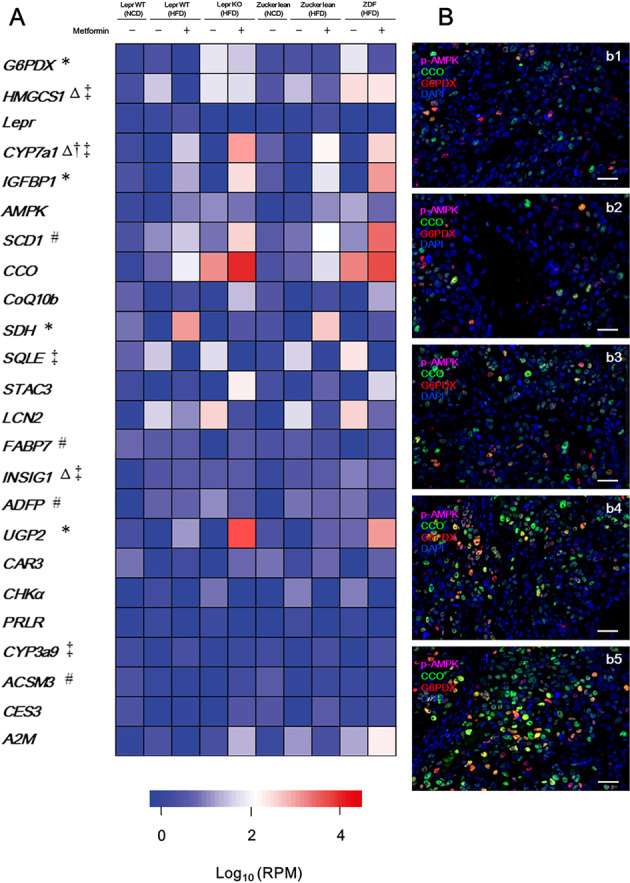
Single-cell RNA sequencing and an immunofluorescence study of hepatocytes isolated from rats (as indicated). **A** Single-cell RNA sequencing: 24 genes with the greatest difference between metformin-treated Lepr WT rats and Lepr-KO rats. *n* = 12. *Glucose metabolism-involved genes; ^#^Fatty acid metabolism-involved genes; ^Δ^Cholesterol metabolism-involved genes; ^†^Bile acid metabolism-involved genes; ^‡^Sterol metabolism-involved genes. **B** Immunofluorescence study on in-vitro cultured hepatocytes isolated from normal chow diet (NCD)-fed *Lepr* wild type (WT) rats without metformin (b1), high-fat diet (HFD)-fed *Lepr* WT rats without (b2) or with (b3) 6 months of metformin administration, and HFD-fed *Lepr* knock-out (KO) rats without (b4) or with (b5) 6 months of metformin administration. Scale bars, 50 μm. G6PDX, glucose-6-phosphate dehydrogenase X-linked; HMGCS1, 3-hydroxy-3-methylglutaryl-coenzyme A synthase 1 Lepr, leptin receptor; CYP7a1, cytochrome P450 (family 7, subfamily a, polypeptide 1); IGFBP1, Insulin-like growth factor-binding protein 1; AMPK, adenosine monophosphate (AMP)-activated protein kinase; SCD1, Stearoyl-coenzyme A desaturase 1; CCO, cytochrome C oxidase; CoQ10b, Coenzyme Q10 homolog B; SDH, serine dehydratase; SQLE, Squalene epoxidase; STAC3, SH3- and cysteine-rich domain 3; LCN2, lipocalin 2; FABP7, fatty acid-binding protein 7 (brain); INSIG1, insulin-induced gene 1; ADFP, adipose differentiation-related protein; UGP2, uridine diphosphate (UDP)-glucose pyrophosphorylase 2; CAR3, carbonic anhydrase 3; CHKα, choline kinase α; PRLR, prolactin receptor; CYP3a9, cytochrome P450, family 3, subfamily a, polypeptide 9; ACSM3, acyl-CoA synthetase medium-chain family member3; CES3, carboxylesterase 3; A2M, alpha-2-macroglobulin.

Next, we examined metformin’s effect on the gene regulation involved in energy metabolism. We quantified the expression levels of the genes involved in the metabolic pathways of glucose, fatty acid, cholesterol, bile acid and sterol (Fig. 6A). Among them, as indicated by single-cell RNA sequencing, genes involved in glucose catabolism, including *glucose-6-phosphate dehydrogenase X-linked* (*G6PDX*), *insulin-like growth factor-binding protein 1* (*IGFBP1*), *serine dehydratase* (*SDH*) and *uridine diphosphate-glucose pyrophosphorylase 2* (*UGP2*) exhibited an upregulated expression profile in hepatocytes derived from HFD-fed metformin-treated *Lepr-KO* rats (Fig. 6A). This suggests that these genes’ aberrant hyperactivation might have led to the over-consumption of cellular energy, which would have resulted in pyroptosis or apoptosis (which will be verified in downstream studies) of hepatocytes. In comparison with *Lepr* WT rat-derived hepatocytes, under the influence of 6 months of metformin administration, hepatocytes from *Lepr-KO* rats exhibited the most robust expression of p-AMPK, CCO and G6PDX (Fig. 6B). This verified that highly activated mitochondrial energy metabolism is a consequence of *Lepr* defective in the presence of metformin.

### Lepr re-expression can rescue Lepr-defective hepatocytes from metformin-induced pyroptosis

**Fig. 7:**
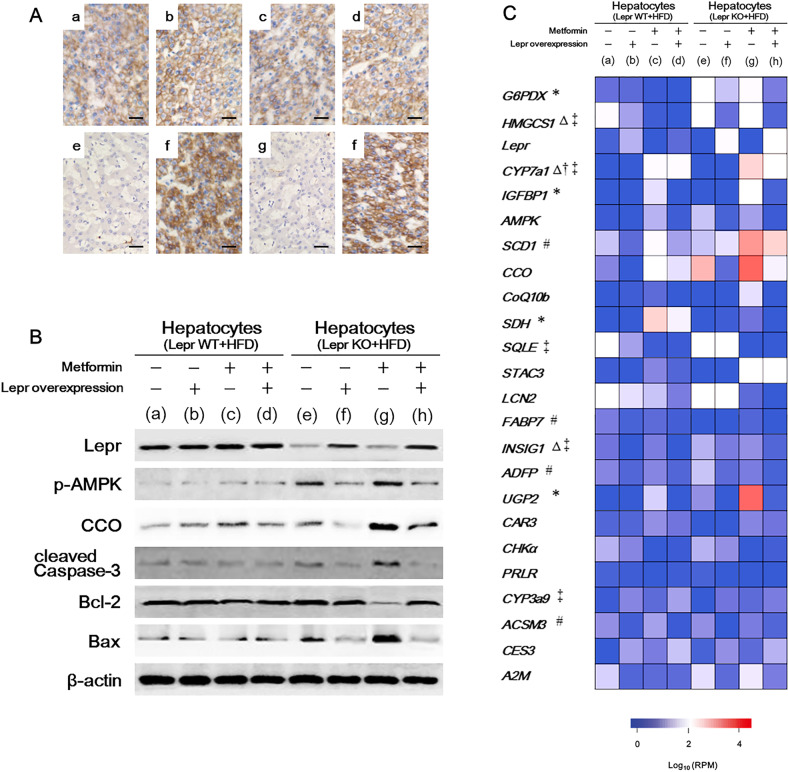
Leptin receptor (Lepr) overexpression can rescue hepatocytes from the hyper-activation of glucose metabolism-involved genes, containing *G6PDX*, *IGFBP1*, *SDH* and *UGP2*. **A** We conducted overexpression of *Lepr* in hepatocytes isolated from high-fat diet (HFD)-fed Lepr-knockout (Lepr-KO) rats and Lepr wild type (Lepr WT) rats. This was followed by immunostaining on Lepr at 96 h post transfection in both the presence and the absence of metformin. Scale bars, 30 μm. a, b hepatocytes isolated from HFD-fed Lepr WT rats were transfected with Lepr construct (b) or control vector (a) in the absence of metformin; c-d, hepatocytes isolated from HFD-fed Lepr WT rats were transfected with lepr construct (d) or control vector (c), followed by metformin treatment for 96 h; e-f, hepatocytes isolated from HFD-fed Lepr-KO rats were transfected with Lepr construct (f) or control vector (e) in the absence of metformin; g-h, hepatocytes isolated from HFD-fed Lepr-KO rats were transfected with Lepr construct (h) or control vector (g), followed by metformin treatment for 96 h. **B** Western blotting analysis for a-h in **A**. **C** Single-cell RNA sequencing for a–h in **A** (the 24 genes with the widest disparities between metformin-treated Lepr WT hepatocytes and Lepr-KO hepatocytes). *n* = 12. *Glucose metabolism-involved genes; ^#^Fatty acid metabolism-involved genes; ^Δ^Cholesterol metabolism-involved genes; ^†^Bile acid metabolism-involved genes; ^‡^Sterol metabolism-involved genes. G6PDX, glucose-6-phosphate dehydrogenase X-linked; HMGCS1, 3-hydroxy-3-methylglutaryl-coenzyme A synthase 1 swqazXEDwseqadxzLepr, leptin receptor; CYP7a1, cytochrome P450 (family 7, subfamily a, polypeptide 1); IGFBP1, Insulin-like growth factor-binding protein 1; AMPK, adenosine monophosphate (AMP)-activated protein kinase; SCD1, Stearoyl-coenzyme A desaturase 1; CCO, cytochrome C oxidase; CoQ10b, Coenzyme Q10 homolog B; SDH, serine dehydratase; SQLE, Squalene epoxidase; STAC3, SH3- and cysteine-rich domain 3; LCN2, lipocalin 2; FABP7, fatty acid-binding protein 7 (brain); INSIG1, insulin-induced gene 1; ADFP, adipose differentiation-related protein; UGP2, uridine diphosphate (UDP)-glucose pyrophosphorylase 2; CAR3, carbonic anhydrase 3; CHKα, choline kinase α; PRLR, prolactin receptor; CYP3a9, cytochrome P450, family 3, subfamily a, polypeptide 9; ACSM3, acyl-CoA synthetase medium-chain family member3; CES3, carboxylesterase 3; A2M, alpha-2-macroglobulin.

**Fig. 8:**
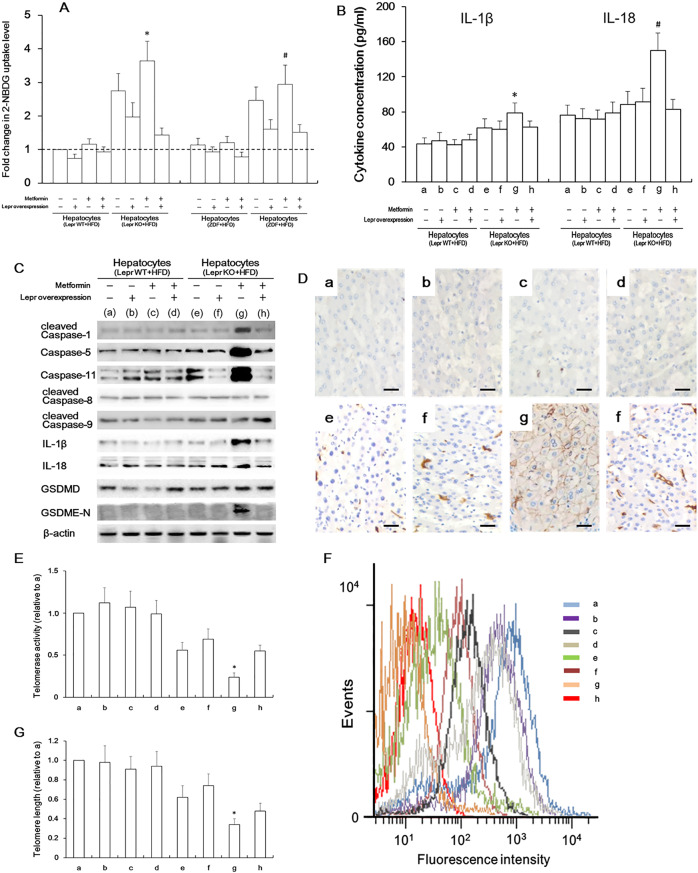
Lepr overexpression can rescue Lepr-KO rats’ hepatocytes from metformin-induced pyroptosis. **A** To measure glucose uptake, upon isolation from HFD-fed Lepr WT rats and Lepr-KO rats, hepatocytes were subject to *Lepr* transfection. *n* = 10. At 30 min post transfection, in the presence of metformin, the hepatocytes were incubated with 100 mg/mL 2-deoxy-2-[(7-nitro-2,1,3-benzoxadiazol-4-yl) amino]-D-glucose (2-NBDG) in glucose-free medium for 1 h. The fluorescence was measured at excitation and emission wavelengths of 485 nm and 535 nm, respectively. *p < 0.05 vs. HFD-fed Lepr-KO rat-derived hepatocytes in the presence of *Lepr* transfection and metformin; ^#^*p* < 0.05 vs. HFD-fed ZDF rat-derived hepatocytes in the presence of *Lepr* transfection and metformin. **B** Enzyme-linked immunosorbent assay (ELISA). *n* = 12. Culture medium was collected and centrifuged to remove cell debris (the original medium in each well of a 12-well plate was 1 ml). a, b hepatocytes isolated from HFD-fed Lepr WT rats were transfected with Lepr construct (b) or control vector (a) in the absence of metformin; c-d, hepatocytes isolated from HFD-fed Lepr WT rats were transfected with Lepr construct (d) or control vector (c), followed by metformin treatment for 96 h; e, f hepatocytes isolated from HFD-fed Lepr-KO rats were transfected with Lepr construct (f) or control vector (e) in the absence of metformin; g-h, hepatocytes isolated from HFD-fed Lepr-KO rats were transfected with Lepr construct (h) or control vector (g), followed by metformin treatment for 96 h. *p < 0.05 vs. h in IL-1β detection; ^#^*p* < 0.05 vs. h in IL-18 detection. a–h are the same as those in **C**–**G**. **C** Western blotting analysis for proteins (as indicated) in hepatocytes isolated from HFD-fed Lepr-knockout (Lepr-KO) rats and Lepr wild type (Lepr WT) rats at 96 h post transfection in the presence and absence of metformin. **D** immunostaining on IL-1β was performed at 96 h post transfection in either the presence or the absence of metformin. Scale bars, 25 μm. **E** Detection of telomerase activity in a–h. *n* = 16. *p < 0.05 vs. h. **F** Telomere length reflected by telomere fluorescence intensities were measured by flow-fluorescence in situ hybridization (flow-FISH). **G** Quantification of **F**. *n* = 16. *p < 0.05 vs. h.

To further demonstrate the necessity of *Lepr* in hepatocyte survival in the presence of metformin, we overexpressed *Lepr* in hepatocytes isolated from HFD-fed *Lepr-KO* rats and *Lepr* WT rats (Fig. 7A). Consequently, in *Lepr-KO* hepatocytes, the changes in expression levels of p-AMPK, CCO, cleaved Caspase-3, BAX and Bcl-2 induced by metformin were reversed by overexpression of *Lepr* (Fig. 7B). Single-cell RNA sequencing also showed a recovery in the expression of glucose metabolism-involved genes in the hepatocytes isolated from HFD-fed metformin-treated *Lepr-KO* rats at 96 h post *Lepr* transfection in the presence of metformin. This included *G6PDX*, *IGFBP1*, *SDH* and *UGP2* (Fig. 7C). Furthermore, we noticed an increase in 2-NBDG uptake in hepatocytes isolated from ZDF rats and *Lepr-KO* rats, in comparison with control rats in the presence of metformin (Fig. 8A). This indicated glucose uptake hyperfunction. Cytokine IL-1β and IL-18 were also significantly upregulated in metformin-treated hepatocytes isolated from HFD-fed *Lepr-KO* rats. We found that this was reversed by the complementary expression of *Lepr* (Fig. 8B), suggesting that pyroptosis may be the underlying cause of hepatic cell death. Consistently, at the post-transcriptional level, the expression of key players involved in pyroptosis, IL-1β, IL-18, caspase-1 and gasdermin E N-terminal (GSDME-N) were all significantly upregulated only in metformin-treated *Lepr-KO* hepatocytes. Their levels were restored by the complement expression of *Lepr* (Fig. 8C, D). In addition, we found that the expression of Caspase-5 and Caspase-11 was regulated in a similar manner. This suggested that both the canonical and non-canonical pathways of pyroptosis were involved in the metformin-induced death of *Lepr*-deficient hepatocytes [40]. Telomerase length and activity are also key indicators of cell survival and pyroptosis. In our study, the telomerase activity (Fig. 8E) and telomere length (Fig. 8F, G) were significantly reduced in metformin-treated *Lepr-KO* hepatocytes, and were partially restored by the complementary *Lepr* expression (Fig. 8E–G). On the other hand, the levels of cleaved Caspase-8 and Caspase-9 in the *Lepr*-deficient hepatocytes showed little effect from either metformin treatment or *Lepr* overexpression. This suggested that apoptosis was not a main factor contributing to metformin’s effect (Fig. 8C). Altogether, these results showed that metformin-induced liver damage mediated by *Lepr* defective was likely triggered by pyroptosis. In the presence of metformin, *Lepr*-deficient hepatocytes experienced a temporary increase in glucose uptake, followed by pyroptosis. Complementing *Lepr* expression in *Lepr*-deficient hepatocytes could restrain the hyper-activation of glucose metabolism, subsequently rescuing hepatocytes from metformin-mediated pyroptosis.

## Discussion

Sixty years of studies since the discovery of metformin have provided solid evidence for its beneficial roles in regulating metabolic processes. In a particular condition (e.g., NAFLD), long-term metformin treatment may induce hepatotoxicity, as indicated by the hepatic enzyme elevation and occurrence of hepatitis [14–18]. In clinical settings, metformin is often advised against, or used with caution, among diabetic patients with hepatitis or abnormal liver function, but the underlying mechanism remains unexplained. *Lepr* deficiency and leptin resistance have been widely studied in CNS and adipose tissue to explore their roles in the pathogenesis of metabolic disorders. Animal studies indicate that metformin’s therapeutic effect on NAFLD may be intertwined with leptin/Lepr signaling. It has been reported that metformin ameliorates hepato-steatosis and liver dysfunction in ob/ob mice, and in a leptin-deficient mouse model [41]. Also, in our previous study we reported that 6 months of metformin treatment could significantly lower blood glucose levels in *Lepr*-deficient ZDF rats, while neither blood lipid profiles, liver enzyme levels nor hepatocyte degeneration were alleviated [36]. Thus, we hypothesized that metformin’s therapeutic effect on NAFLD depends on leptin/Lepr signaling pathway. In subsequent experiments, we compared the therapeutic efficacy of metformin on HFD-induced NAFLD from *Lepr* WT rats and *Lepr*-deficient rats, respectively. As expected, our data showed that metformin could only alleviate NAFLD in rats with functional Lepr; whereas metformin promoted the pyroptosis of NAFLD hepatocytes when Lepr was dysfunctional in *Lepr*-deficient rats. These results suggest that metformin’s efficacy on NAFLD patients with T2D might be dependent on leptin sensitivity, or the functional binding between leptin and its receptor. Altogether, our study provides in vivo medical evidence of the pharmacology and pharmacokinetics of metformin in treating NAFLD and other conditions, opening new avenues to potential human clinical trials of such treatments.

Our study reveals new insight into Lepr’s roles in liver metabolism. Defective Lepr expression in the hepatocytes of ZDF rats eliminates the benefits of metformin treatment in weight loss and hepatic lipid regulation. It also leads to the deterioration of liver function and damage to the hepatic histological structure after long-term metformin administration. We observed similar phenomena in *Lepr-KO* rats, verifying that Lepr defect is detrimental to metformin treatment for NAFLD livers. From another perspective, this is also in line with the potent lipolytic effects of Lepr signaling pathway, as demonstrated by clinical trials [42, 43]. Consistent with our study, other studies have also shown that hepatic steatosis can be reduced and hepatocytes rescued by Lepr re-expression [44, 45]. Furthermore, we explored whether Lepr defect in hepatocytes could likewise respond independently to metformin. To exclude the effects of neurohumoral regulation or hepatic interstitial cells, we cultured purified hepatocytes derived from HFD-fed *Lepr* WT rats and *Lepr*-KO rats in vitro with metformin, and the individual hepatocytes showed increased expression levels of pyroptosis-related genes. This is consistent with the results in vivo. In vitro experiments confirmed that Lepr defect in hepatocytes underlays the hepatic damage induced by long-term metformin treatment, and this was independent of leptin/Lepr signaling from neurohumoral regulation governed by CNS. Although there was no original *Lepr* defect, BRL 3 A cells co-cultured with exosomes derived from the peripheral blood of HFD-fed metformin-treated *Lepr*-defective rats also showed AMPK overactivation. This is consistent with the results based on *Lepr*-defective liver tissue. Additionally, we found the upregulation of CCO and cleaved Caspase-3, and the downregulation of pro-survival factor Bcl-2 in BRL 3 A, at both 48 h and 2 weeks post co-culture with the aforementioned exosomes. This observation verified that Lepr defect and metformin treatment can affect the properties of circulating exosomes, which may promote hepatic damage via circulation, and may also be replenished by injured hepatocytes.

It has been reported that high CCO levels might be associated with programmed cell death [46]. In our study, we showed that CCO expression was positively correlated with that of cleaved Caspase-3 (*r* = 0.972, *p* < 0.05), a key effector in mediating cell death. CCO is mainly involved in the mitochondrial electron transport chain where it facilitates ATP synthesis from protons, and is produced primarily during glucose and lipid catabolism. In our study, in the presence of HFD induction, long-term metformin administration caused both hepatic cells and tissues derived from *Lepr-KO* rats to exhibit a higher CCO expression level than those from *lepr* WT rats. This indicated that Lepr may negatively regulate CCO to balance glucose catabolism and maintain metabolic homeostasis in hepatocytes in the presence of metformin. Meanwhile, we demonstrated that Lepr overexpression in *Lepr*-deficient hepatocytes reduces metformin-mediated CCO hyper-activation, not only at that transcription level, but also at the post-transcriptional protein level. Moreover, single-cell RNA sequencing of key glucose metabolic genes showed that *G6PDX*, *IGFBP1*, *SDH* and *UGP2* were hyper-activated, resulting in over-consumption of cellular energy followed by cell death. Consistent with our results, other studies have reported that disruption of hepatic Lepr signaling can change insulin sensitivity and glucose tolerance, leading to fat accumulation in the liver [45, 47]. In our study, in hepatocytes isolated from the livers of HFD-fed *Lepr-KO* rats, in the presence of metformin, daunorubicin decreased CCO expression, with minimal influence on p-AMPK. This suggested that CCO acts downstream of activated AMPK. The CCO overexpression may reflect a hyperfunctional state of mitochondria, and may be the result of Lepr defective under the direct influence of metformin. Thus, long-term stimulation with metformin caused the overexpression of glucose metabolism-related genes, and promoted the hyper-activation of the mitochondrial oxidative respiratory chain in hepatocytes with Lepr defective. This led to over-consumption of cellular energy, which aggravated hepatocyte degeneration followed by pyroptosis. This is consistent with previous reports demonstrating the roles of AMPK phosphorylation in promoting catabolic pathways by activating CCO [48, 49].

In the presence of metformin, we noticed elevated production of IL-1β and IL-18, as well as Caspase-5, Caspase-11, and cleaved Caspase-1 and Caspase-3. Additionally, we noticed GSDME-N in the HFD-fed *Lepr*-defective hepatocytes, the expression levels of which decreased markedly at 96 h post-transfection of *Lepr*. However, the expression of both cleaved Caspase-8 and Caspase-9 were minimally affected after *Lepr* overexpression. This suggested that pyroptosis, and not apoptosis, had occurred. Since the pyroptotic markers’ levels could be restored, and hyperactive glucose metabolism could be controlled by the re-expression of Lepr in *Lepr*-deficient hepatocytes, we presumed that pyroptosis is the main contributing factor behind metformin’s negative effect on *Lepr*-defective hepatocytes. Pyroptosis is a serious inflammatory type of programmed cell death. After activation of inflammasome complex, Caspase-1 is cleaved and activated to induce production of the IL-1β, IL-18 and the pyroptotic effectors GSDMD and GSDME [50]. GSDME contains an amino-terminal pore-forming domain that triggers pyroptosis [51–53]. It has been reported that GSDME cleavage at Asp270 is induced by apoptosis-associated Caspase-3, converting apoptotic signals to pyroptosis [54]. This has been verified by our observations in view of the higher expression of cleaved Caspase-3 in metformin-treated *Lepr*-deficient hepatocytes. A previous study has shown a correlation between increased levels of Gasdermin family members and improvement in the liver function, in both NAFLD patients and db/db mouse models [55]. Also, pyroptosis inhibition can reduce liver inflammation and improve the overall NAFLD activity score [56]. It has been shown that metformin-induced pyroptosis is activated through AMPK/silent information regulator 1 (SIRT1) pathway in tumor cells, where increased NFκB p65 stimulates Bax and cytochrome C release. This triggers Caspase-3 cleavage [57]. Meanwhile, our study offers a new perspective, speculating that through overactivation of AMPK/CCO pathway, long-term metformin treatment could promote pyroptosis in *Lepr*-defective hepatocytes, leading to severe hepatic damage in *Lepr*-defective individuals. Our results suggest that metformin’s capacity in promoting glucose metabolism was amplified in the *Lepr*-defective milieu. Moreover, the hyper-activated glucose metabolism may elicit over-consumption of cell energetic reservoir, and drive severe inflammation followed by pyroptosis. Restoring Lepr expression in *Lepr*-defective hepatocytes could rescue cells from AMPK/CCO axis overactivation and pytoptosis.

Telomeres are specific structures found at the end of chromosomes in eukaryotes, and telomerase is a ribonucleoprotein that synthesizes and directs the telomeric repeats onto the 3′ end of existing telomeres using its RNA component as a template, where it compensates for telomere shortening during DNA replication, and thus stabilizes telomere length [58]. Therefore, telomere length may function as a “barometer” reflecting the number of cell divisions, and finally signaling replicative senescence or programmed cell death when a critical telomere length is reached. We measured the telomerase activity and telomere length in hepatocytes isolated from HFD-fed *Lepr*-KO rats and their corresponding controls, in the presence of metformin. Our findings showed that long-term metformin treatment resulted in lower telomerase activity and shorter telomere length in *Lepr*-deficient hepatocytes. It was possible to reverse this, at least partially, by *Lepr* re-expression in *Lepr*-deficient hepatocytes. Thus, we assumed that lower telomerase activity and shorter telomere length might also act as pivotal indicators for hepatocyte pyroptosis induced by metformin in the *Lepr*-deficient niche.

Leptin is normally produced by adipose tissue, and it has been correlated with cellular metabolism [59]. In order to maintain energy balance in the liver, leptin needs to interact with AMPK, which regulates the liver’s metabolic fluxes [60]. Many studies have shown that NAFLD can be ameliorated by activating AMPK-related pathways [61, 62], and aggravated when those pathways are suppressed [63]. In the *leptin*-deficient ob/ob mouse models, there was no change in the AMPK levels, however hepatic AMPK phosphorylation was downregulated [41]. Metformin upregulates hepatic p-AMPK expression, which, in turn, rescued the hepatocytes from NAFLD in a model [41]. However, our study reported that *Lepr*-deficient ZDF rats responded to metformin very differently. ZDF rats are congenital *Lepr*-deficient models with severe leptin resistance. This induces hyperlipidemia and fat accumulation in the liver, as shown in our study. AMPK, and particularly p-AMPK, was elevated in ZDF rat livers. We speculated that this was a compensatory response to hyperleptindemia induced by severe leptin resistance. In our study, metformin upregulated phosphorylated AMPK levels and its downstream CCO in HDF-fed ZDF rats. This resulted in glucose metabolism hyperactivity and pyroptosis which then accelerated liver damage. We had similar observations in our *Lepr*-KO rat models established via the CRISPR/Cas9 system. Although long-term metformin treatment did slightly upregulate the Lepr expression, it could not override leptin resistance in the ZDF rat livers. Our study’s findings also showed that leptin supplement may mitigate metformin’s detrimental effect on *Lepr*-deficient rat-derived hepatocytes. In the presence of metformin, 10 μM leptin could slightly increase Lepr expression while downregulating p-AMPK and CCO levels; we found a negative correlation between Lepr expression and CCO expression (*r* = −0.912, *p* < 0.05). On this basis, we speculated that administration in combination with leptin to *Lepr*-defectiveive individuals with NAFLD might mitigate the risk in metformin treatment. Thus, further efforts should be directed towards that end by means of stratified clinical trials with regard to combinatorial leptin administration.

We also reported a more dynamic model of glucose regulation, using *Lepr*-deficient hepatocytes exposed to metformin. Single-cell RNA sequencing showed that the key players in glucose metabolism had undergone significant elevation in their transcription levels. This suggested that the glucose metabolism had been hyper-activated before cellular pyroptosis. The progress of glucose metabolism was coupled with increased expression of pyroptotic factors. During this process, apoptotic markers including *Caspase-8* and *Caspase-9* remained stable. This indicated that the metformin-treated *Lepr*-deficient hepatocytes would eventually undergo pyroptosis. In summary, our results indicated that the efficacy of metformin treatment on NAFLD individuals with T2D is highly dependent on leptin sensitivity, or the binding between leptin and its receptor. HFD reduces the expression of leptin receptors in animals, causing mild leptin resistance. Metformin has been shown to increase hepatic *Lepr* expression, thereby decreasing steatosis in HFD-fed mice [30]. However, metformin did not override leptin resistance in the *Lepr*-deficient rats in our study, since these rats carried a genetic defective in Lepr. Even the up regulation of these defectiveive receptors would have been unlikely to improve leptin binding.

Our study has several limitations. Firstly, we used hepatocytes isolated from the livers of ZDF rats and *Lepr*-KO rats (but not from liver-specific *Lepr*-KO rats) to detect hepatic *Lepr* defective’s effect on metformin-treated NAFLD. This does not entirely rule out the possibility of neurohumoral regulation by CNS Lepr signaling. Although individual cell experiments provide substantial evidence that metformin is harmful in NAFLD treatment when there is a Lepr defective in the hepatocytes, this is still in need of verification in liver-specific *Lepr*-KO animals before applying it to clinical settings. Secondly, after AMPK inhibitor treatment, the elevated p-AMPK expression in hepatocytes from metformin-treated *Lepr*-deficient rats returned to its original state. During this process, levels of CCO and cleaved Caspase-3 dropped, yet they remained higher than those in control Lepr-deficient rats. This suggests that metformin may promote pyroptosis through mechanisms other than AMPK activation. This could be addressed in future studies. In addition, the information that we acquired from animal models might not be entirely applicable to human patients. However, our results have important implications for clinical research. For example, we showed that the genetic defective in the *Lepr* was responsible for the ineffectiveness or detrimental effects of the long-term metformin treatment on NAFLD in rats. In T2D patients, Lys656Asn polymorphism of the *Lepr* gene led to leptin resistance [64]. This polymorphism has also been associated with insulin resistance and abnormal glucose levels in patients with NAFLD [64]. It has been reported that patients with the mutant allele have a different response to weight loss caused by the high monounsaturated fat hypocaloric diet, with little improvement in either leptin resistance or insulin resistance [65]. Therefore, leptin resistance or *Lepr* gene polymorphism should be considered in future randomized control trials of long-term metformin treatment on NAFLD in patients preconditioned with T2D. By conducting a stratified clinical trial, we may be able to distinguish patients who may benefit from metformin treatment from those who should avoid it. In summary, our results imply that long-term metformin treatment has no preventive or therapeutic effect on NAFLD in *Lepr*-deficient rats, but it does increase liver damage. Metformin administration via AMPK phosphorylation and CCO activation would require integral Lepr or preserved leptin sensitivity for effective NAFLD treatment in patients with T2D.

## Conclusion

Hepatic *Lepr* defect independently increased liver damage in the process of long-term metformin treatment via overactivation of the AMPK-CCO axis and consequent pyroptosis. Additionally, the functional Lepr was necessary for the favorable effects of metformin administration.

## Supplementary information

aj-checklist

Author contacts

Fig 2- Single Original Western blotting images

Fig 4- Single Original Western blotting images

Fig 5-Single Original Western blotting images

Fig 7-Single Original Western blotting images

Fig 8-Single Original Western blotting images

Supplementary material 1-Fig 2A

Supplementary material 2-Fig 2B

Supplementary material 3-Fig 4A

Supplementary material 4-Fig 5A

Supplementary material 5-Fig 5C

Supplementary material 6-Fig 7-01

Supplementary material 7-Fig 7-02

Supplementary material 8-Fig 8-01

Supplementary material 9-Fig 8-02

Supplementary material
