# Supplementary material for: Metformin induces pyroptosis in leptin receptor-defective hepatocytes via overactivation of the AMPK axis
Source: Cell Death Dis. 2023 Feb 3;14(2):82. doi: 10.1038/s41419-023-05623-4 (PMC9898507; doi:10.1038/s41419-023-05623-4)
Supplement: Supplementary file 2 — Author contacts [file 41419_2023_5623_MOESM2_ESM.doc]

| Author | Fax | Department | E-mail | Corresponding |
| --- | --- | --- | --- | --- |
| Bingli Liu | 86-21-31342617 | Department of Orthopedics, Shanghai Pudong New Area People's Hospital, 490 Chuanhuan South Road, Pudong New Area, Shanghai, 201299, China. | doctorblliu@126.com | No |
| Jingyuan Xu | 86-21-68035001 | Department of Gastroenterology, Shanghai Pudong Hospital, Fudan University Pudong Medical Center, Shanghai 201399, China | 498520087@qq.com | No |
| Linyao Lu | 86-21-68036506 | Center for Medical Research and Innovation, Shanghai Pudong Hospital, Fudan University Pudong Medical Center, Shanghai 201399, China | 1098453467@qq.com | No |
| Lili Gao | 86-21-68036506 | Center for Medical Research and Innovation, Shanghai Pudong Hospital, Fudan University Pudong Medical Center, Shanghai 201399, China | upsetmonkey@163.com | No |
| Shengjuan Zhu | 86-21-68036506 | Center for Medical Research and Innovation, Shanghai Pudong Hospital, Fudan University Pudong Medical Center, Shanghai 201399, China | mrszhushengjuan@163.com | No |
| Yi Sui | 86-20-87332200 | Department of Nutrition, The First Affiliated Hospital of Sun Yat-sen University, Guangzhou 510080, Guangdong Province, China | suiyisy2005@sina.com | No |
| Ting Cao | 86-21-68036506 | Center for Medical Research and Innovation, Shanghai Pudong Hospital, Fudan University Pudong Medical Center, Shanghai 201399, China | scientific2fudan@163.com | No |
| Tao Yang | 86-21-68036506 | Center for Medical Research and Innovation, Shanghai Pudong Hospital, Fudan University Pudong Medical Center, Shanghai 201399, China | yangt@fudan.edu.cn | Yes |
