# Supplementary figures and images for: Metformin induces pyroptosis in leptin receptor-defective hepatocytes via overactivation of the AMPK axis (part 1 of 2)
Source: Cell Death Dis. 2023 Feb 3;14(2):82. doi: 10.1038/s41419-023-05623-4 (PMC9898507; doi:10.1038/s41419-023-05623-4)

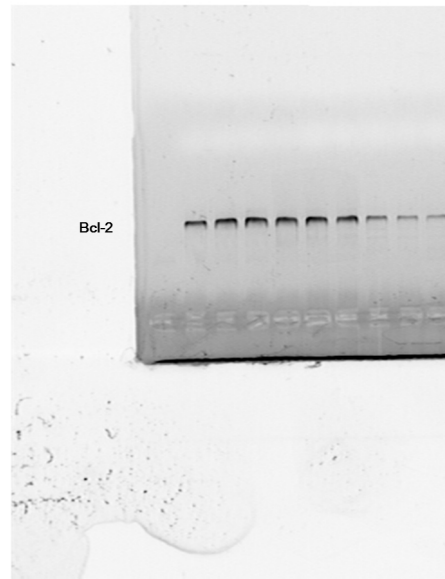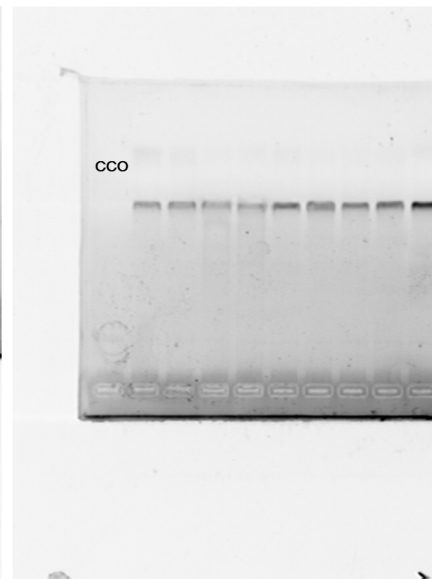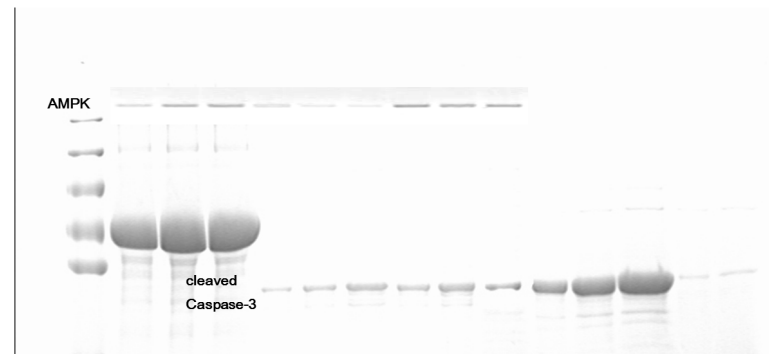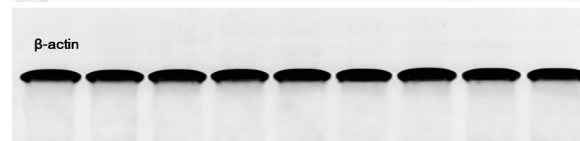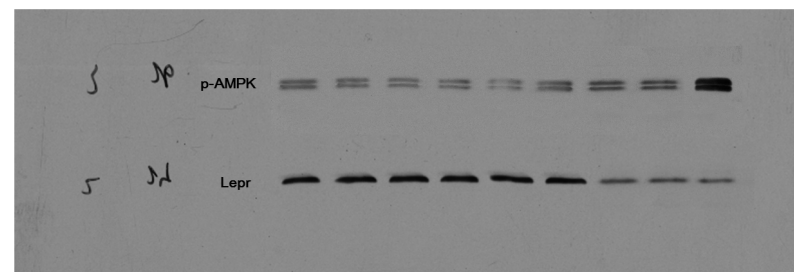

Supplement: Supplementary file 3 — Fig 2- Single Original Western blotting images [file 41419_2023_5623_MOESM3_ESM.zip › Fig 2- Single Original Western blotting images/Fig 2A (Original film).pdf]

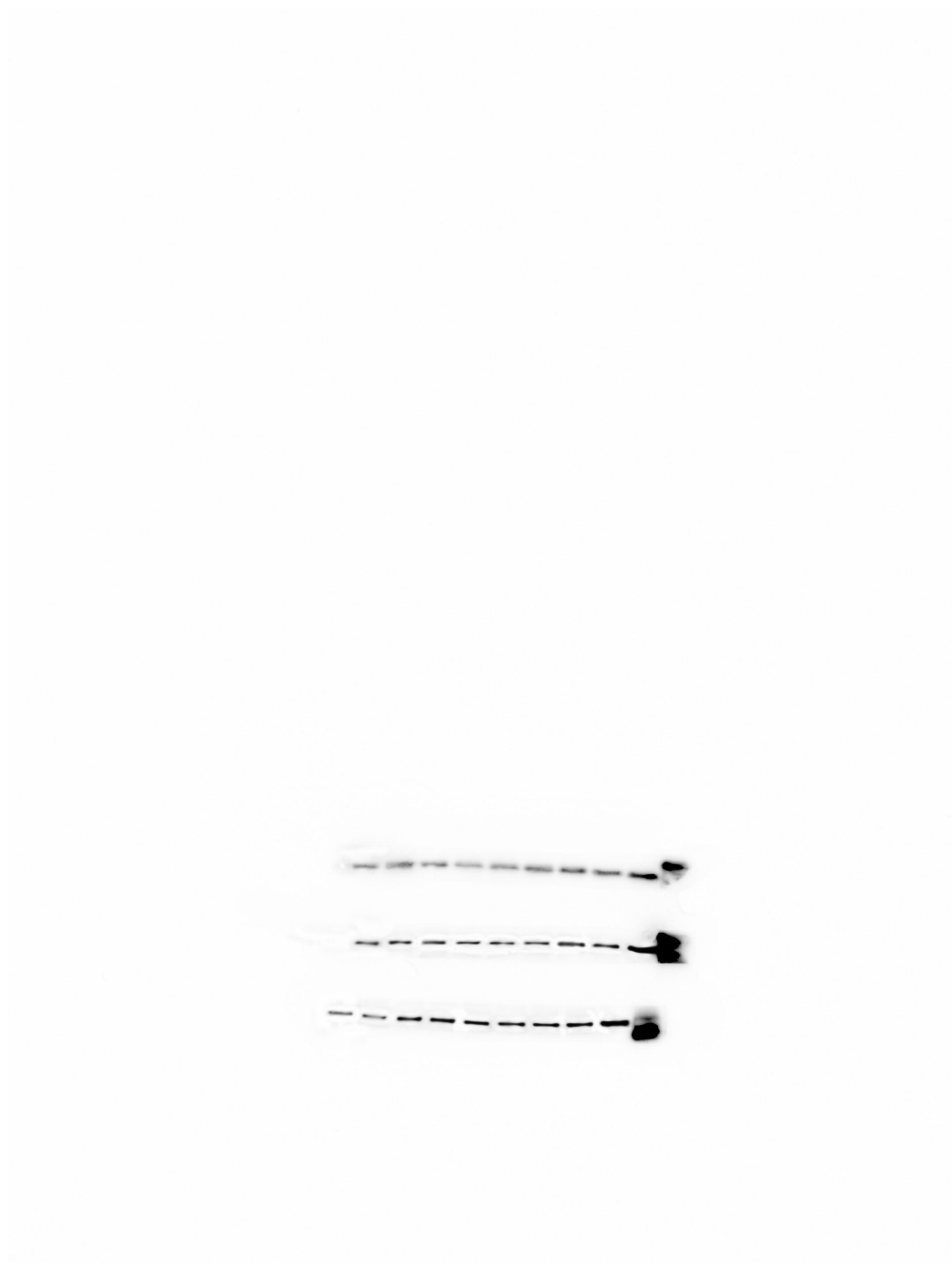

Supplement: Supplementary file 3 — Fig 2- Single Original Western blotting images [file 41419_2023_5623_MOESM3_ESM.zip › Fig 2- Single Original Western blotting images/Fig 2A/01 02 03 Cleaved cas3.pdf]

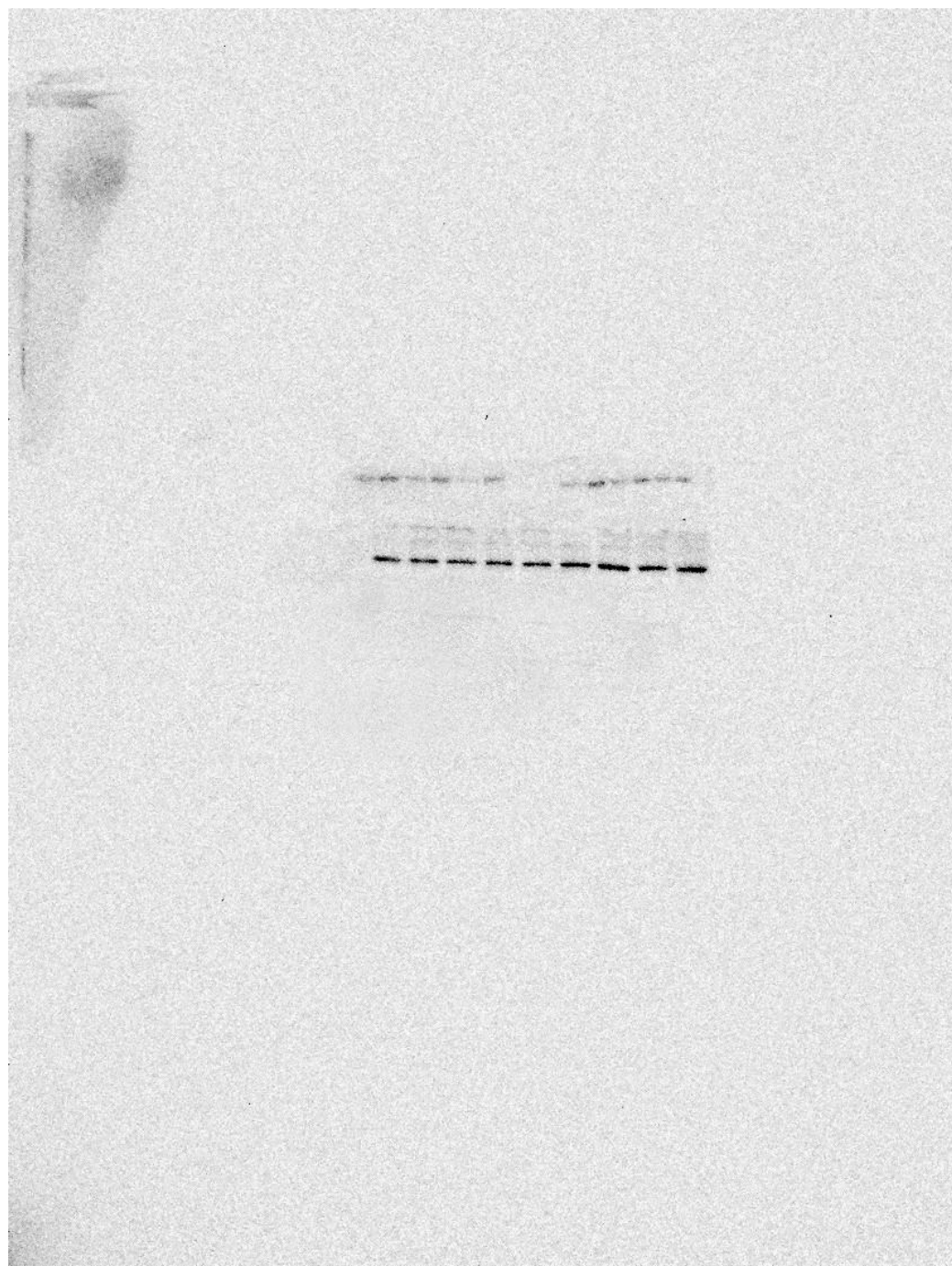

Supplement: Supplementary file 3 — Fig 2- Single Original Western blotting images [file 41419_2023_5623_MOESM3_ESM.zip › Fig 2- Single Original Western blotting images/Fig 2A/01 AMPK.pdf]

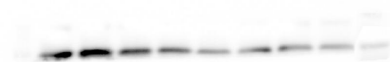

Supplement: Supplementary file 3 — Fig 2- Single Original Western blotting images [file 41419_2023_5623_MOESM3_ESM.zip › Fig 2- Single Original Western blotting images/Fig 2A/01 Bcl2.pdf]

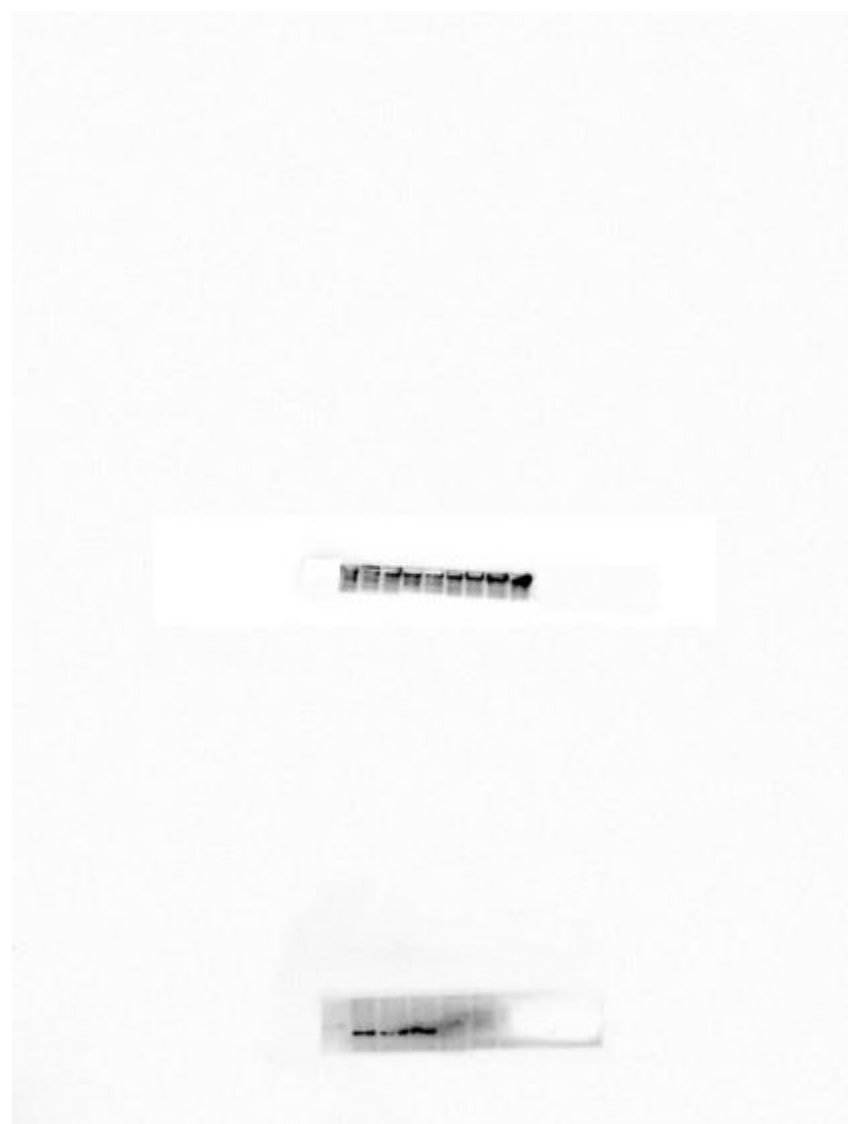

Supplement: Supplementary file 3 — Fig 2- Single Original Western blotting images [file 41419_2023_5623_MOESM3_ESM.zip › Fig 2- Single Original Western blotting images/Fig 2A/01 CCO.pdf]

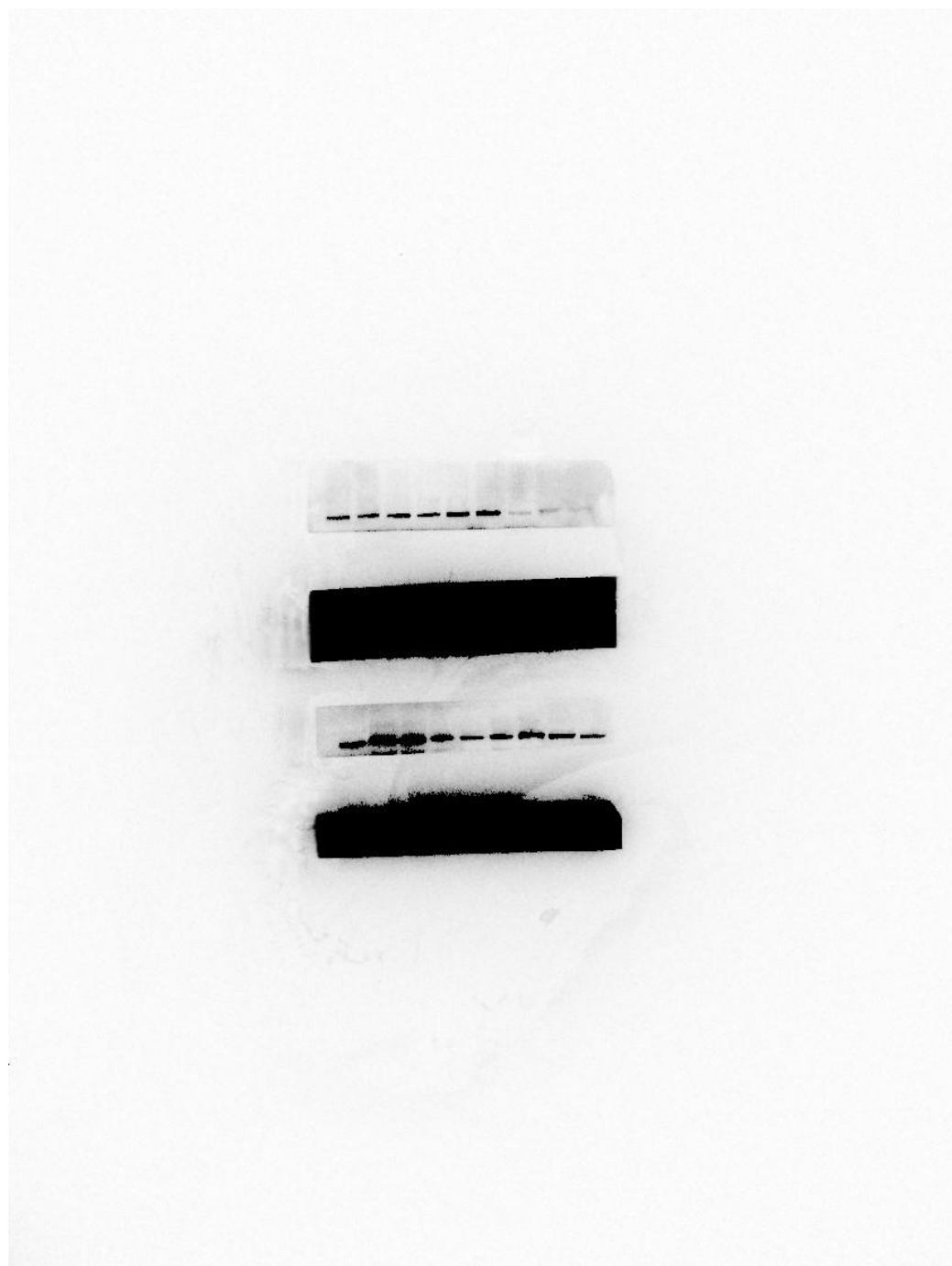

Supplement: Supplementary file 3 — Fig 2- Single Original Western blotting images [file 41419_2023_5623_MOESM3_ESM.zip › Fig 2- Single Original Western blotting images/Fig 2A/01 Lepr & beta-actin.pdf]

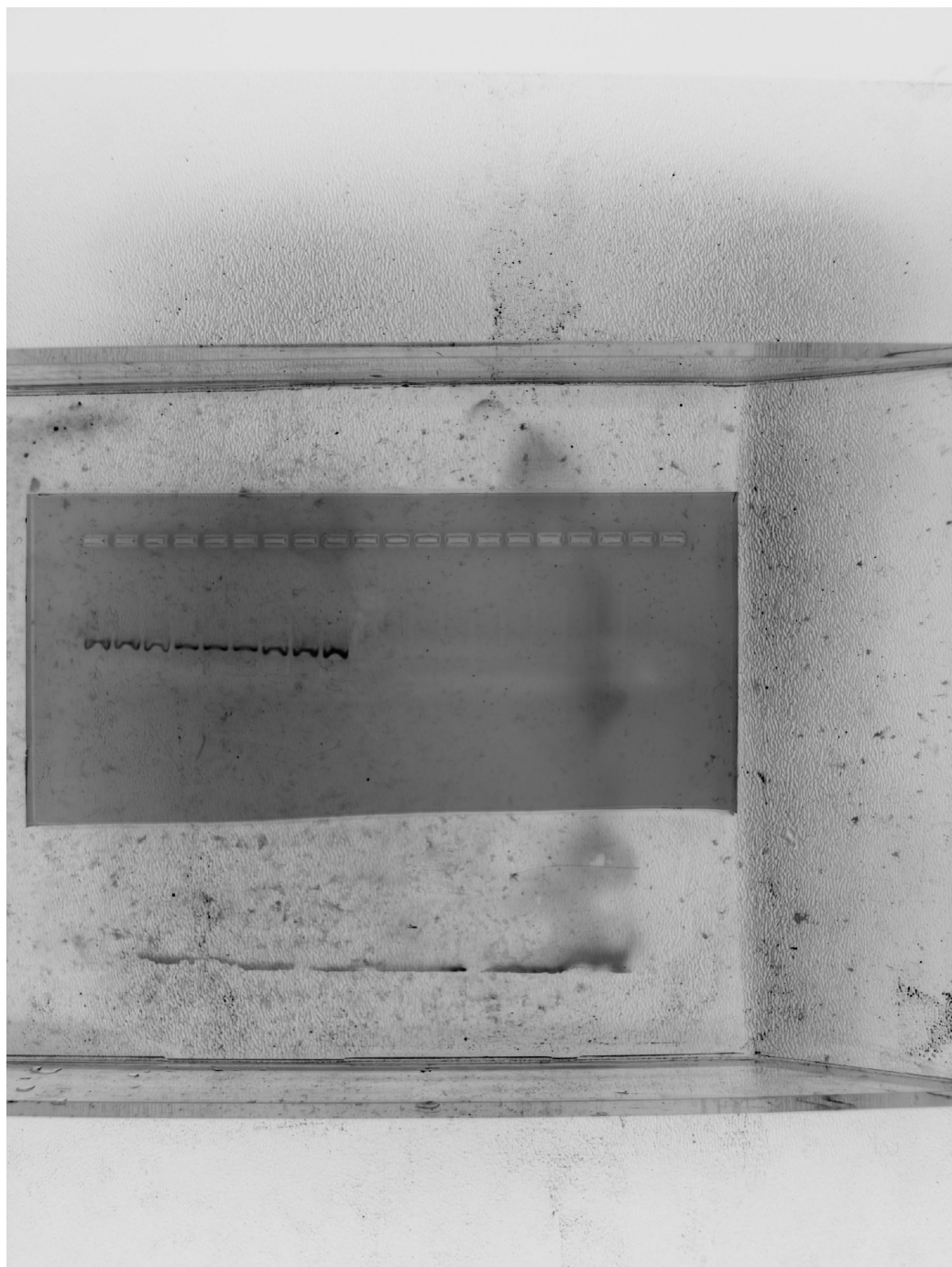

Supplement: Supplementary file 3 — Fig 2- Single Original Western blotting images [file 41419_2023_5623_MOESM3_ESM.zip › Fig 2- Single Original Western blotting images/Fig 2A/01 pAMPK.pdf]

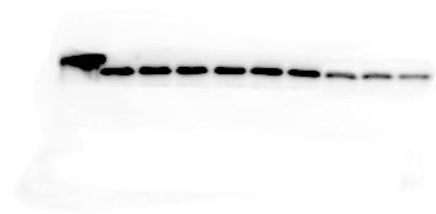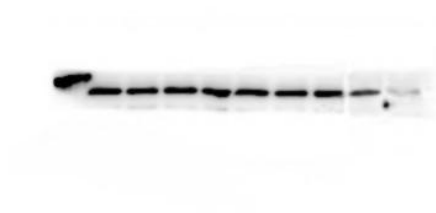

Supplement: Supplementary file 3 — Fig 2- Single Original Western blotting images [file 41419_2023_5623_MOESM3_ESM.zip › Fig 2- Single Original Western blotting images/Fig 2A/02 03 Bcl2.pdf]

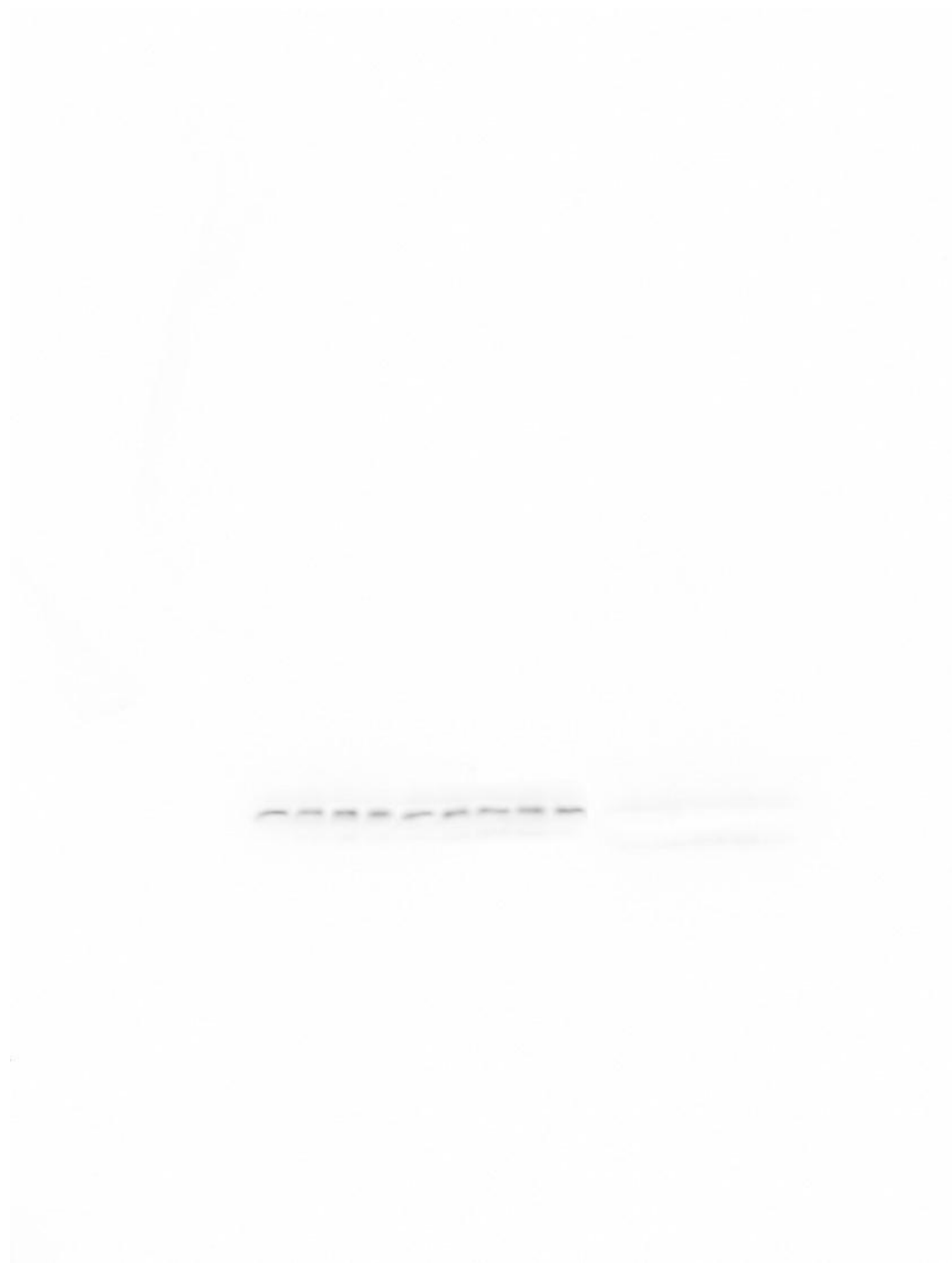

Supplement: Supplementary file 3 — Fig 2- Single Original Western blotting images [file 41419_2023_5623_MOESM3_ESM.zip › Fig 2- Single Original Western blotting images/Fig 2A/02 AMPK.pdf]

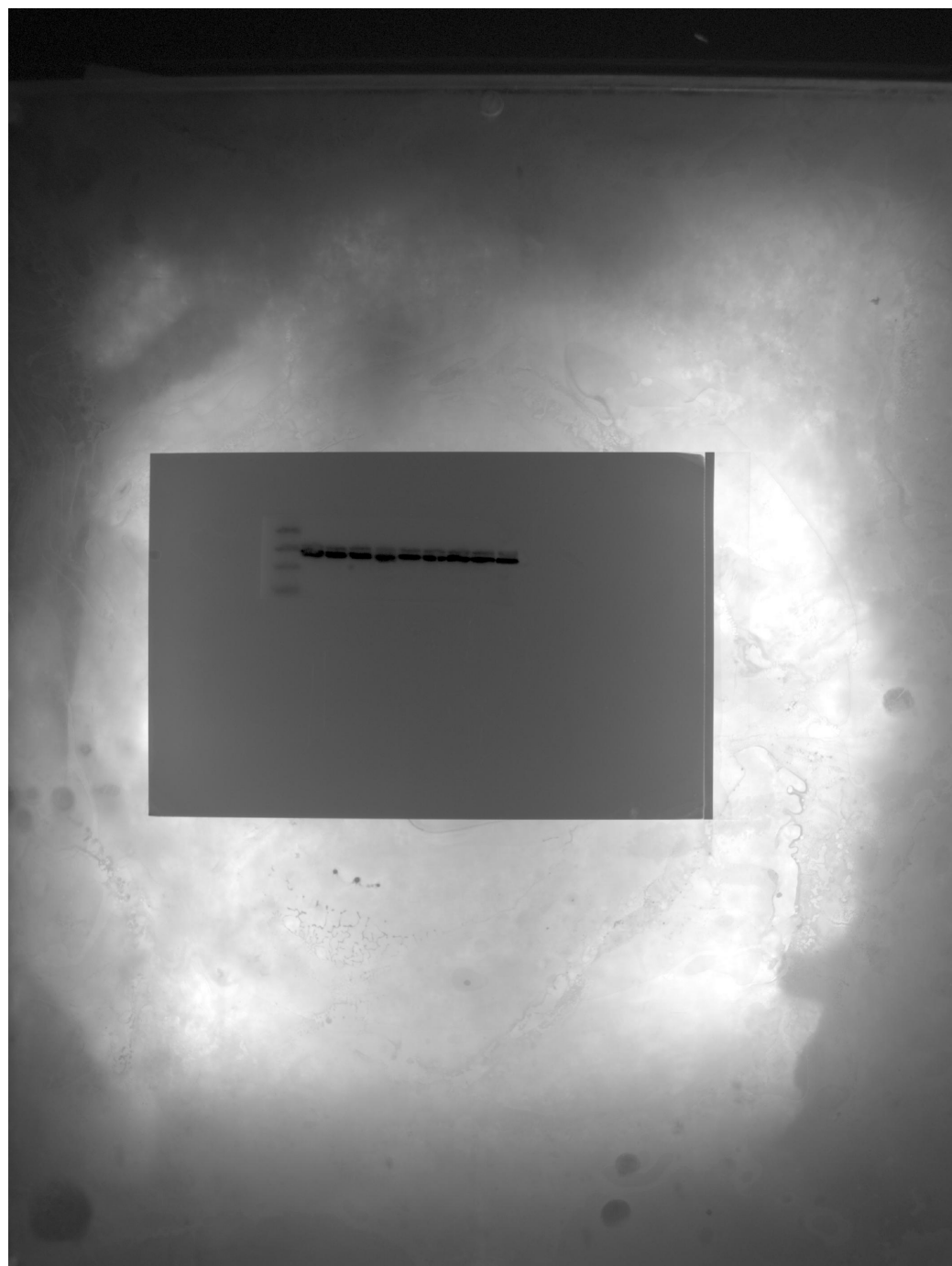

Supplement: Supplementary file 3 — Fig 2- Single Original Western blotting images [file 41419_2023_5623_MOESM3_ESM.zip › Fig 2- Single Original Western blotting images/Fig 2A/02 beta-actin.pdf]

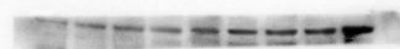

Supplement: Supplementary file 3 — Fig 2- Single Original Western blotting images [file 41419_2023_5623_MOESM3_ESM.zip › Fig 2- Single Original Western blotting images/Fig 2A/02 CCO.pdf]

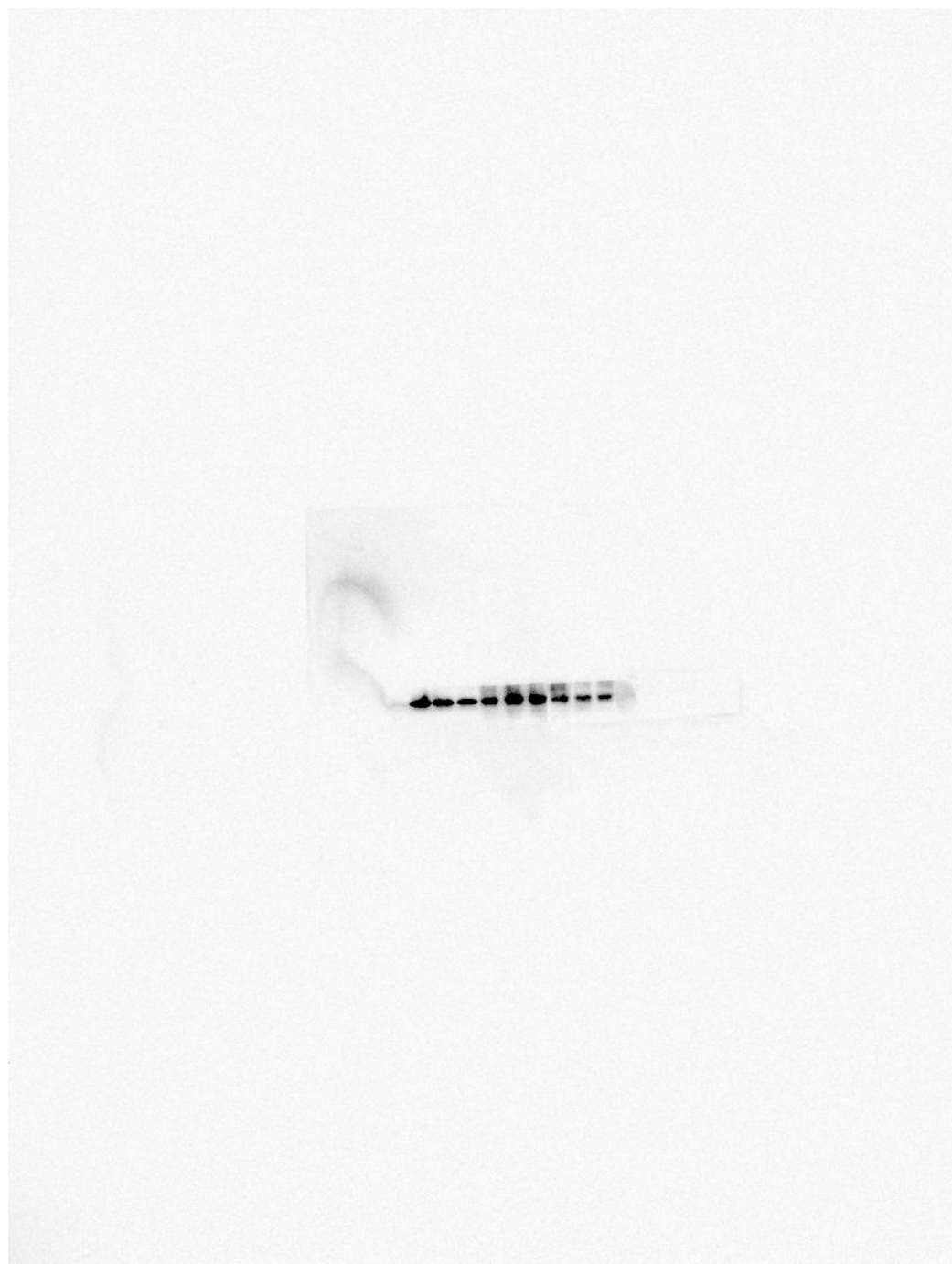

Supplement: Supplementary file 3 — Fig 2- Single Original Western blotting images [file 41419_2023_5623_MOESM3_ESM.zip › Fig 2- Single Original Western blotting images/Fig 2A/02 Lepr.pdf]

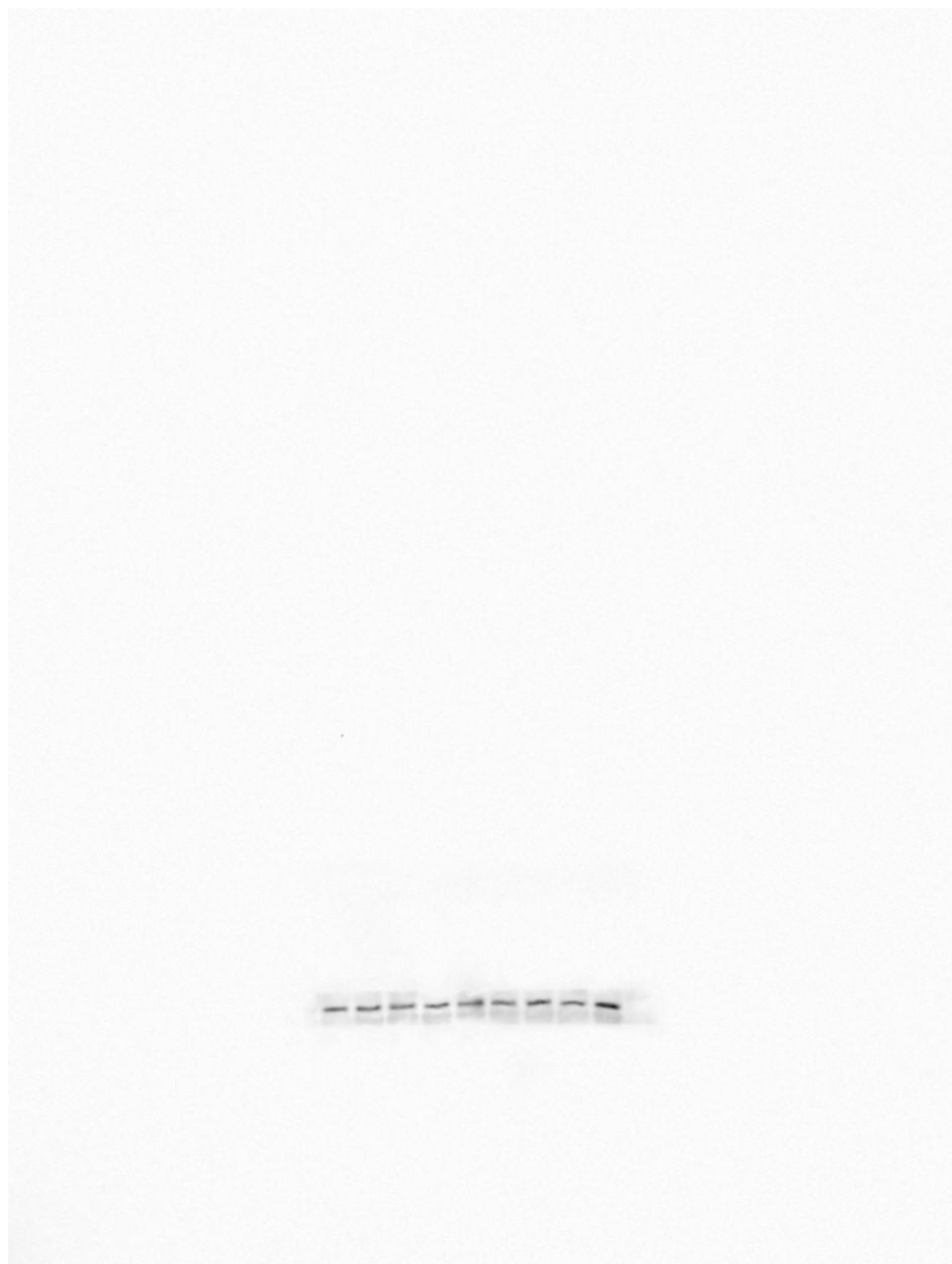

Supplement: Supplementary file 3 — Fig 2- Single Original Western blotting images [file 41419_2023_5623_MOESM3_ESM.zip › Fig 2- Single Original Western blotting images/Fig 2A/02 pAMPK.pdf]

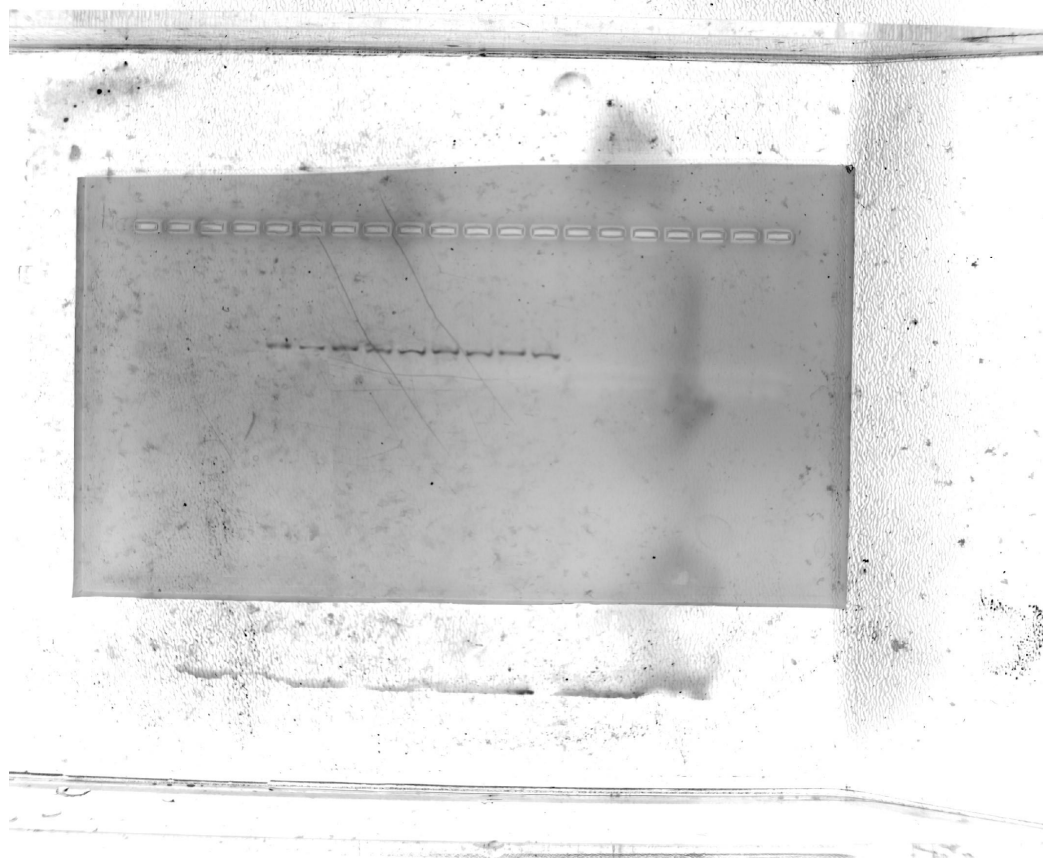

Supplement: Supplementary file 3 — Fig 2- Single Original Western blotting images [file 41419_2023_5623_MOESM3_ESM.zip › Fig 2- Single Original Western blotting images/Fig 2A/03 AMPK.pdf]

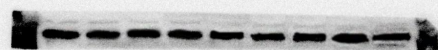

Supplement: Supplementary file 3 — Fig 2- Single Original Western blotting images [file 41419_2023_5623_MOESM3_ESM.zip › Fig 2- Single Original Western blotting images/Fig 2A/03 beta-actin.pdf]

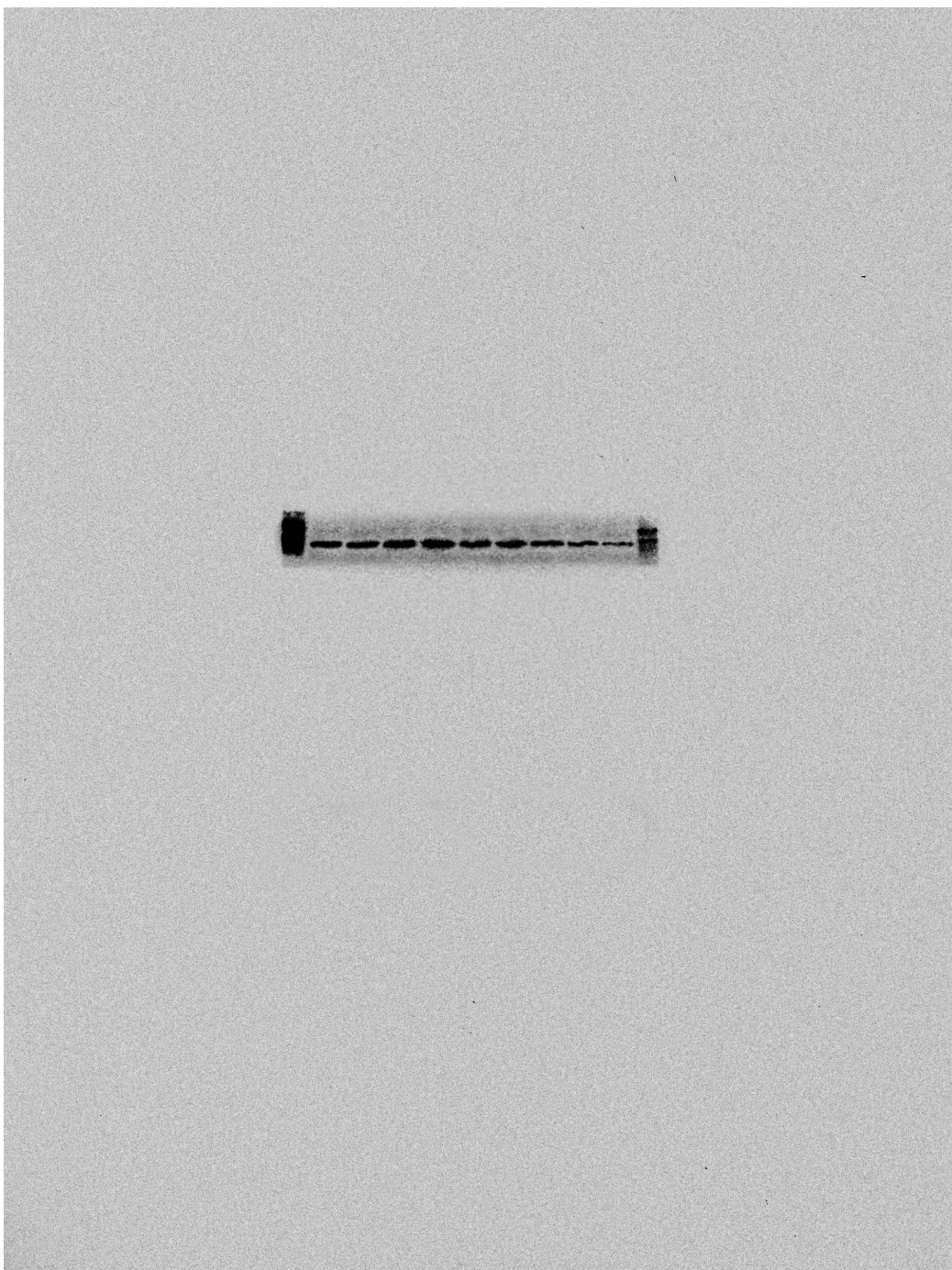

Supplement: Supplementary file 3 — Fig 2- Single Original Western blotting images [file 41419_2023_5623_MOESM3_ESM.zip › Fig 2- Single Original Western blotting images/Fig 2A/03 Lepr.pdf]

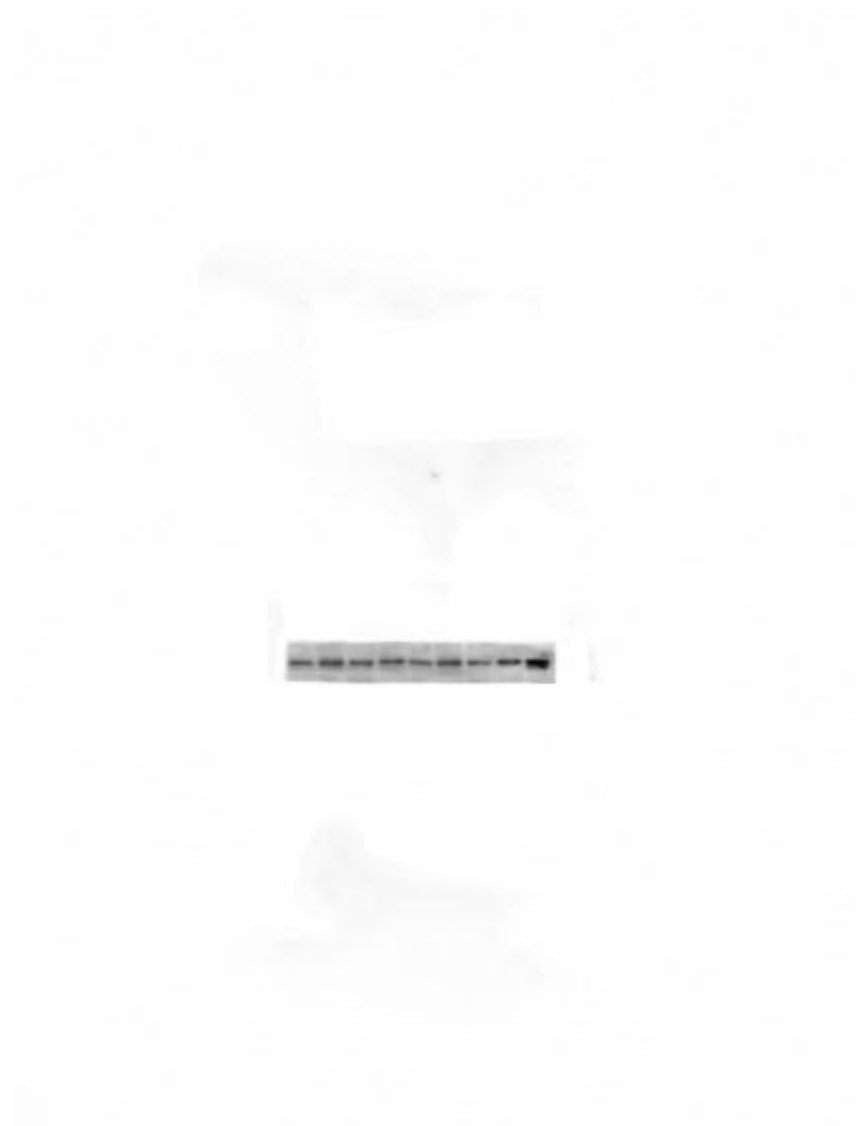

Supplement: Supplementary file 3 — Fig 2- Single Original Western blotting images [file 41419_2023_5623_MOESM3_ESM.zip › Fig 2- Single Original Western blotting images/Fig 2A/03 pAMPK.pdf]

$\beta$ -actin

Bcl-2

CCO

cleaved  
Caspase-3

AMPK

p-AMPK

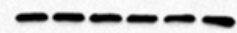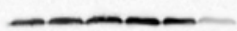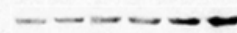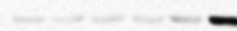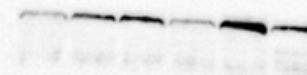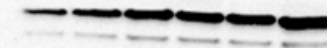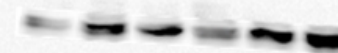

Supplement: Supplementary file 3 — Fig 2- Single Original Western blotting images [file 41419_2023_5623_MOESM3_ESM.zip › Fig 2- Single Original Western blotting images/Fig 2B (Original film).pdf]

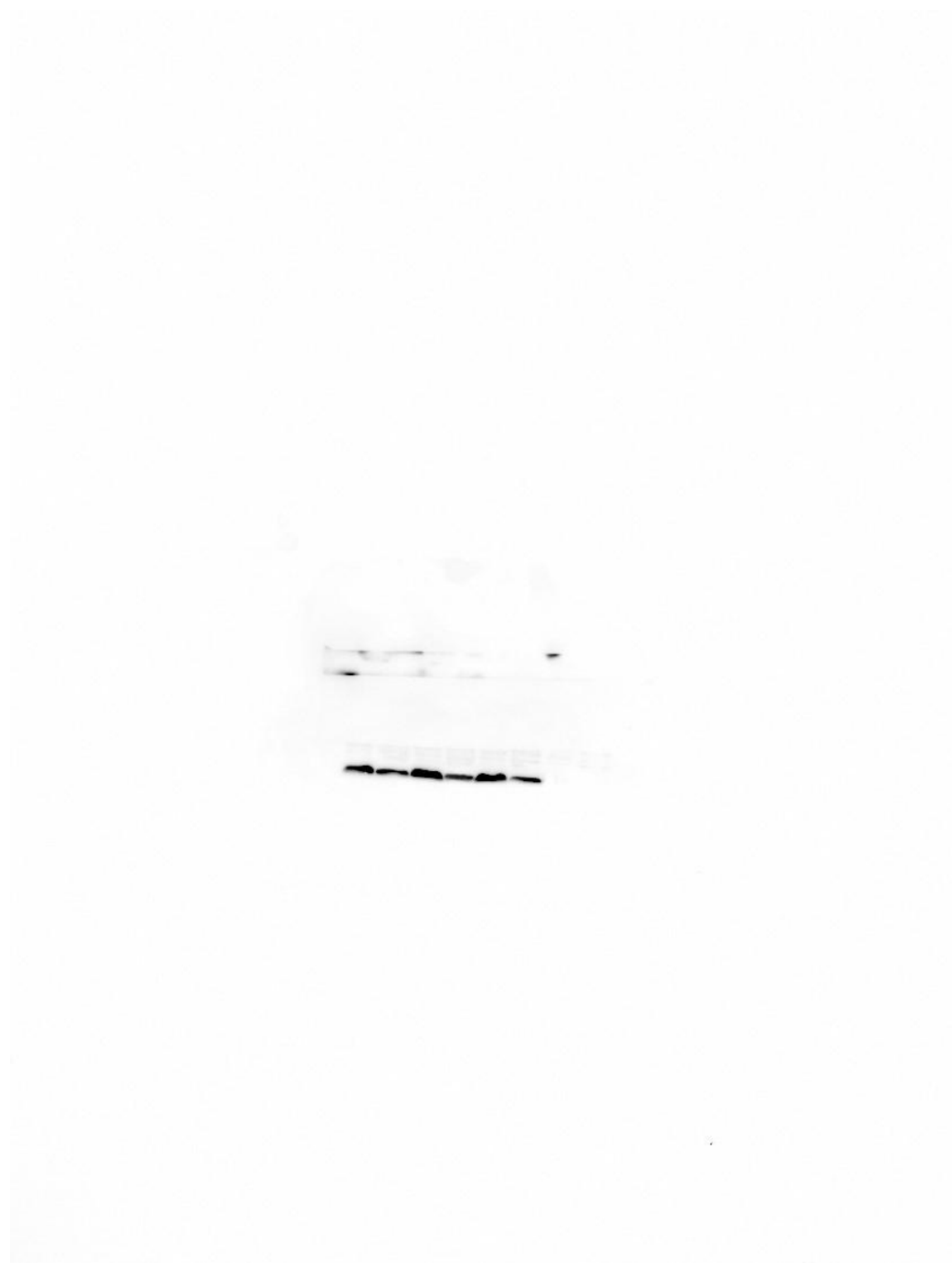

Supplement: Supplementary file 3 — Fig 2- Single Original Western blotting images [file 41419_2023_5623_MOESM3_ESM.zip › Fig 2- Single Original Western blotting images/Fig 2B/01 AMPK (6 lanes).pdf]

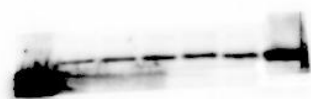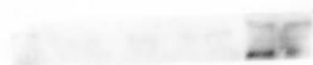

Supplement: Supplementary file 3 — Fig 2- Single Original Western blotting images [file 41419_2023_5623_MOESM3_ESM.zip › Fig 2- Single Original Western blotting images/Fig 2B/01 CCO (6 lanes).pdf]

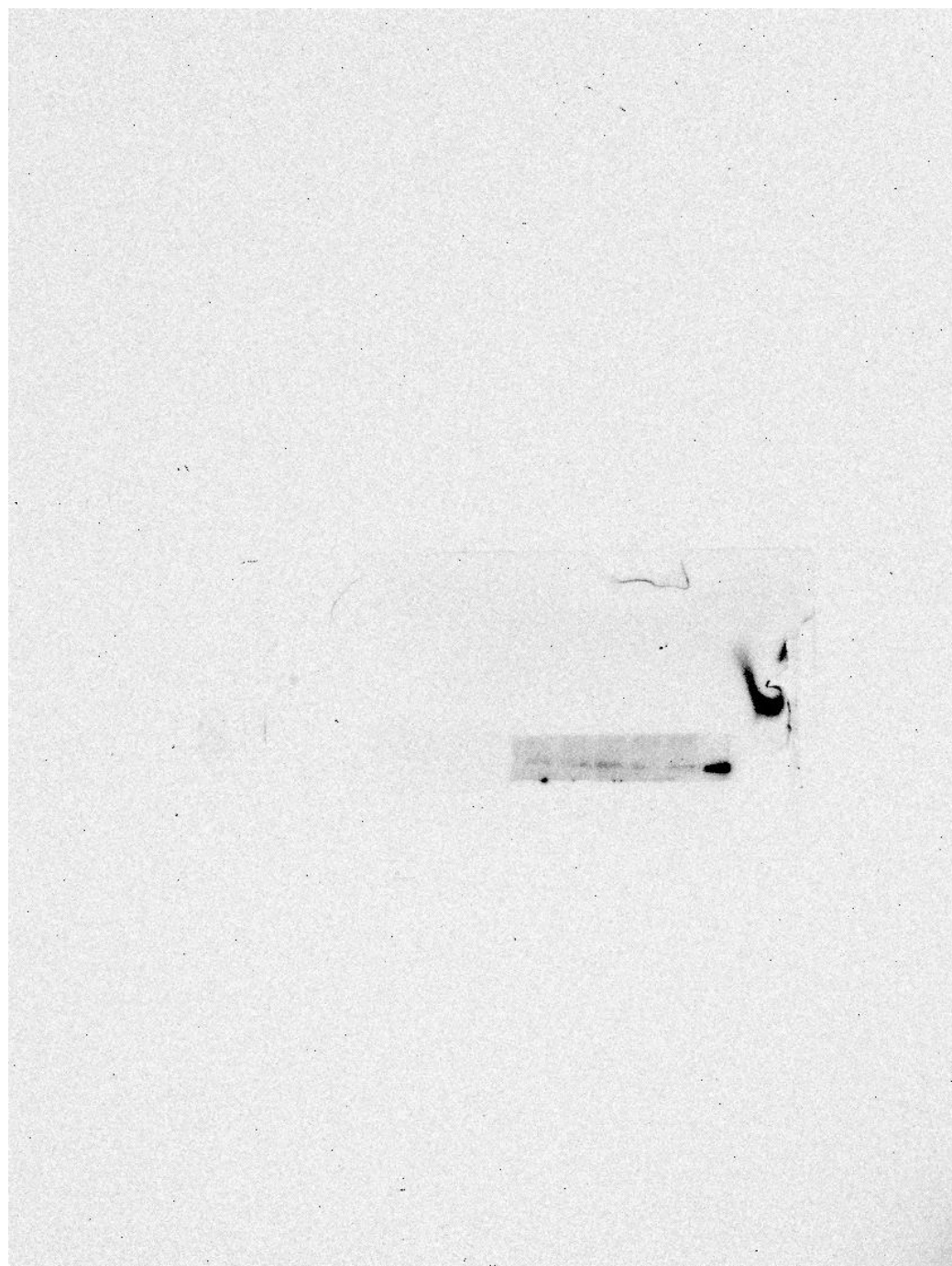

Supplement: Supplementary file 3 — Fig 2- Single Original Western blotting images [file 41419_2023_5623_MOESM3_ESM.zip › Fig 2- Single Original Western blotting images/Fig 2B/01 Cleaved cas3 (6 lanes).pdf]

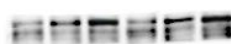

Supplement: Supplementary file 3 — Fig 2- Single Original Western blotting images [file 41419_2023_5623_MOESM3_ESM.zip › Fig 2- Single Original Western blotting images/Fig 2B/01 pAMPK (6 lanes).pdf]

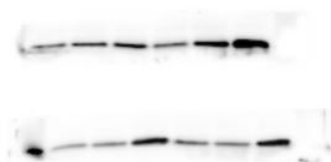

Supplement: Supplementary file 3 — Fig 2- Single Original Western blotting images [file 41419_2023_5623_MOESM3_ESM.zip › Fig 2- Single Original Western blotting images/Fig 2B/02 03 pAMPK (6 lanes).pdf]

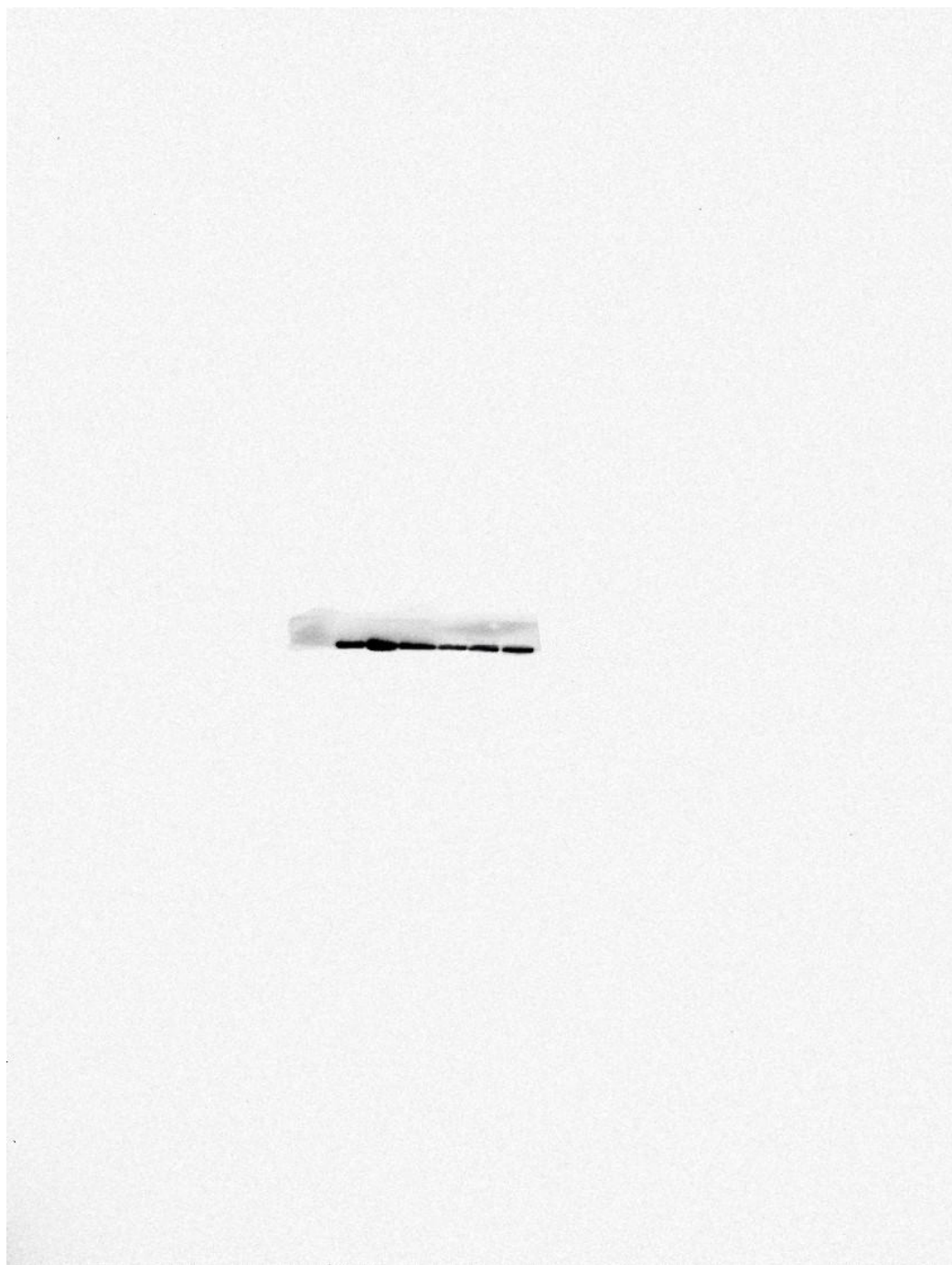

Supplement: Supplementary file 3 — Fig 2- Single Original Western blotting images [file 41419_2023_5623_MOESM3_ESM.zip › Fig 2- Single Original Western blotting images/Fig 2B/02 AMPK (6 lanes).pdf]

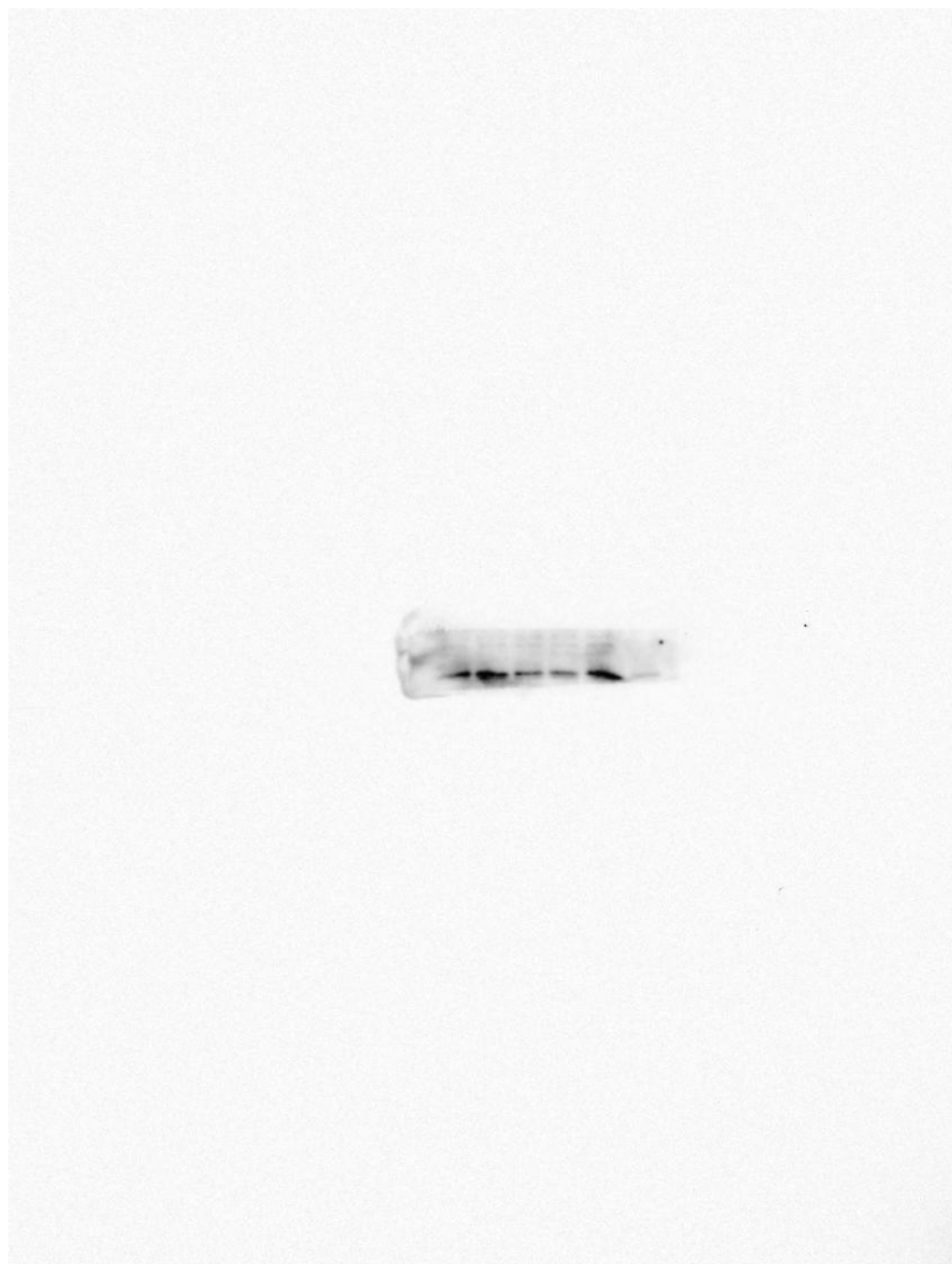

Supplement: Supplementary file 3 — Fig 2- Single Original Western blotting images [file 41419_2023_5623_MOESM3_ESM.zip › Fig 2- Single Original Western blotting images/Fig 2B/02 Bcl2 (6 lanes).pdf]

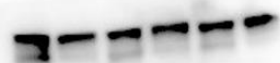

Supplement: Supplementary file 3 — Fig 2- Single Original Western blotting images [file 41419_2023_5623_MOESM3_ESM.zip › Fig 2- Single Original Western blotting images/Fig 2B/02 beta-actin (6 lanes).pdf]

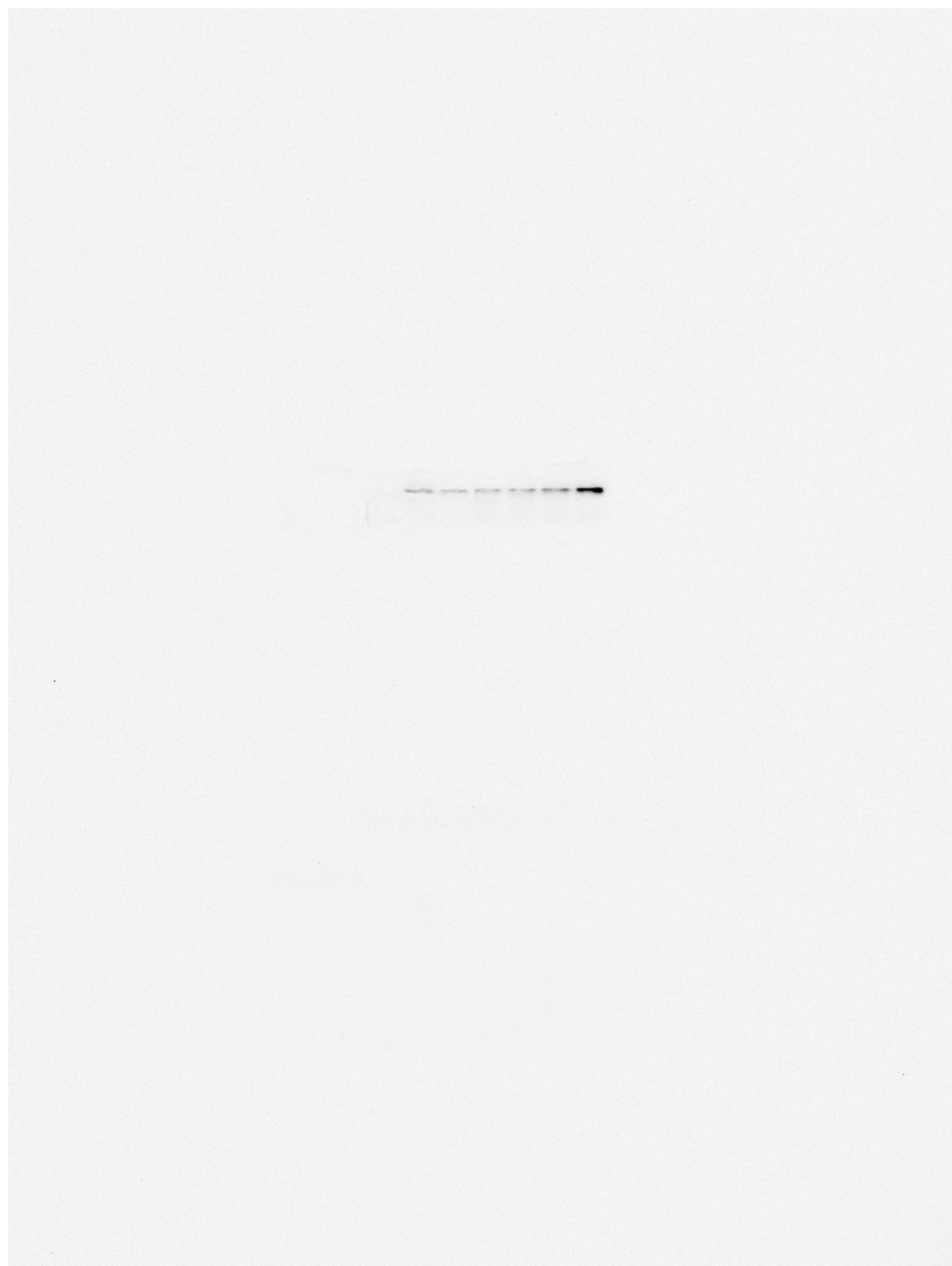

Supplement: Supplementary file 3 — Fig 2- Single Original Western blotting images [file 41419_2023_5623_MOESM3_ESM.zip › Fig 2- Single Original Western blotting images/Fig 2B/02 CCO (6 lanes).pdf]

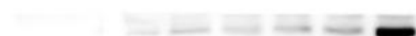

Supplement: Supplementary file 3 — Fig 2- Single Original Western blotting images [file 41419_2023_5623_MOESM3_ESM.zip › Fig 2- Single Original Western blotting images/Fig 2B/02 Cleaved cas3 (6 lanes).pdf]

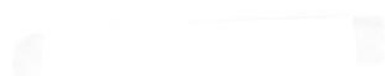

— — — — —

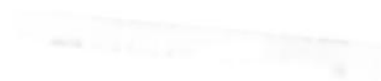

Supplement: Supplementary file 3 — Fig 2- Single Original Western blotting images [file 41419_2023_5623_MOESM3_ESM.zip › Fig 2- Single Original Western blotting images/Fig 2B/03 AMPK (6 lanes).pdf]

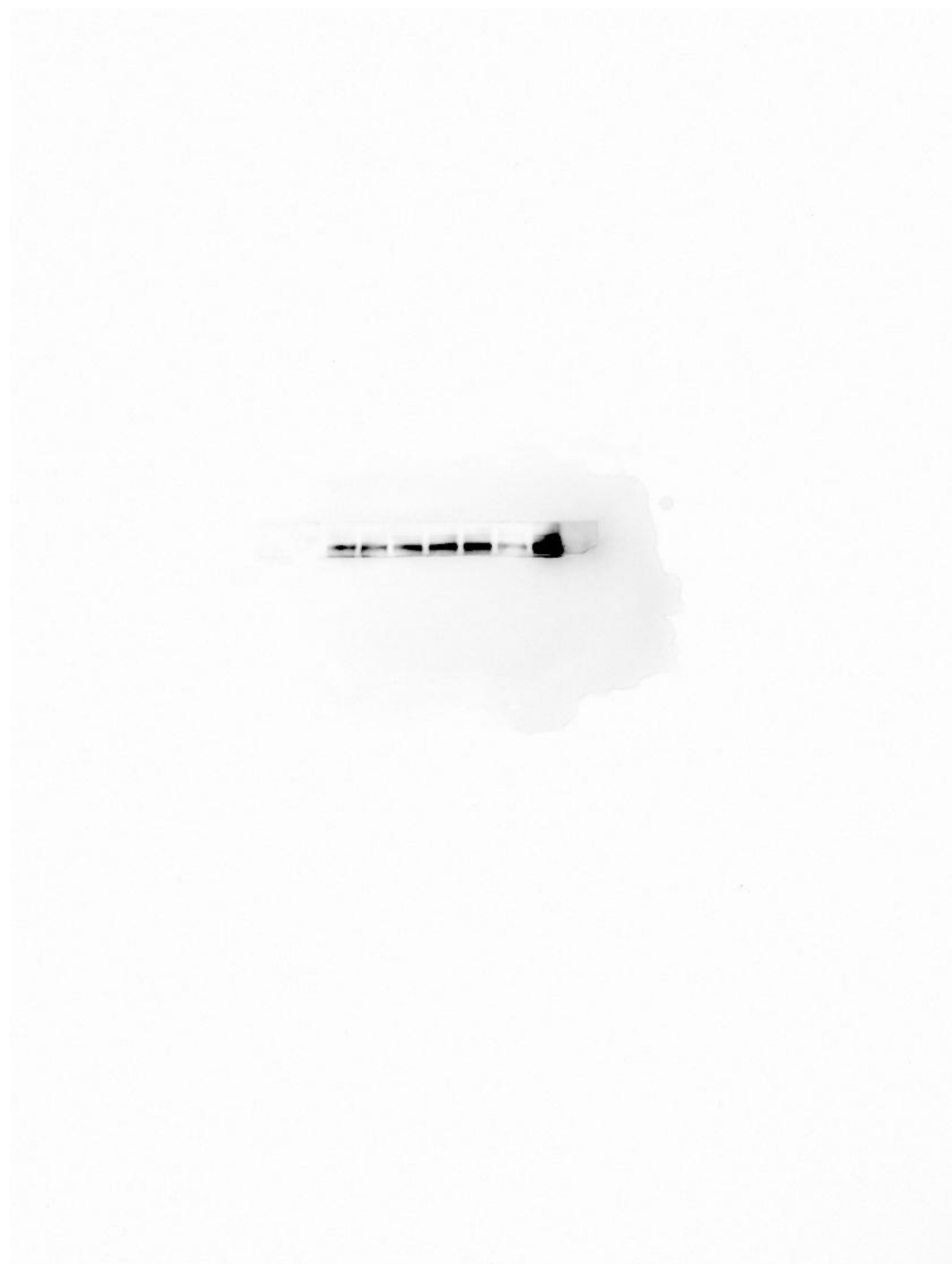

Supplement: Supplementary file 3 — Fig 2- Single Original Western blotting images [file 41419_2023_5623_MOESM3_ESM.zip › Fig 2- Single Original Western blotting images/Fig 2B/03 Bcl2 (6 lanes).pdf]

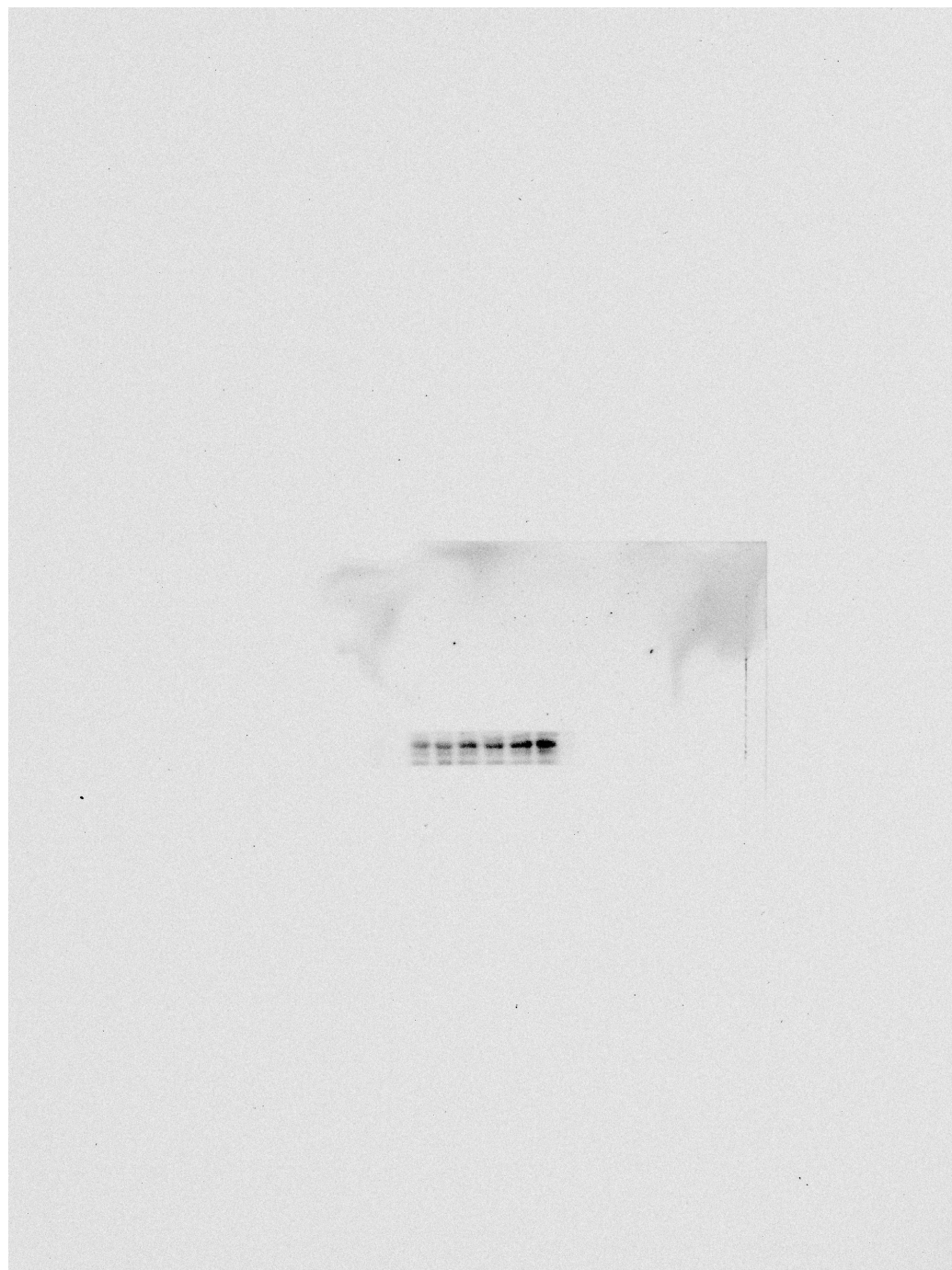

Supplement: Supplementary file 3 — Fig 2- Single Original Western blotting images [file 41419_2023_5623_MOESM3_ESM.zip › Fig 2- Single Original Western blotting images/Fig 2B/03 CCO (6 lanes).pdf]

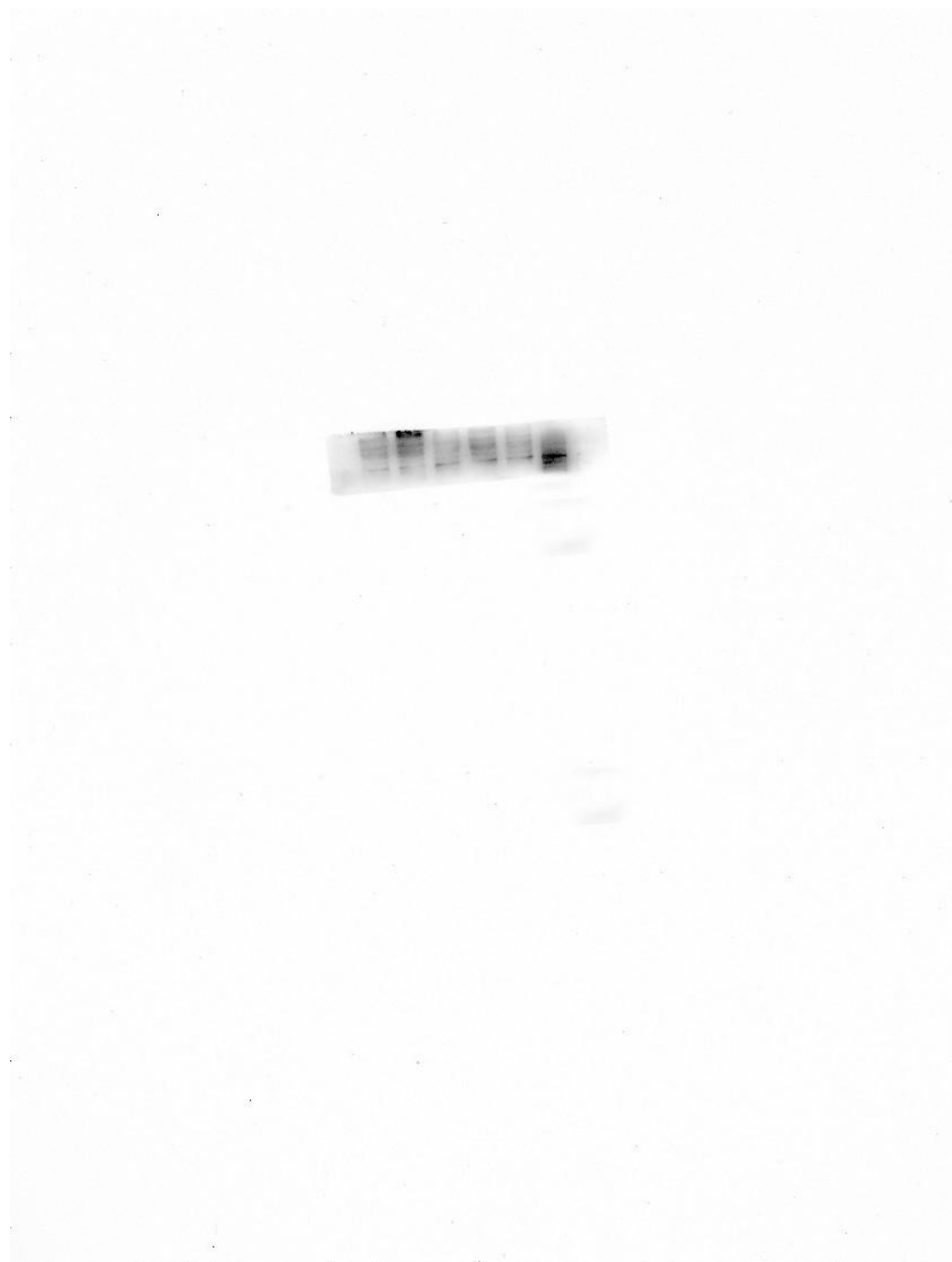

Supplement: Supplementary file 3 — Fig 2- Single Original Western blotting images [file 41419_2023_5623_MOESM3_ESM.zip › Fig 2- Single Original Western blotting images/Fig 2B/03 Cleaved cas3 (6 lanes).pdf]

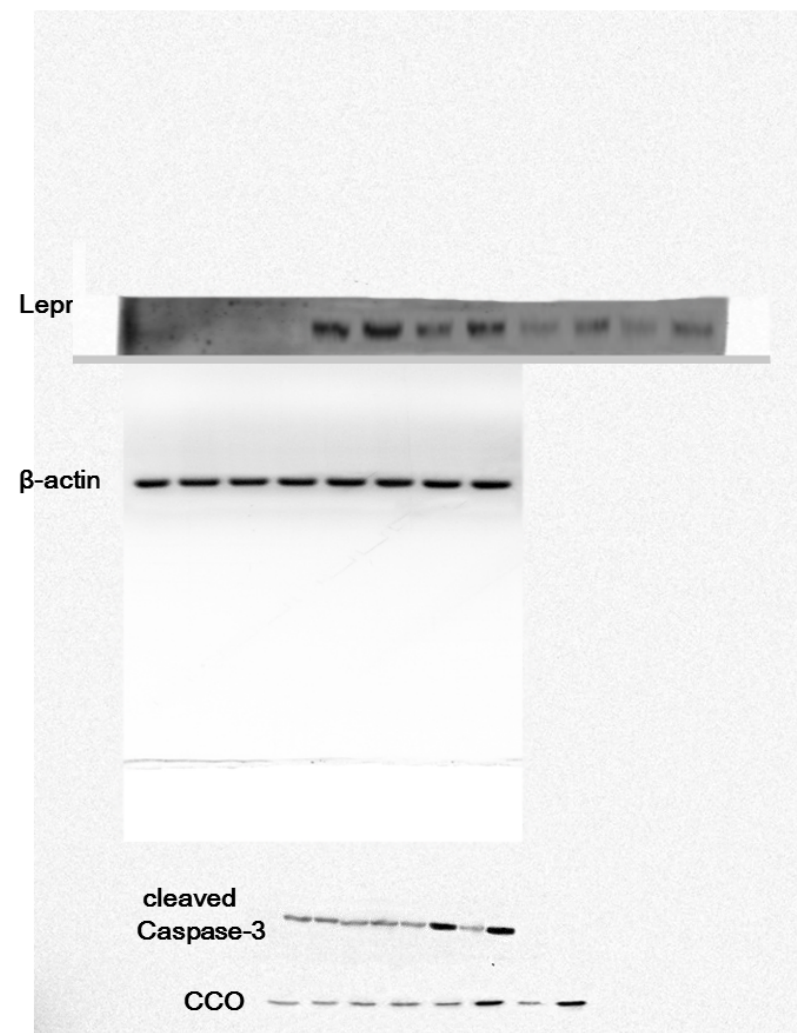

Supplement: Supplementary file 4 — Fig 4- Single Original Western blotting images [file 41419_2023_5623_MOESM4_ESM.zip › Fig 4- Single Original Western blotting images -/Fig 4A (Original film).pdf]

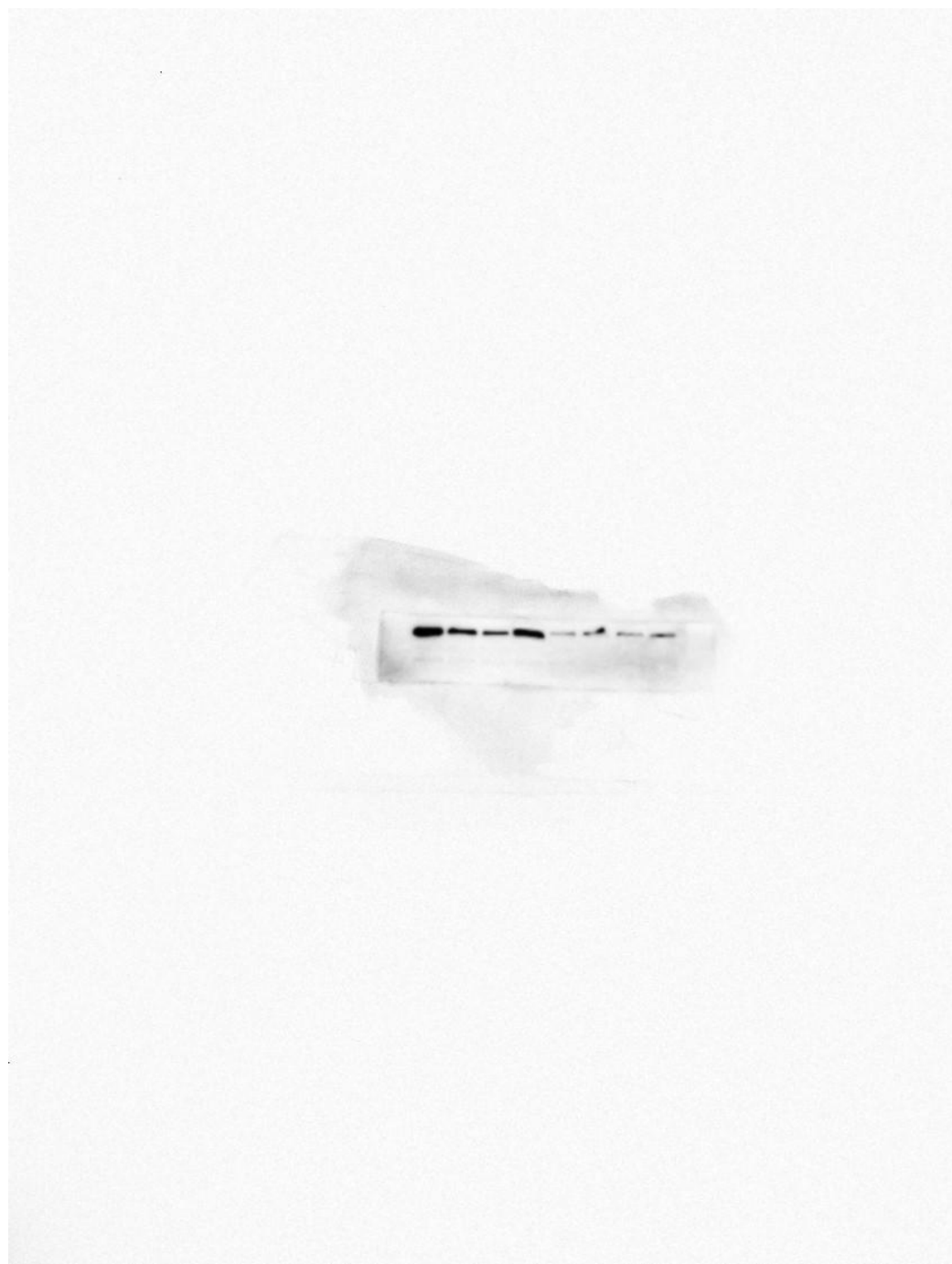

Supplement: Supplementary file 4 — Fig 4- Single Original Western blotting images [file 41419_2023_5623_MOESM4_ESM.zip › Fig 4- Single Original Western blotting images -/Figure 4A/01 Lepr.pdf]

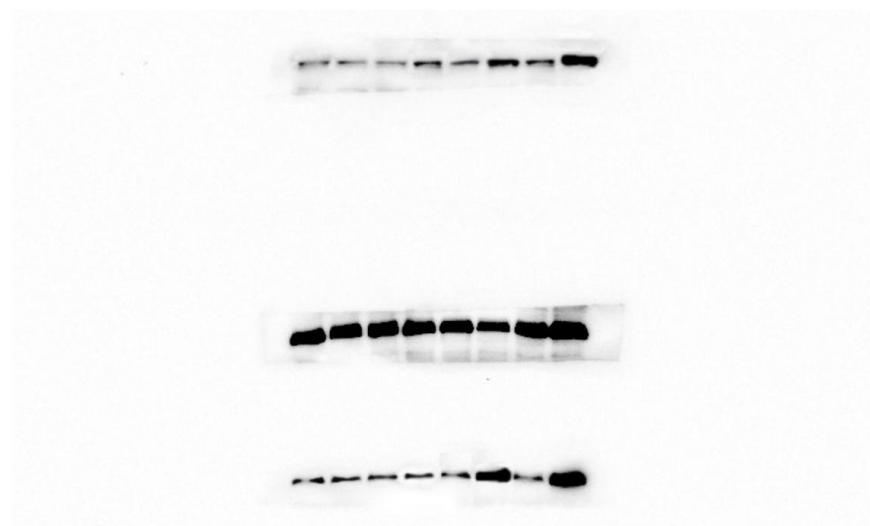

Supplement: Supplementary file 4 — Fig 4- Single Original Western blotting images [file 41419_2023_5623_MOESM4_ESM.zip › Fig 4- Single Original Western blotting images -/Figure 4A/02 CCO & beta-actin & Cleaved cas3.pdf]

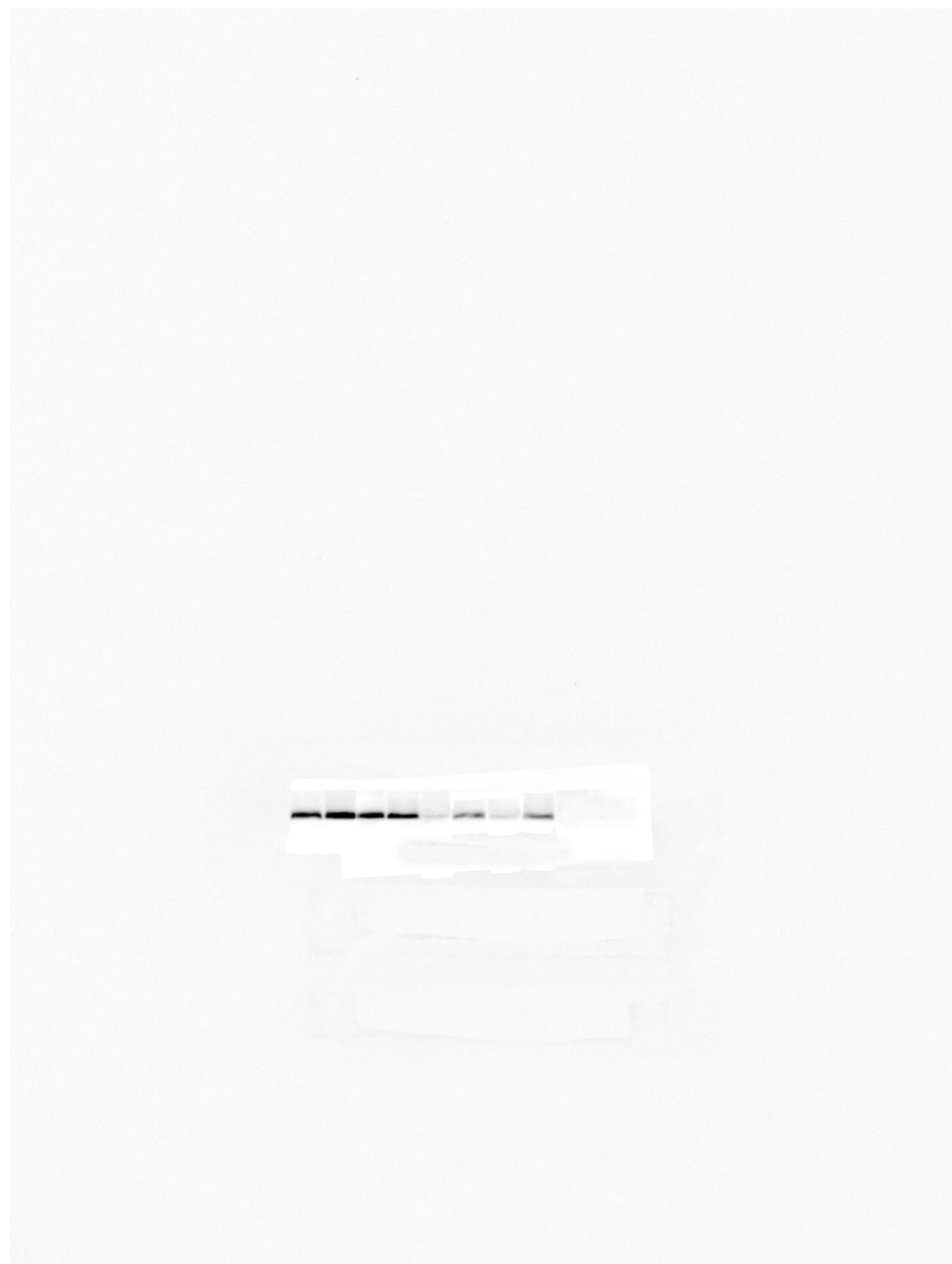

Supplement: Supplementary file 4 — Fig 4- Single Original Western blotting images [file 41419_2023_5623_MOESM4_ESM.zip › Fig 4- Single Original Western blotting images -/Figure 4A/02 Lepr.pdf]

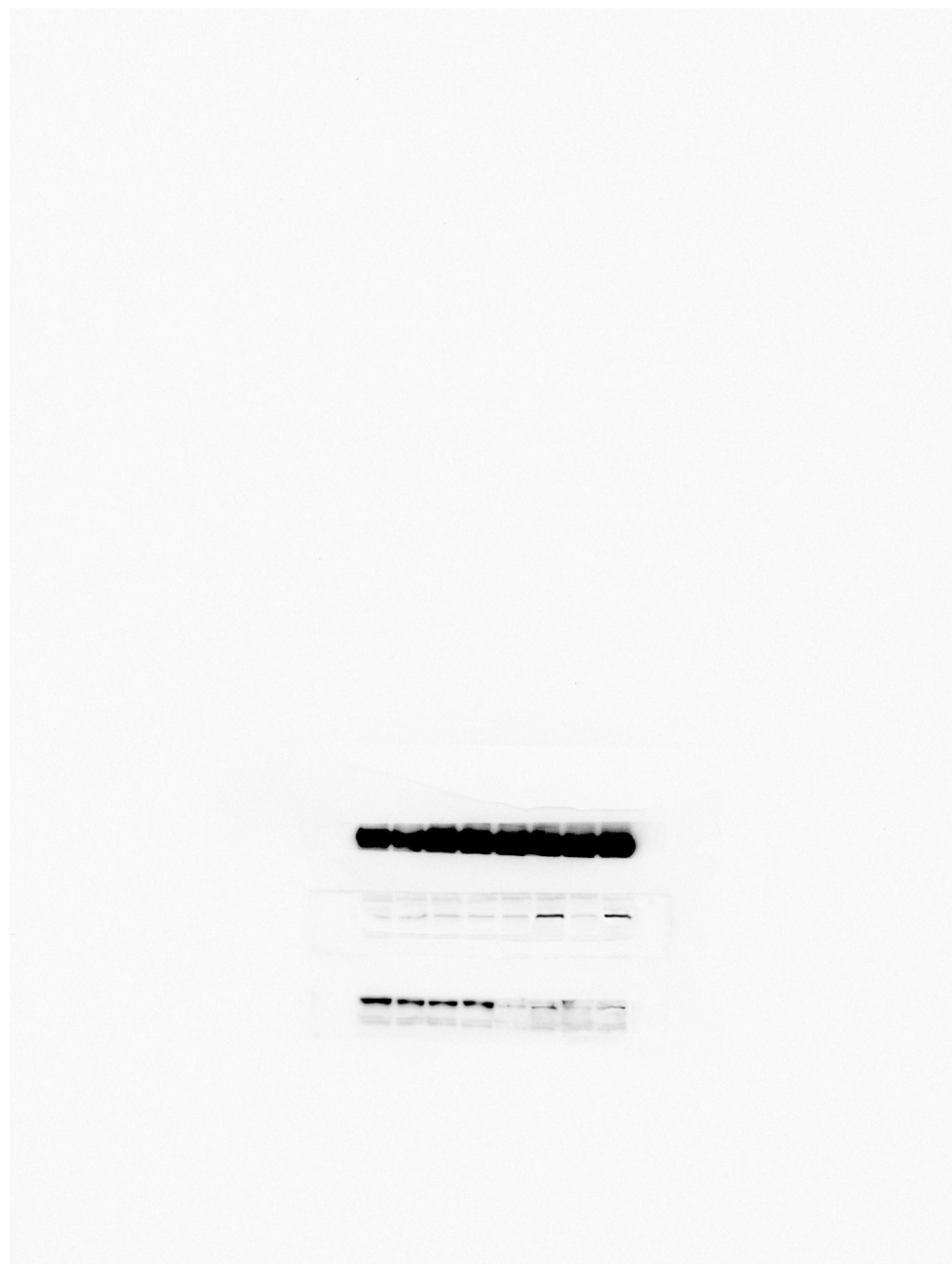

Supplement: Supplementary file 4 — Fig 4- Single Original Western blotting images [file 41419_2023_5623_MOESM4_ESM.zip › Fig 4- Single Original Western blotting images -/Figure 4A/03 beta-actin & CCO & Lepr.pdf]

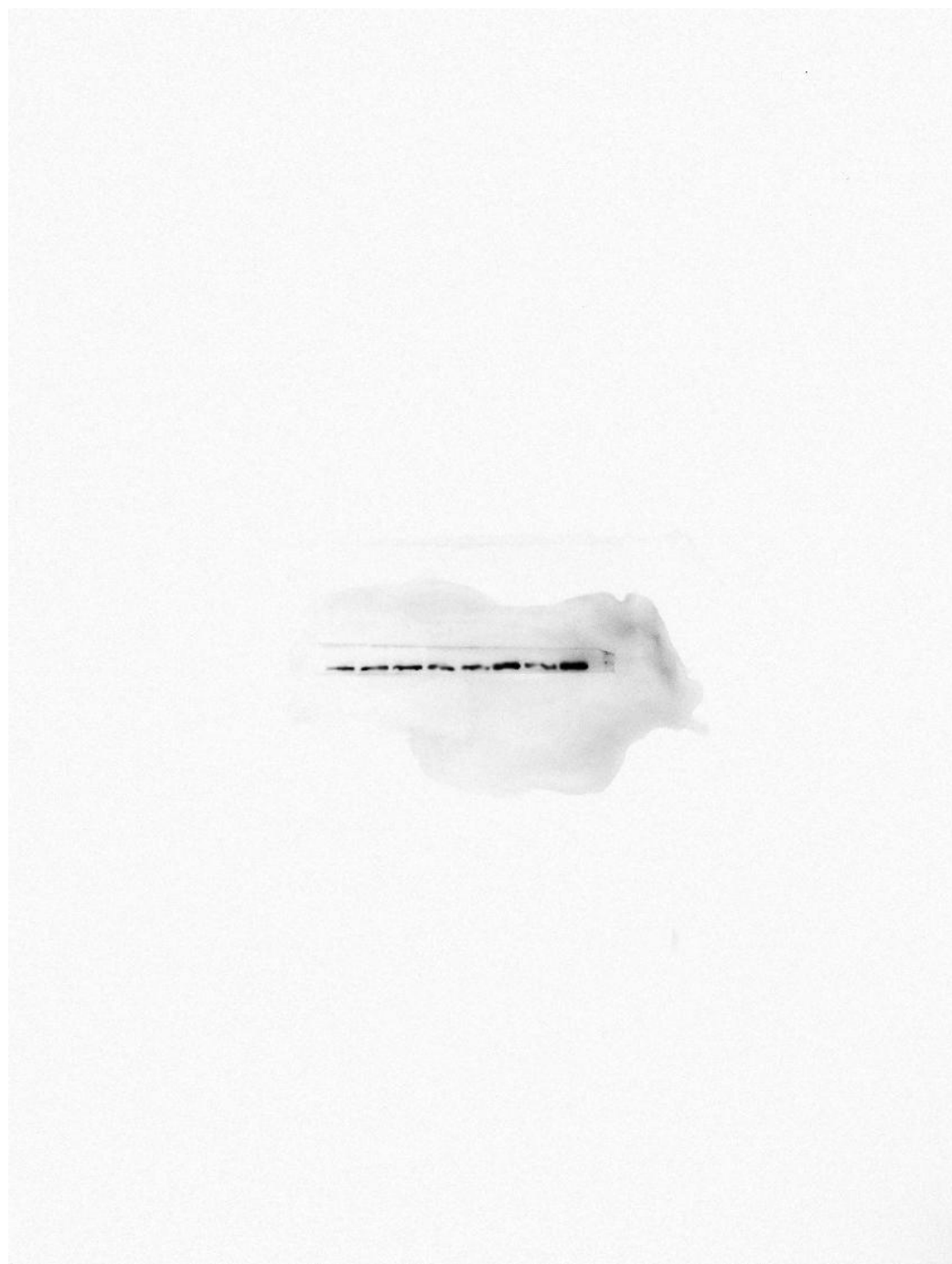

Supplement: Supplementary file 4 — Fig 4- Single Original Western blotting images [file 41419_2023_5623_MOESM4_ESM.zip › Fig 4- Single Original Western blotting images -/Figure 4A/03 Cleaved cas3.pdf]

$\beta$ -actin

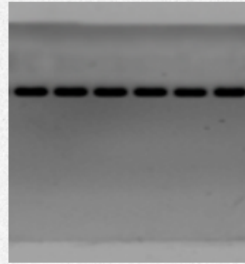

CCO

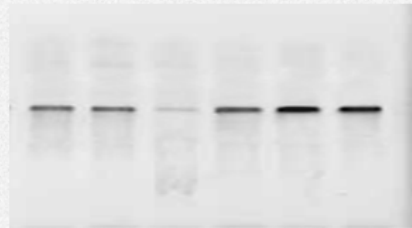

p-AMPK

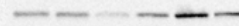

Lepr

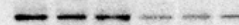

cleaved  
Caspase-3

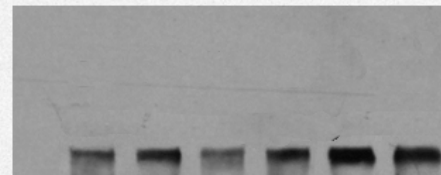

Supplement: Supplementary file 5 — Fig 5-Single Original Western blotting images [file 41419_2023_5623_MOESM5_ESM.zip › Fig 5-Single Original Western blotting images -/Fig 5A (Original film).pdf]

cleaved  
Caspase-3

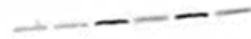

Lepr

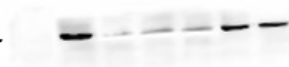

$\beta$ -actin

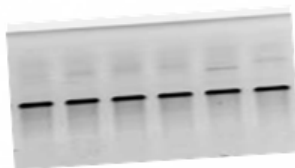

CCO

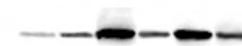

p-AMPK

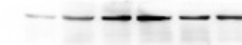

Supplement: Supplementary file 5 — Fig 5-Single Original Western blotting images [file 41419_2023_5623_MOESM5_ESM.zip › Fig 5-Single Original Western blotting images -/Fig 5C (Original film).pdf]

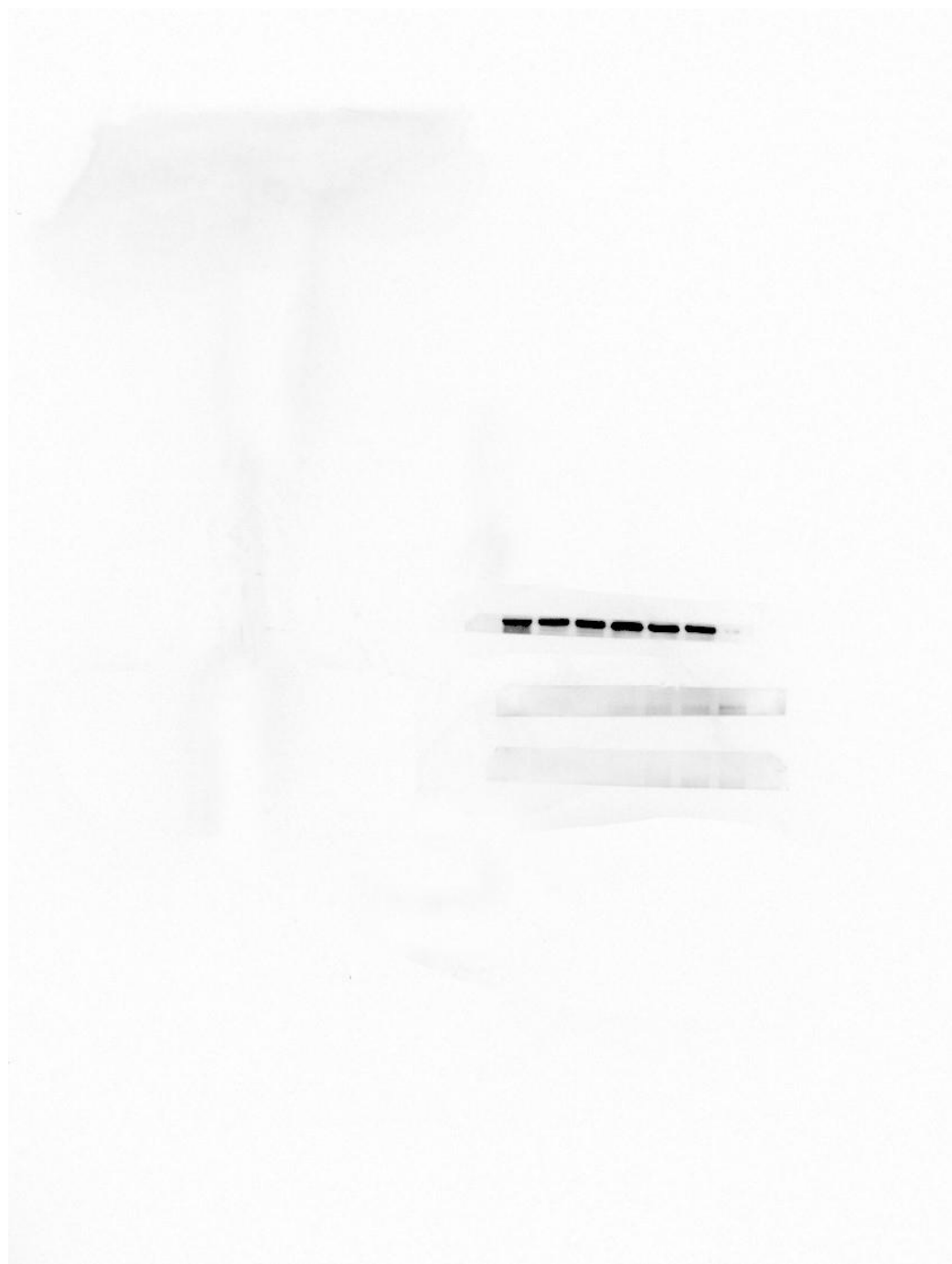

Supplement: Supplementary file 5 — Fig 5-Single Original Western blotting images [file 41419_2023_5623_MOESM5_ESM.zip › Fig 5-Single Original Western blotting images -/Figure 5A/01 beta-actin (Fig 5A 6 lanes).pdf]

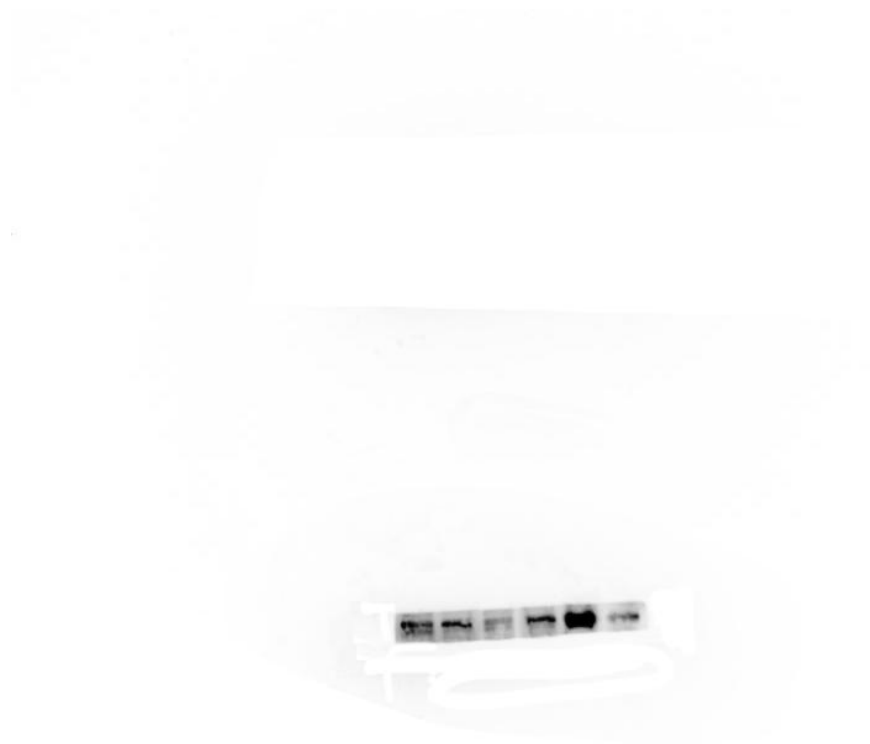

Supplement: Supplementary file 5 — Fig 5-Single Original Western blotting images [file 41419_2023_5623_MOESM5_ESM.zip › Fig 5-Single Original Western blotting images -/Figure 5A/01 Cleaved cas3 (Fig 5A 6 lanes).pdf]

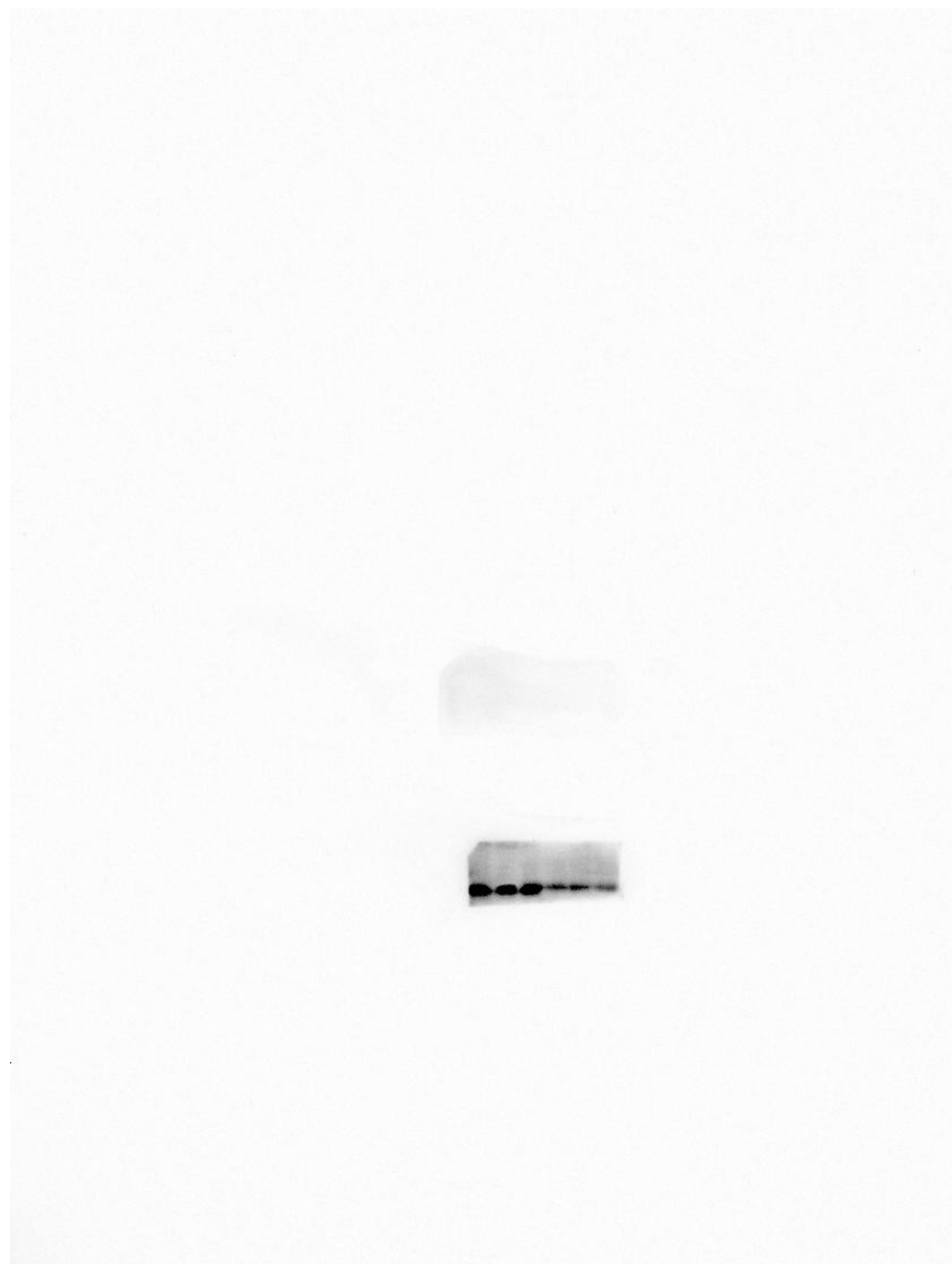

Supplement: Supplementary file 5 — Fig 5-Single Original Western blotting images [file 41419_2023_5623_MOESM5_ESM.zip › Fig 5-Single Original Western blotting images -/Figure 5A/01 Lepr (Fig 5A 6 lanes).pdf]

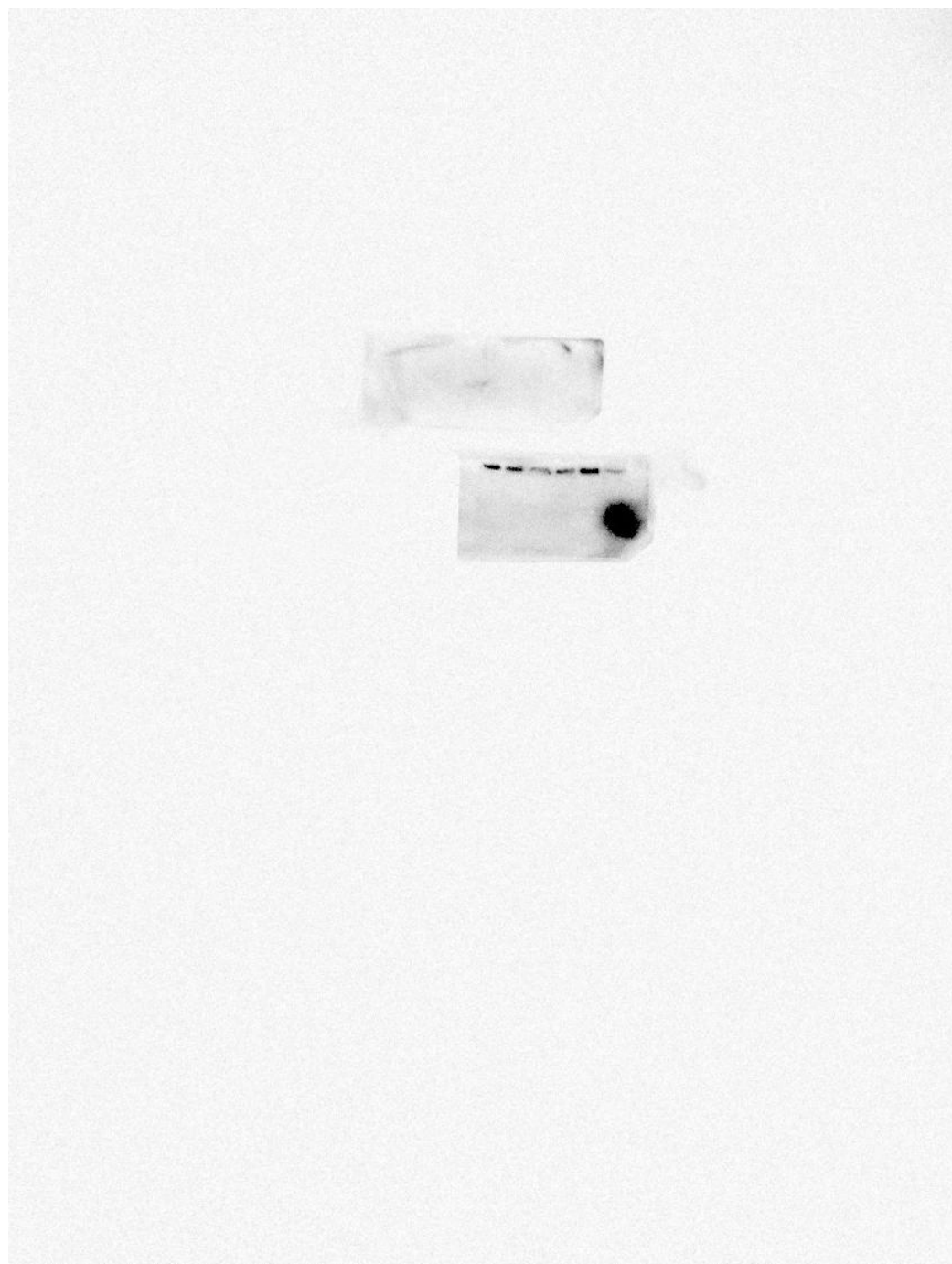

Supplement: Supplementary file 5 — Fig 5-Single Original Western blotting images [file 41419_2023_5623_MOESM5_ESM.zip › Fig 5-Single Original Western blotting images -/Figure 5A/01 pAMPK (Fig 5A 6 lanes).pdf]

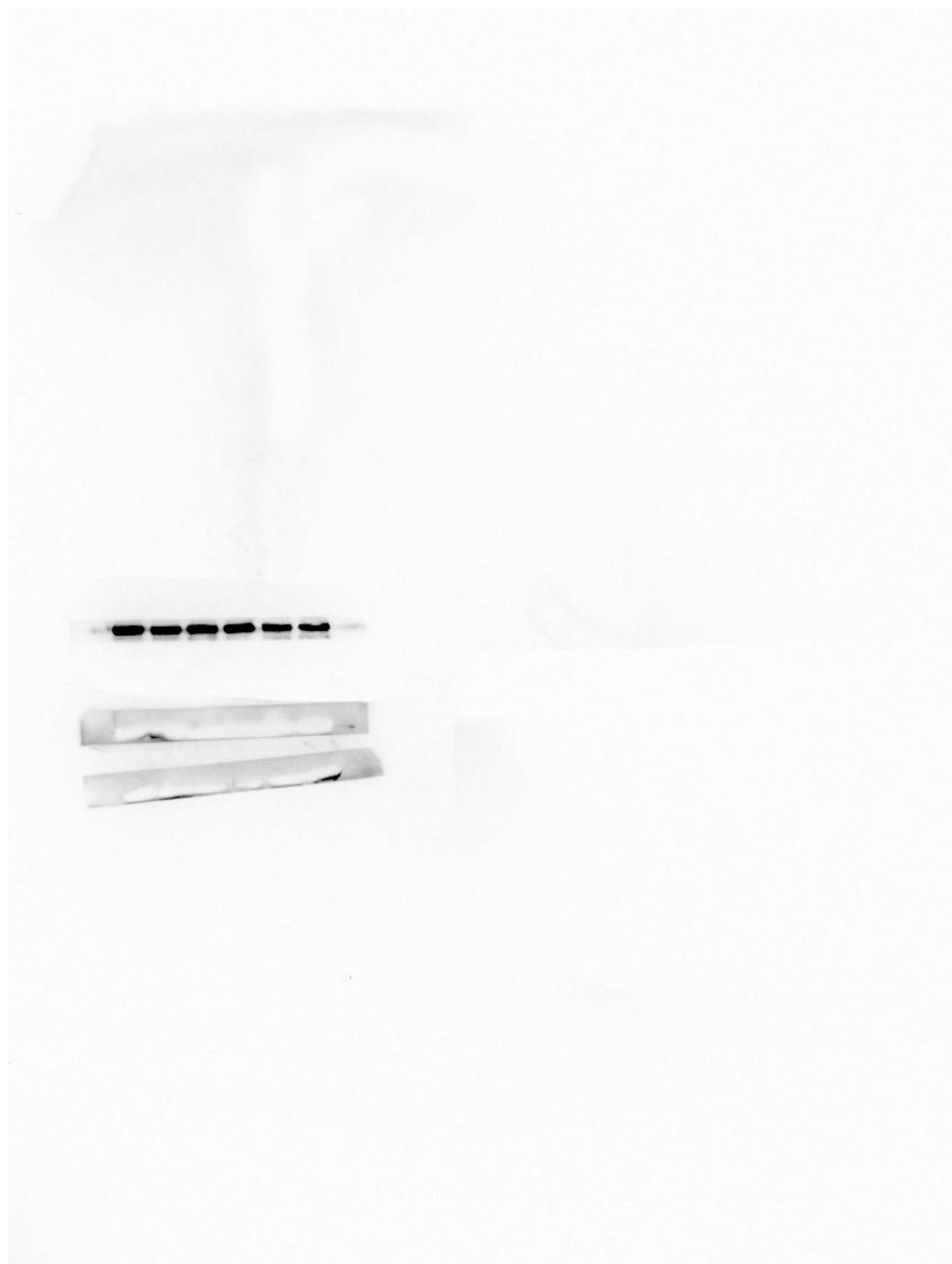

Supplement: Supplementary file 5 — Fig 5-Single Original Western blotting images [file 41419_2023_5623_MOESM5_ESM.zip › Fig 5-Single Original Western blotting images -/Figure 5A/02 beta-actin (6 lanes).pdf]

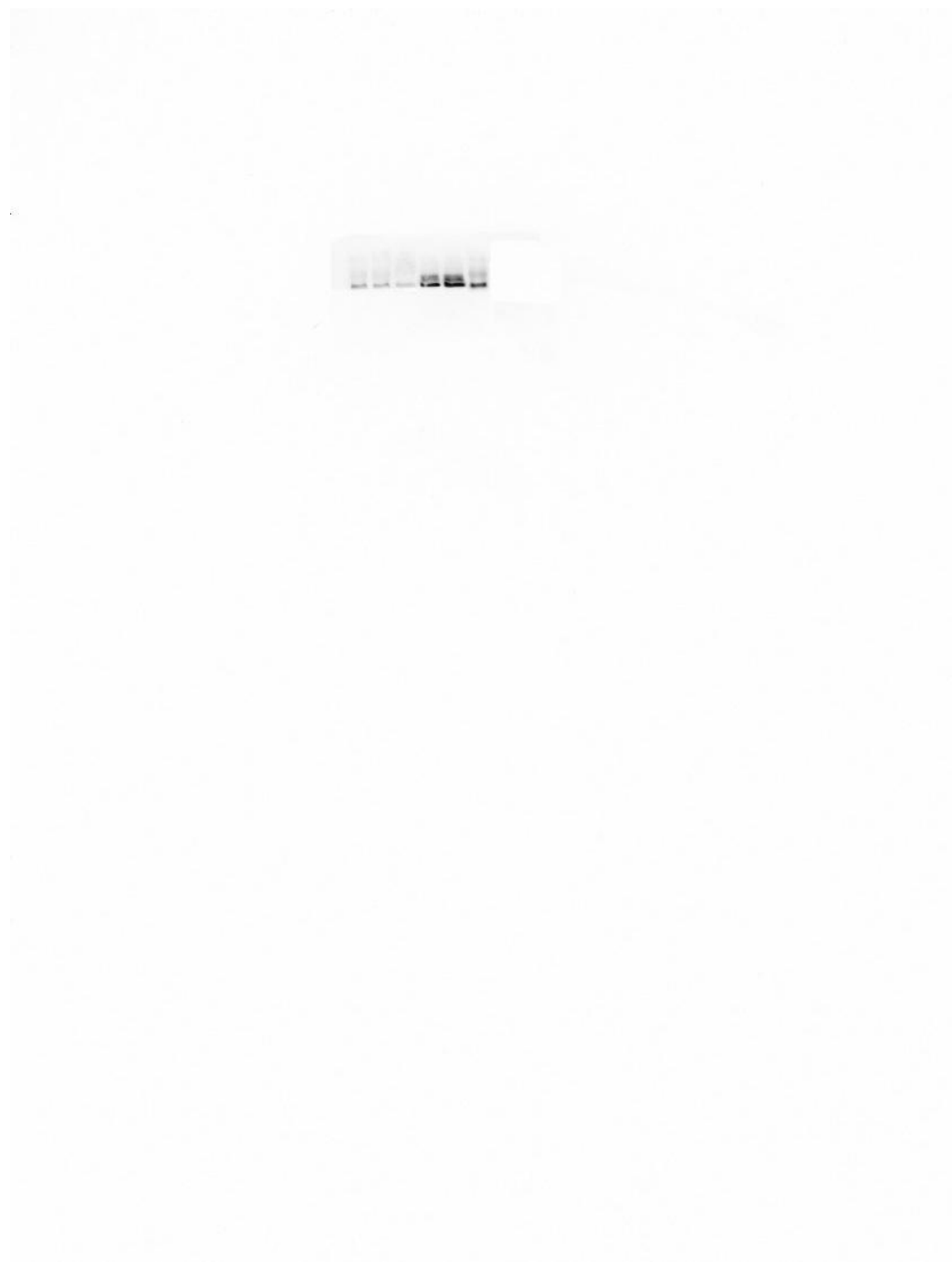

Supplement: Supplementary file 5 — Fig 5-Single Original Western blotting images [file 41419_2023_5623_MOESM5_ESM.zip › Fig 5-Single Original Western blotting images -/Figure 5A/02 CCO (Fig 5A 6 lanes).pdf]

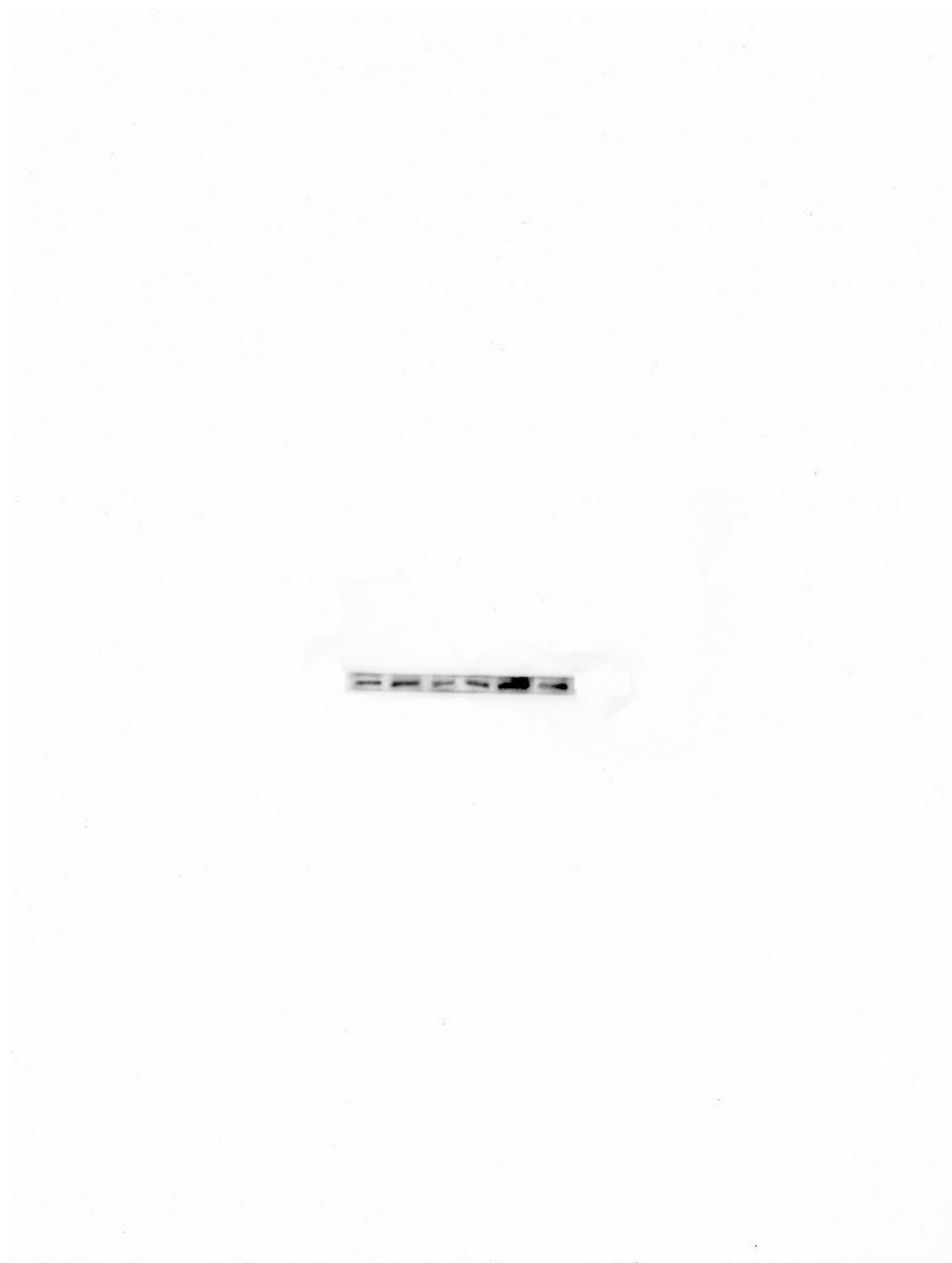

Supplement: Supplementary file 5 — Fig 5-Single Original Western blotting images [file 41419_2023_5623_MOESM5_ESM.zip › Fig 5-Single Original Western blotting images -/Figure 5A/02 Cleaved cas3 (Fig 5A 6 lanes).pdf]

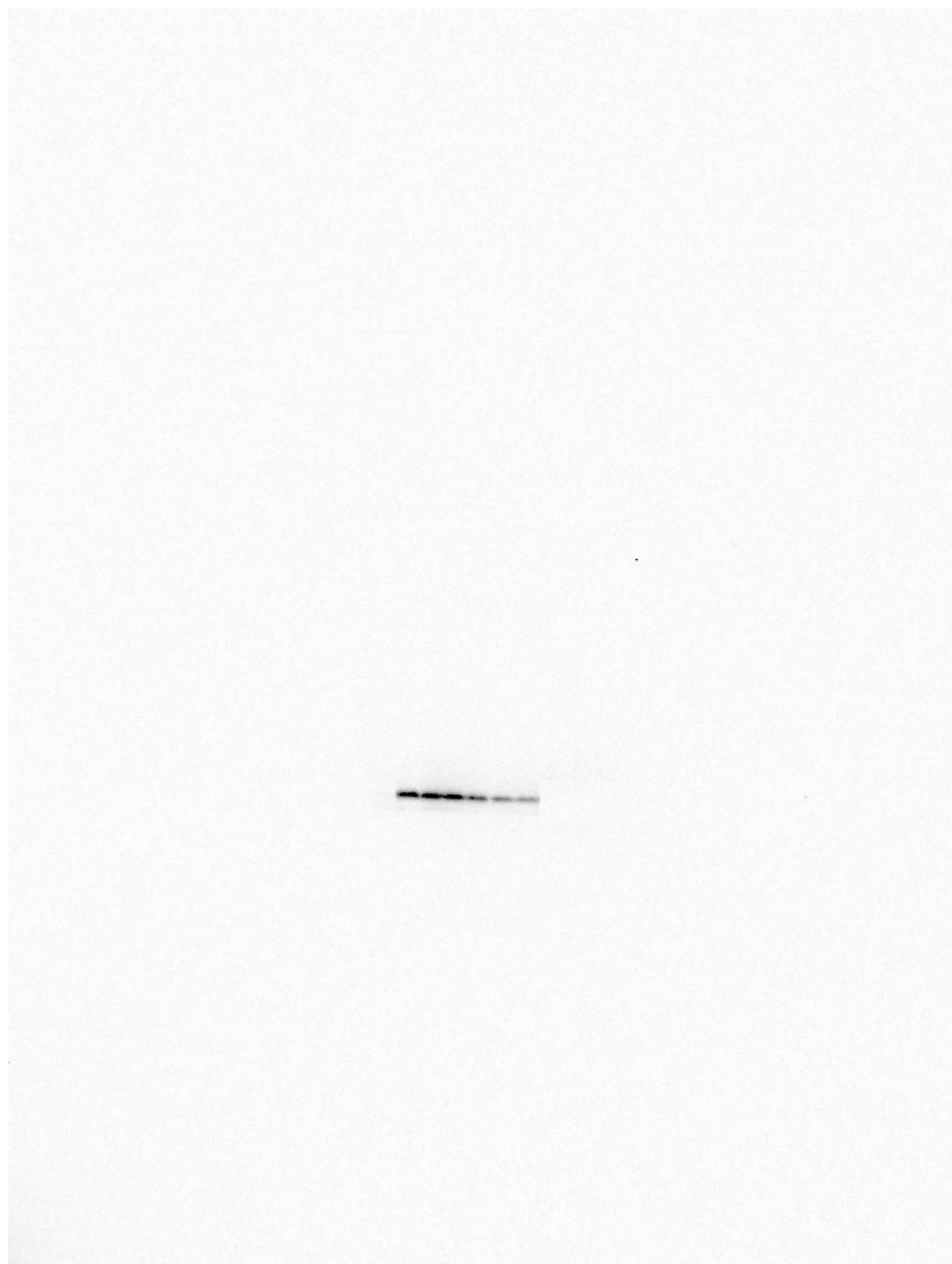

Supplement: Supplementary file 5 — Fig 5-Single Original Western blotting images [file 41419_2023_5623_MOESM5_ESM.zip › Fig 5-Single Original Western blotting images -/Figure 5A/02 Lepr (Fig 5A 6 lanes).pdf]

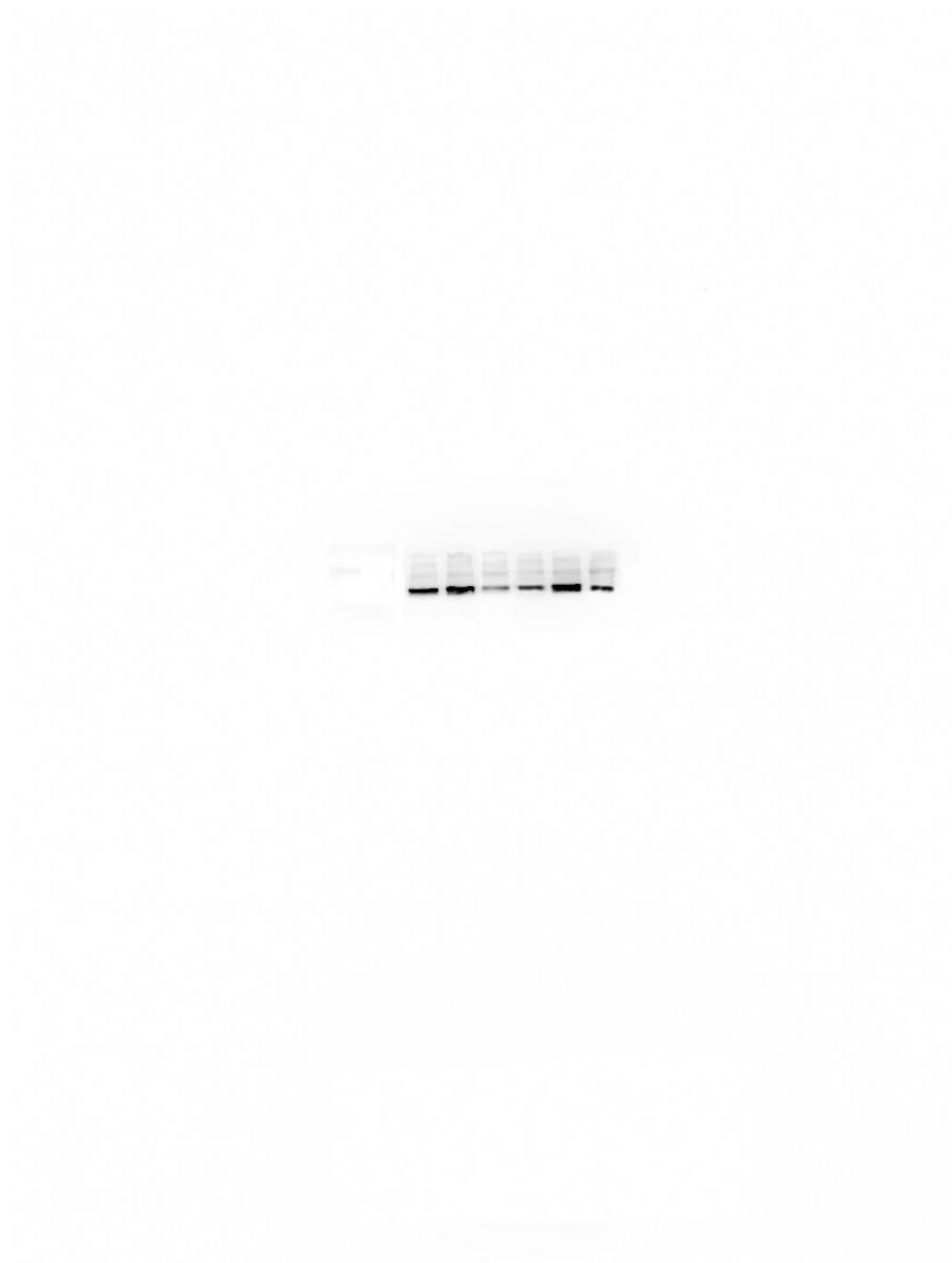

Supplement: Supplementary file 5 — Fig 5-Single Original Western blotting images [file 41419_2023_5623_MOESM5_ESM.zip › Fig 5-Single Original Western blotting images -/Figure 5A/02 pAMPK (Fig 5A 6 lanes).pdf]

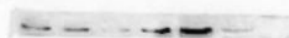

Supplement: Supplementary file 5 — Fig 5-Single Original Western blotting images [file 41419_2023_5623_MOESM5_ESM.zip › Fig 5-Single Original Western blotting images -/Figure 5A/03 CCO (Fig 5A 6 lanes).pdf]

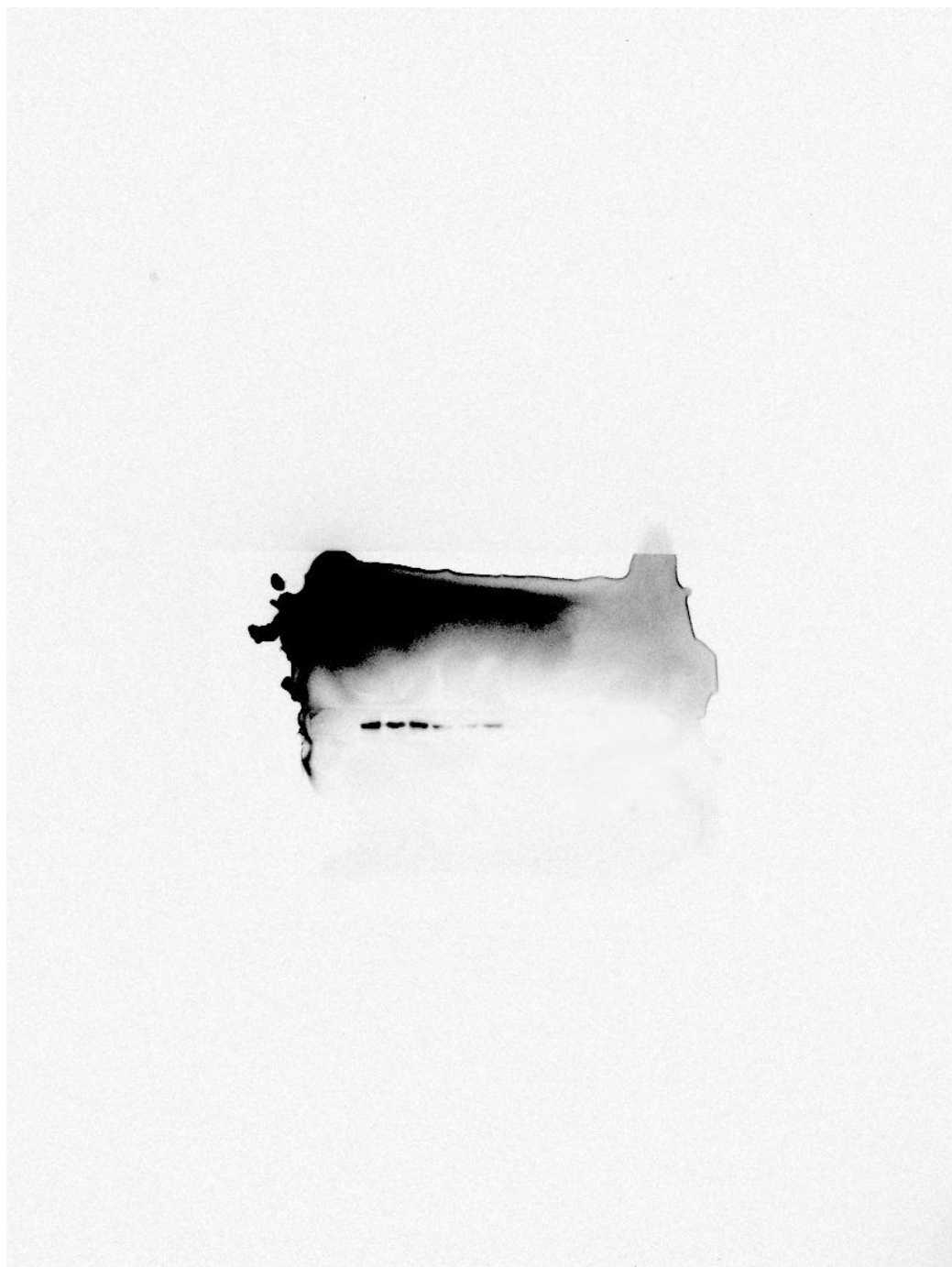

Supplement: Supplementary file 5 — Fig 5-Single Original Western blotting images [file 41419_2023_5623_MOESM5_ESM.zip › Fig 5-Single Original Western blotting images -/Figure 5A/03 Lepr (Fig 5A 6 lanes).pdf]

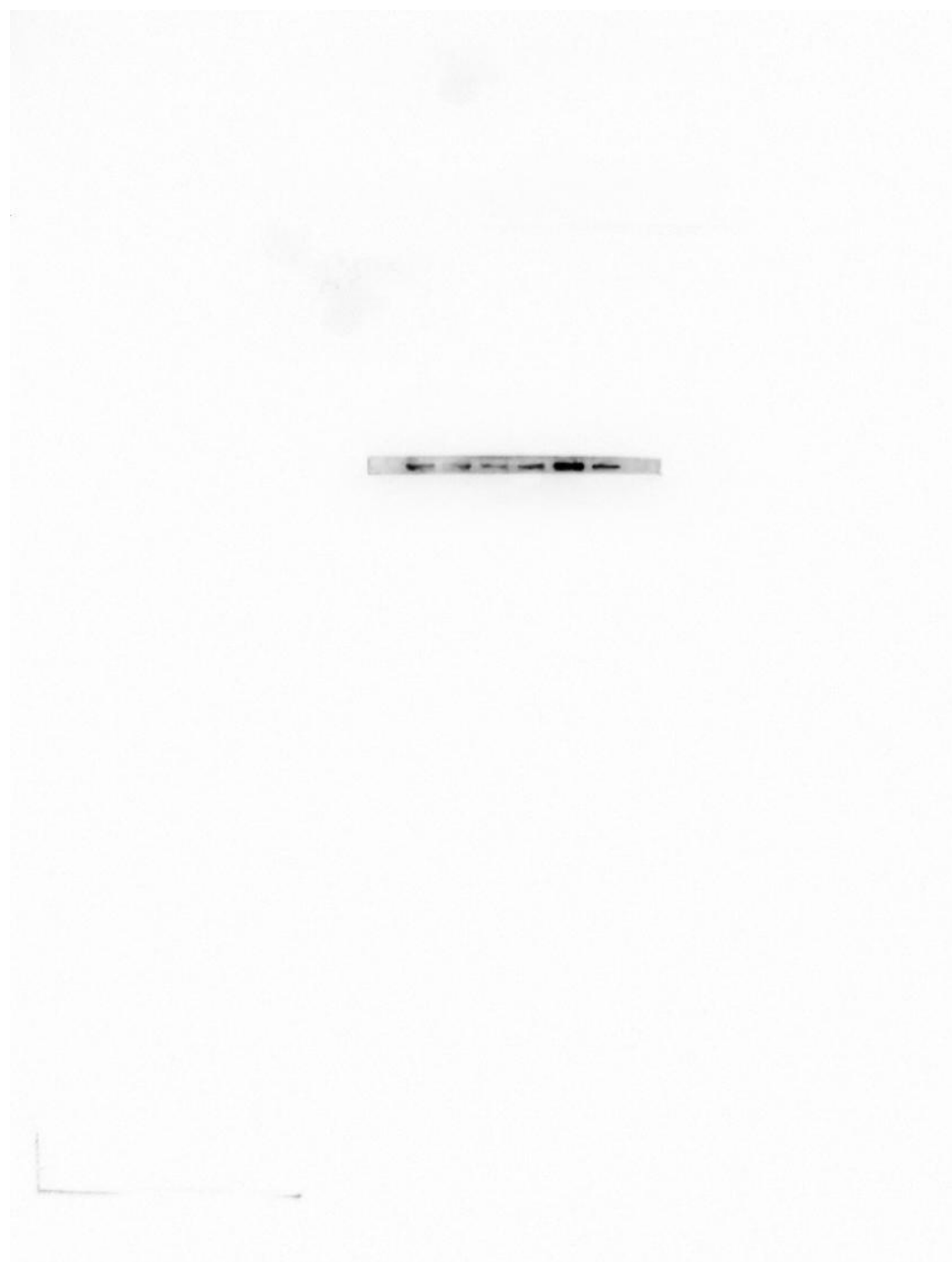

Supplement: Supplementary file 5 — Fig 5-Single Original Western blotting images [file 41419_2023_5623_MOESM5_ESM.zip › Fig 5-Single Original Western blotting images -/Figure 5A/03 pAMPK (Fig 5A 6 lanes).pdf]

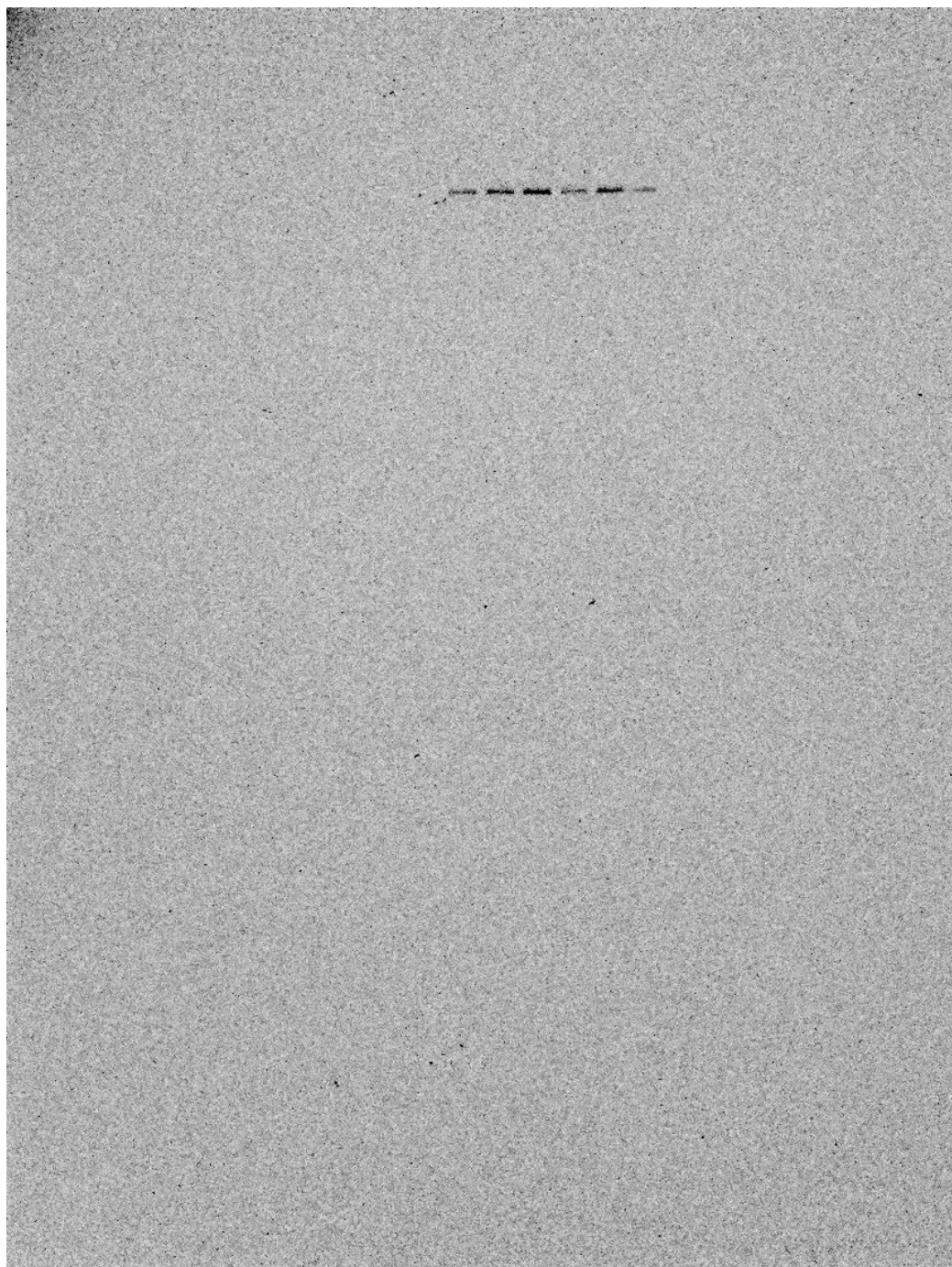

Supplement: Supplementary file 5 — Fig 5-Single Original Western blotting images [file 41419_2023_5623_MOESM5_ESM.zip › Fig 5-Single Original Western blotting images -/Figure 5C/01 CCO (Fig 5C 6 lanes).pdf]

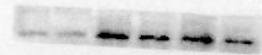

Supplement: Supplementary file 5 — Fig 5-Single Original Western blotting images [file 41419_2023_5623_MOESM5_ESM.zip › Fig 5-Single Original Western blotting images -/Figure 5C/01 Cleaved cas3 (Fig 5C 6 lanes).pdf]

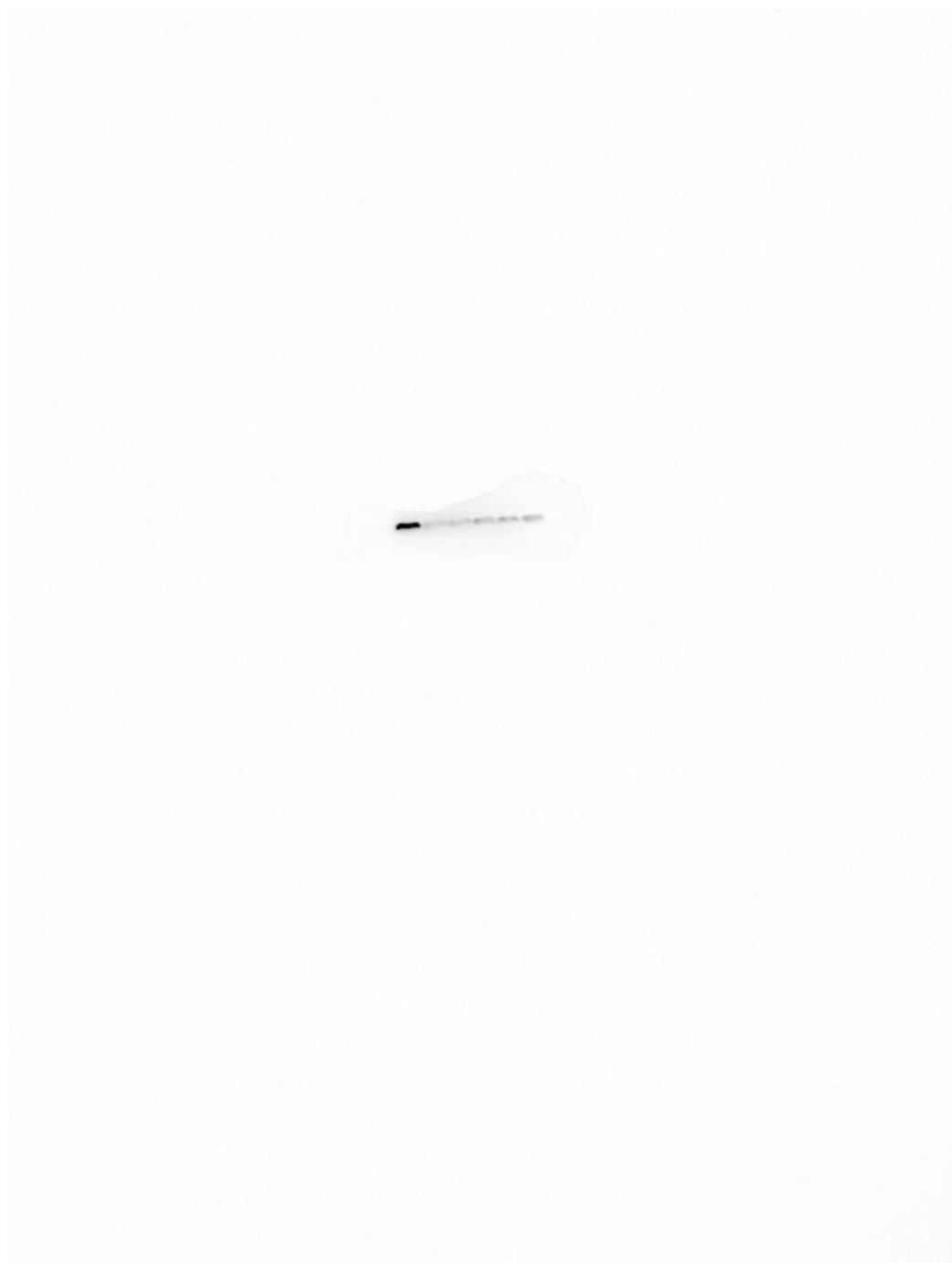

Supplement: Supplementary file 5 — Fig 5-Single Original Western blotting images [file 41419_2023_5623_MOESM5_ESM.zip › Fig 5-Single Original Western blotting images -/Figure 5C/01 Lepr (Fig 5C 6 lanes).pdf]

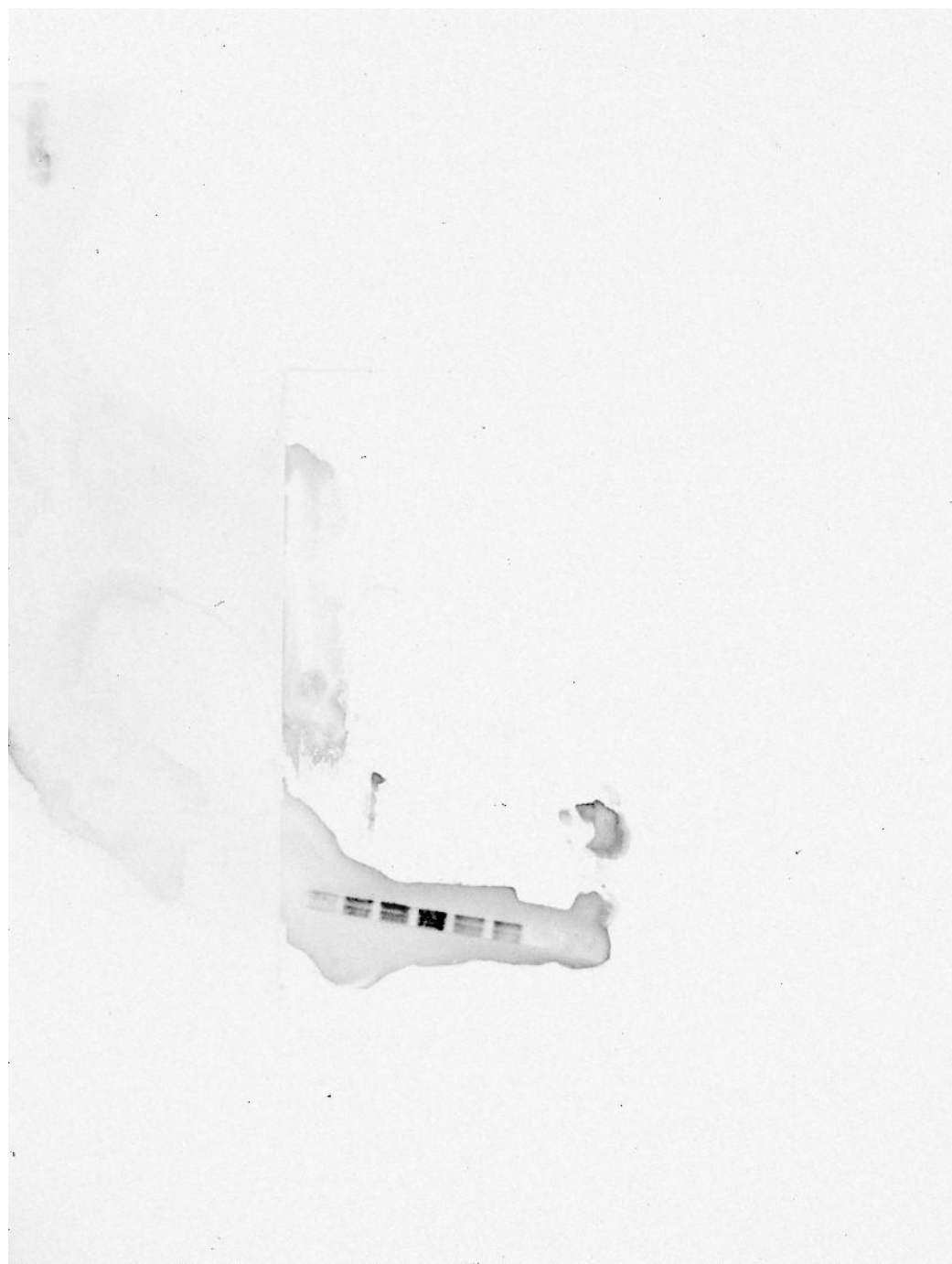

Supplement: Supplementary file 5 — Fig 5-Single Original Western blotting images [file 41419_2023_5623_MOESM5_ESM.zip › Fig 5-Single Original Western blotting images -/Figure 5C/01 pAMPK (Fig 5C 6 lanes).pdf]

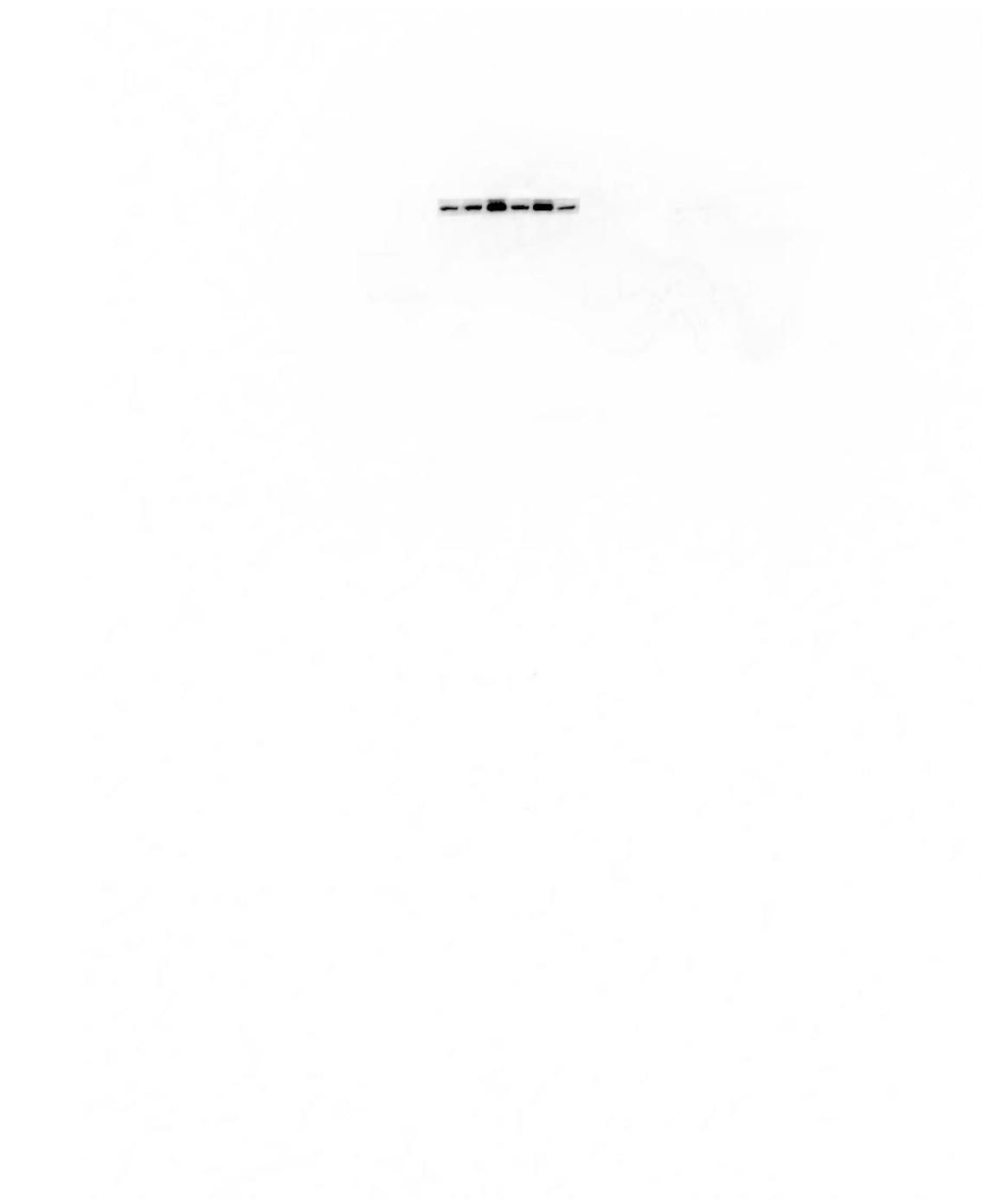

Supplement: Supplementary file 5 — Fig 5-Single Original Western blotting images [file 41419_2023_5623_MOESM5_ESM.zip › Fig 5-Single Original Western blotting images -/Figure 5C/02 CCO (Fig 5C 6 lanes).pdf]

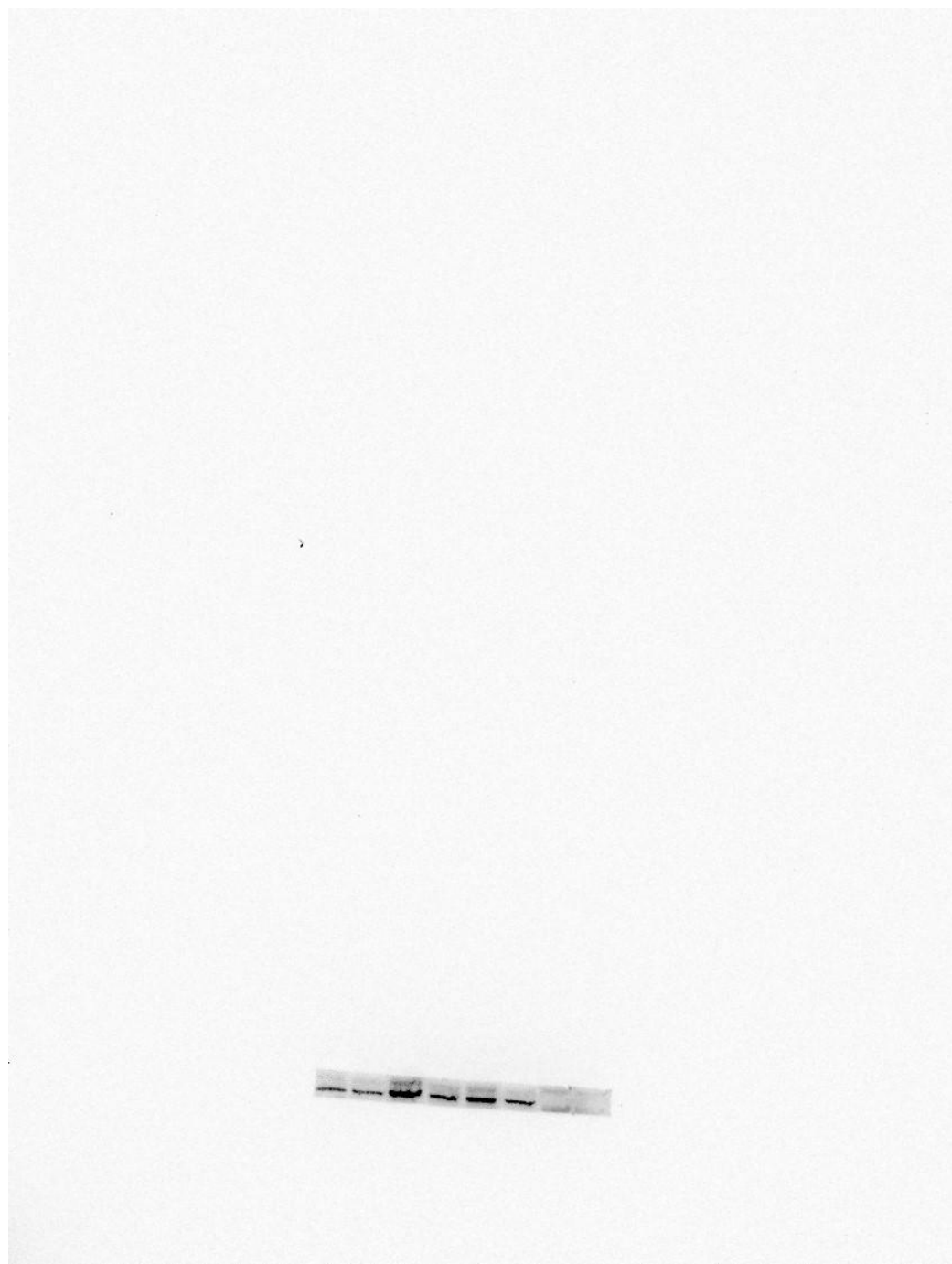

Supplement: Supplementary file 5 — Fig 5-Single Original Western blotting images [file 41419_2023_5623_MOESM5_ESM.zip › Fig 5-Single Original Western blotting images -/Figure 5C/02 Cleaved cas3.pdf]

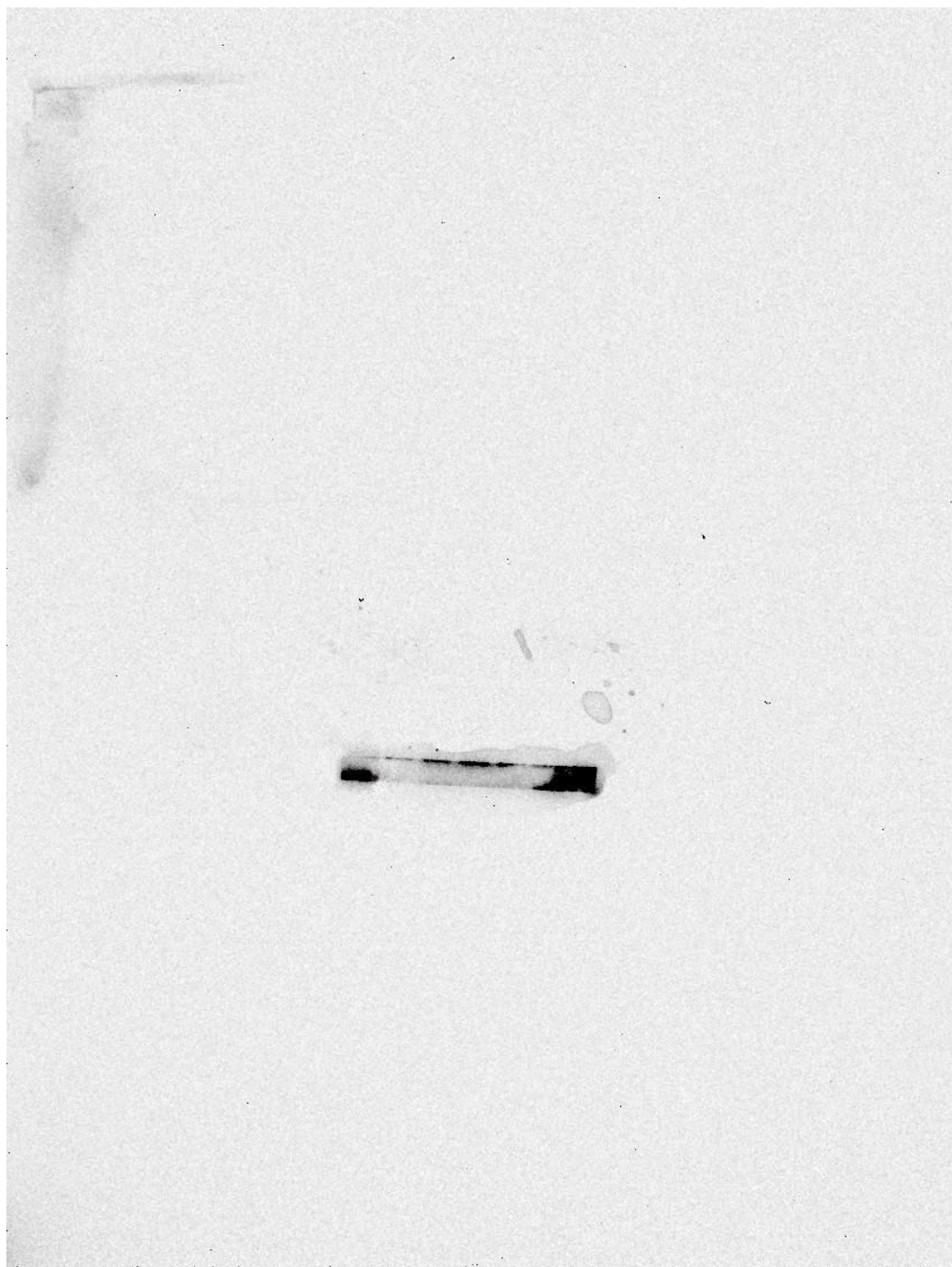

Supplement: Supplementary file 5 — Fig 5-Single Original Western blotting images [file 41419_2023_5623_MOESM5_ESM.zip › Fig 5-Single Original Western blotting images -/Figure 5C/02 pAMPK (Fig 5C 6 lanes).pdf]

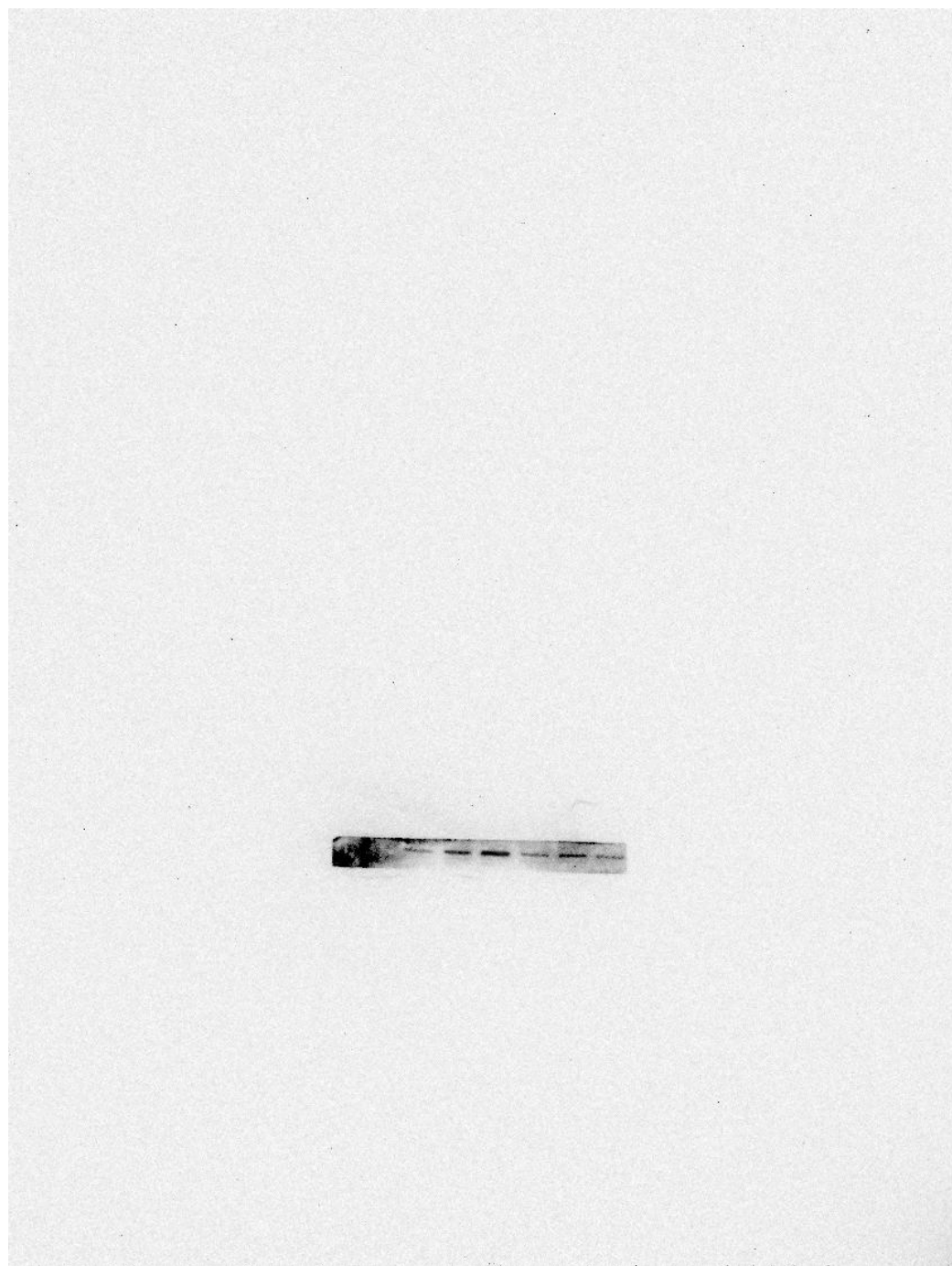

Supplement: Supplementary file 5 — Fig 5-Single Original Western blotting images [file 41419_2023_5623_MOESM5_ESM.zip › Fig 5-Single Original Western blotting images -/Figure 5C/03 CCO (Fig 5C 6 lanes).pdf]

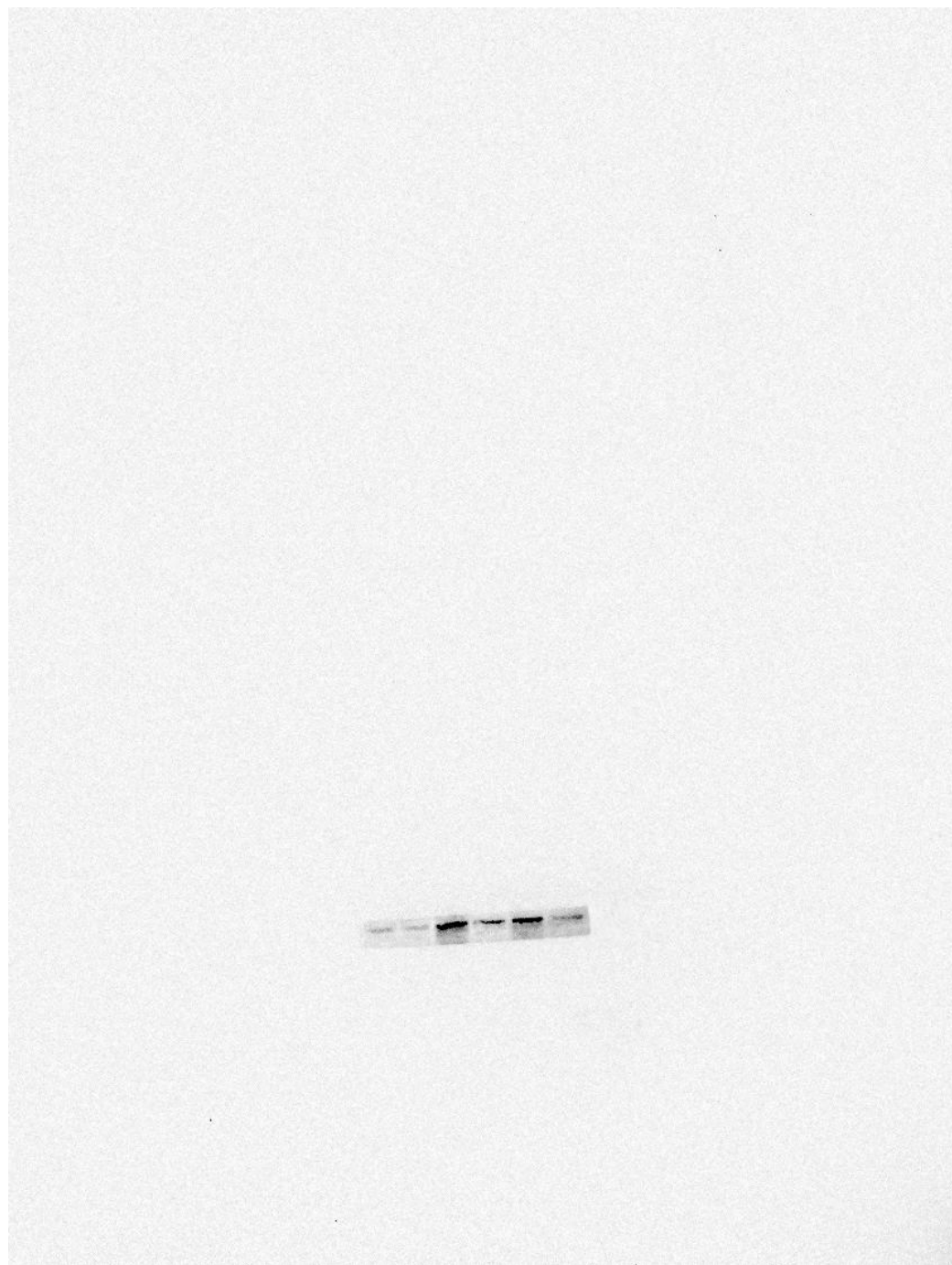

Supplement: Supplementary file 5 — Fig 5-Single Original Western blotting images [file 41419_2023_5623_MOESM5_ESM.zip › Fig 5-Single Original Western blotting images -/Figure 5C/03 Cleaved cas3.pdf]

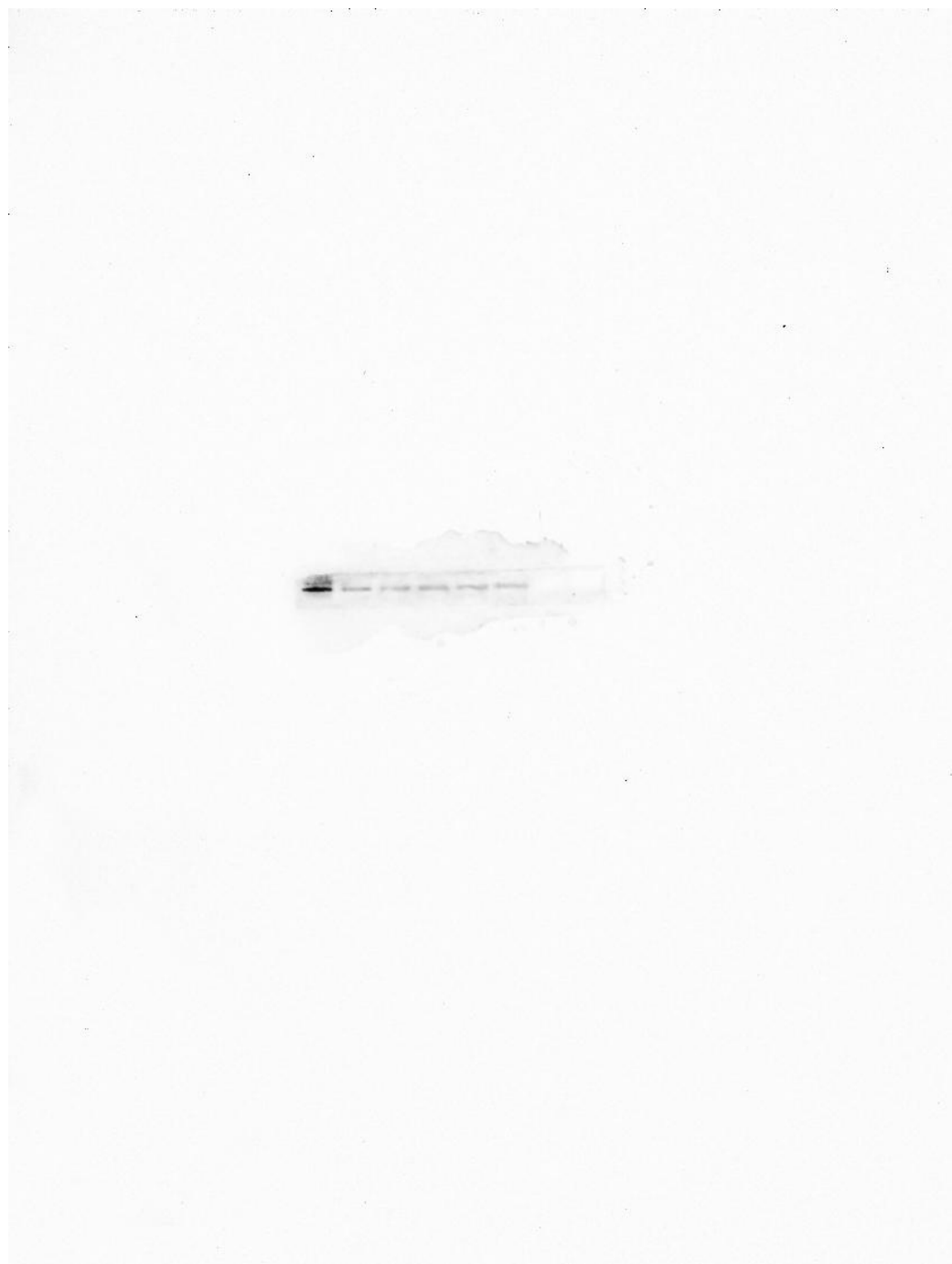

Supplement: Supplementary file 5 — Fig 5-Single Original Western blotting images [file 41419_2023_5623_MOESM5_ESM.zip › Fig 5-Single Original Western blotting images -/Figure 5C/03 Lepr (Fig 5C 6 lanes).pdf]

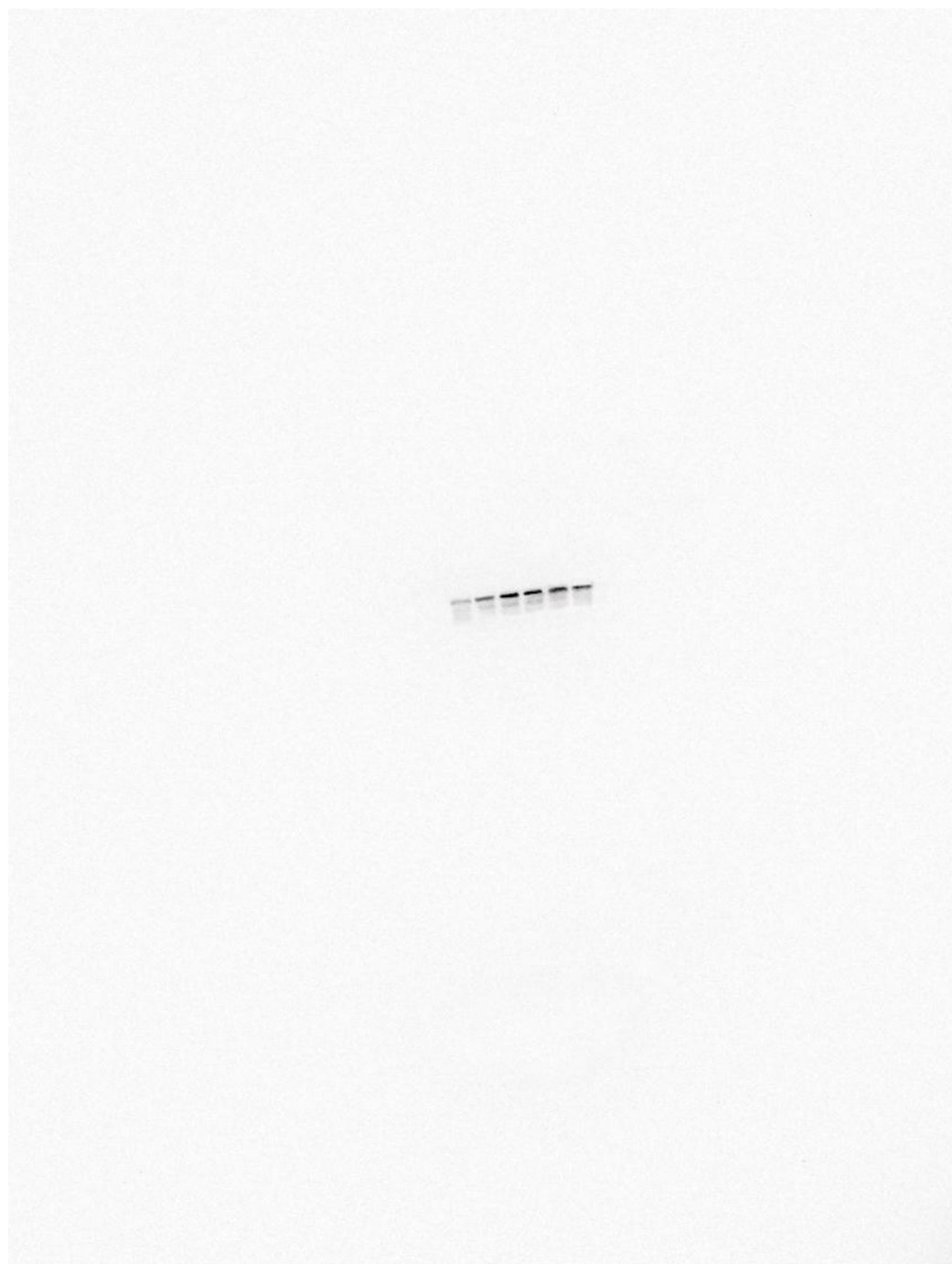

Supplement: Supplementary file 5 — Fig 5-Single Original Western blotting images [file 41419_2023_5623_MOESM5_ESM.zip › Fig 5-Single Original Western blotting images -/Figure 5C/03 pAMPK (Fig 5C 6 lanes).pdf]

Lepr

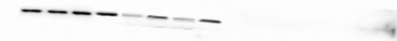

$\beta$ -actin

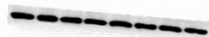

CCO

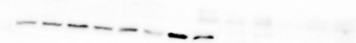

Bax

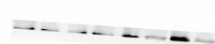

p-AMPK

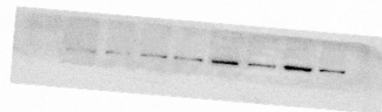

Supplement: Supplementary file 6 — Fig 7-Single Original Western blotting images [file 41419_2023_5623_MOESM6_ESM.zip › Fig 7-Single Original Western blotting images -/Figure 7 (Original film) 01.pdf]

Bcl-2

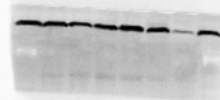

cleaved  
Caspase-3

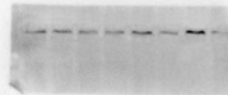

Supplement: Supplementary file 6 — Fig 7-Single Original Western blotting images [file 41419_2023_5623_MOESM6_ESM.zip › Fig 7-Single Original Western blotting images -/Figure 7 (Original film) 02.pdf]

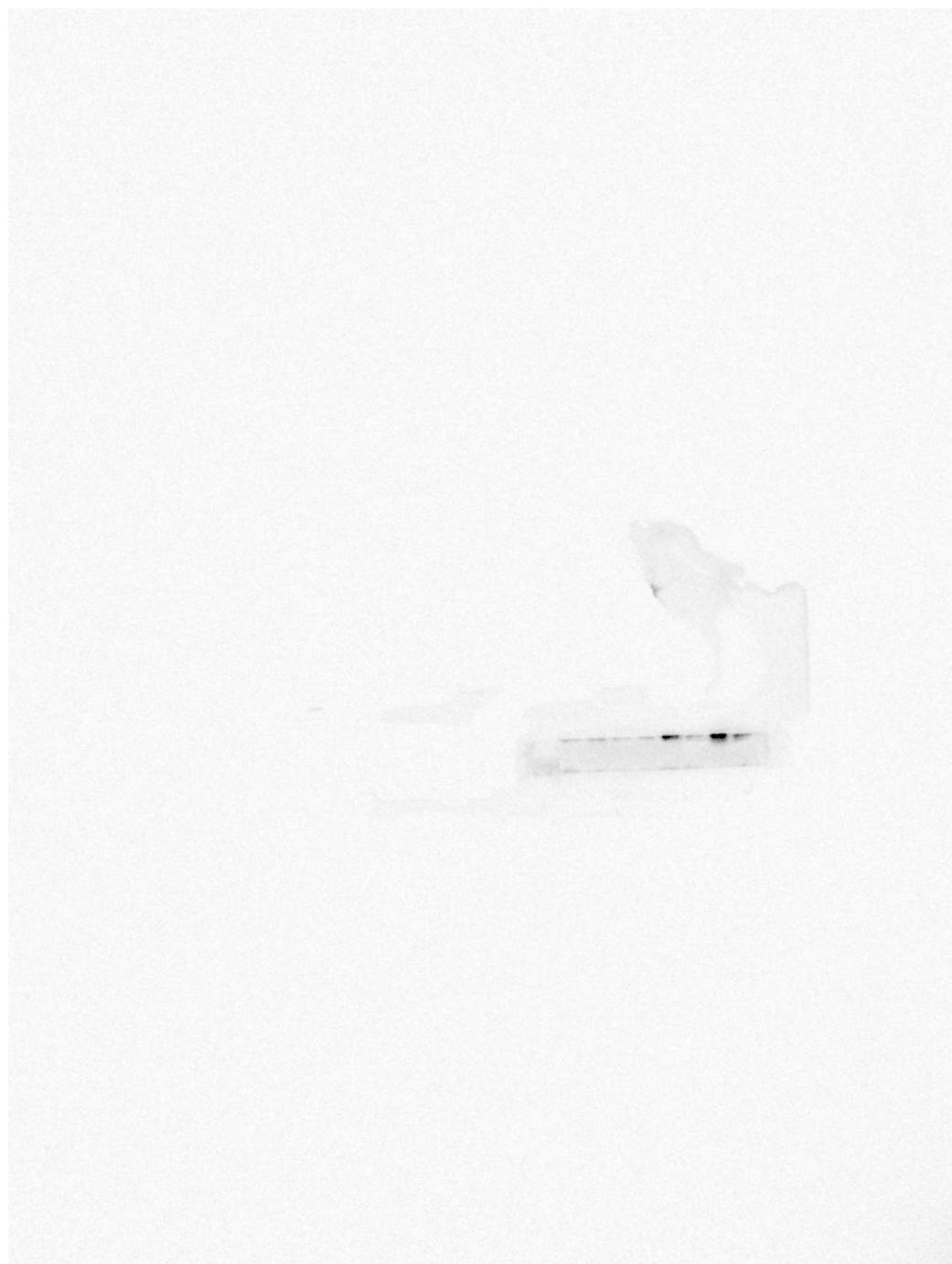

Supplement: Supplementary file 6 — Fig 7-Single Original Western blotting images [file 41419_2023_5623_MOESM6_ESM.zip › Fig 7-Single Original Western blotting images -/Figure 7B/01 Bax (Fig 7B).pdf]

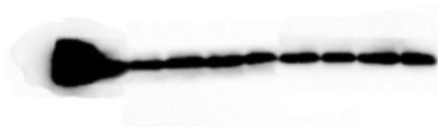

Supplement: Supplementary file 6 — Fig 7-Single Original Western blotting images [file 41419_2023_5623_MOESM6_ESM.zip › Fig 7-Single Original Western blotting images -/Figure 7B/01 beta-actin (Fig 7B).pdf]

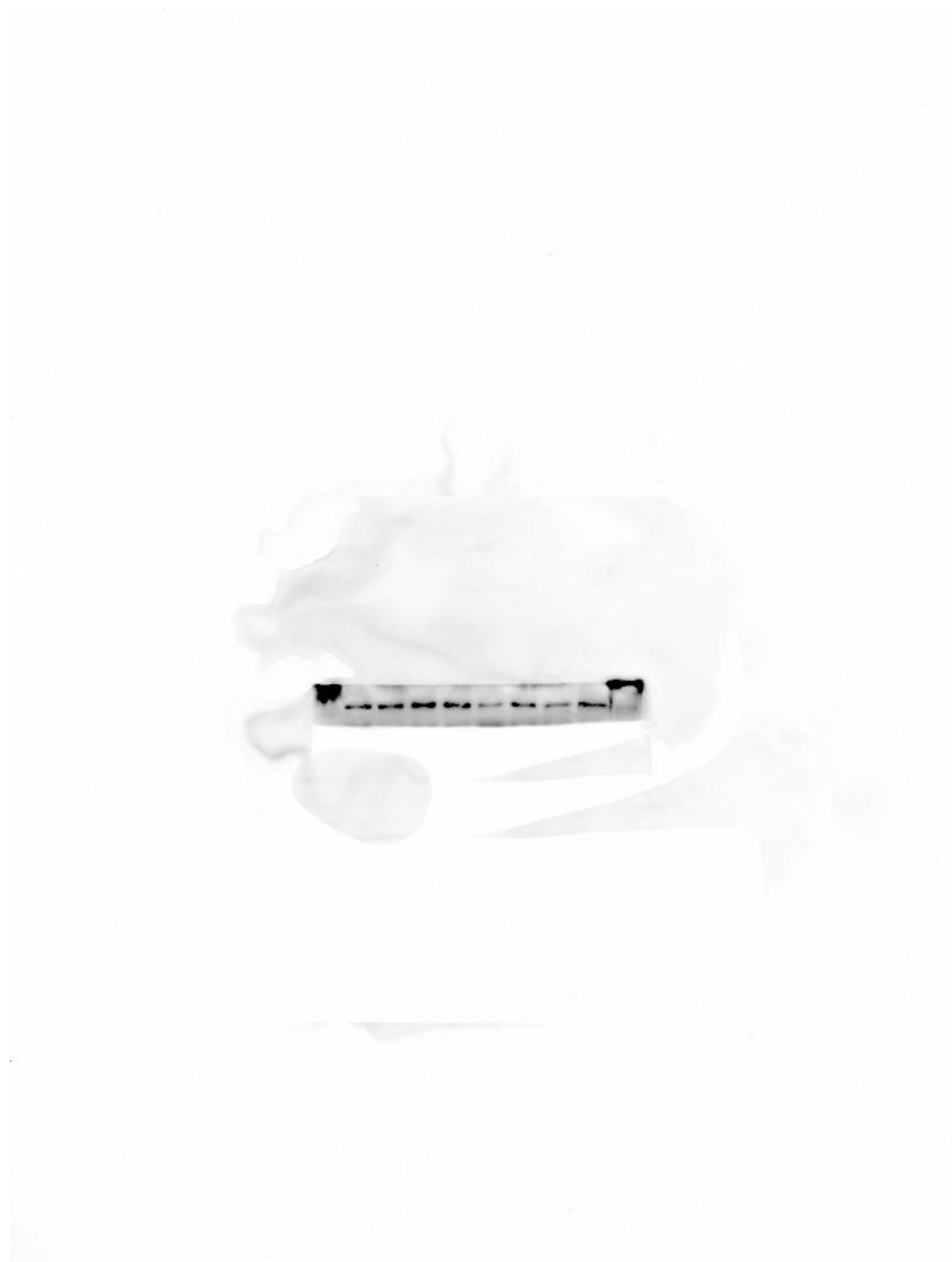

Supplement: Supplementary file 6 — Fig 7-Single Original Western blotting images [file 41419_2023_5623_MOESM6_ESM.zip › Fig 7-Single Original Western blotting images -/Figure 7B/01 Lepr (Fig 7B).pdf]

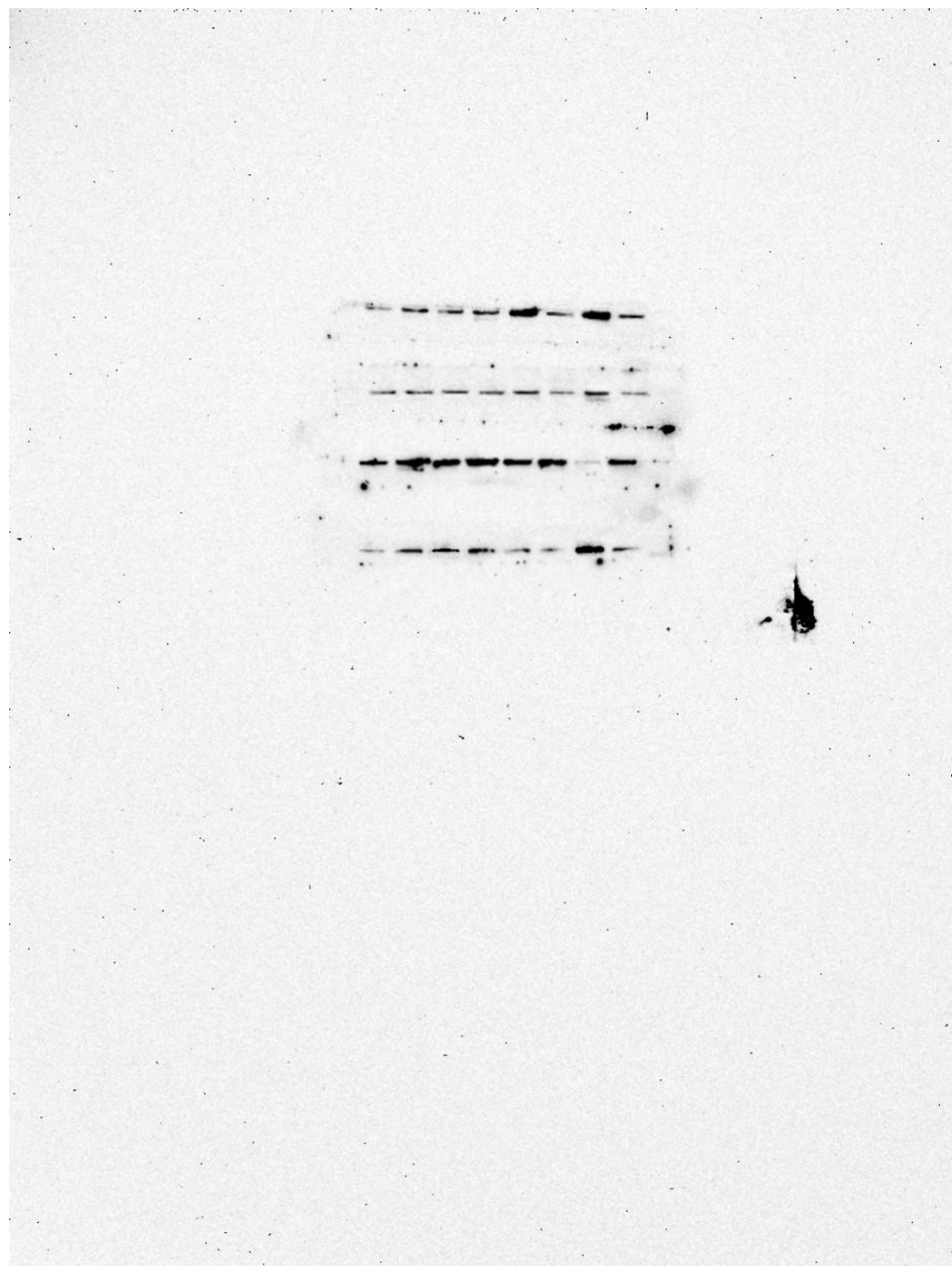

Supplement: Supplementary file 6 — Fig 7-Single Original Western blotting images [file 41419_2023_5623_MOESM6_ESM.zip › Fig 7-Single Original Western blotting images -/Figure 7B/01 pAMPK & 01 CCO & 01 Bcl2 & 01 Cleaved cas3.pdf]

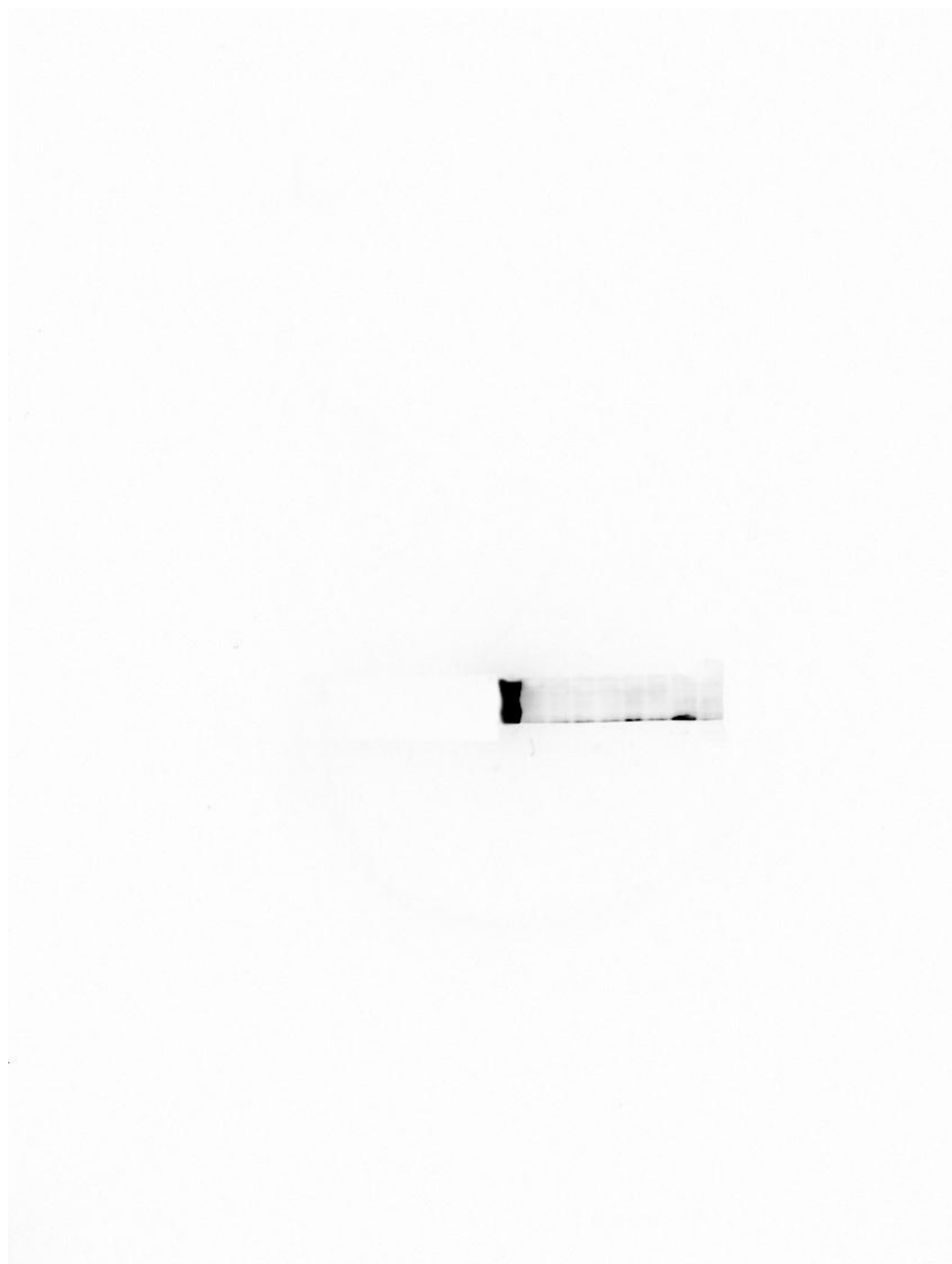

Supplement: Supplementary file 6 — Fig 7-Single Original Western blotting images [file 41419_2023_5623_MOESM6_ESM.zip › Fig 7-Single Original Western blotting images -/Figure 7B/02 Bax (Fig 7B).pdf]

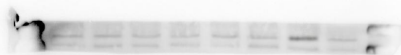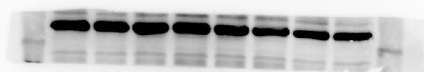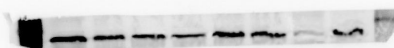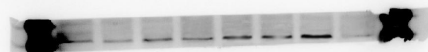

Supplement: Supplementary file 6 — Fig 7-Single Original Western blotting images [file 41419_2023_5623_MOESM6_ESM.zip › Fig 7-Single Original Western blotting images -/Figure 7B/02 Cleaved cas3 & 02 beta-actin & 02 Bcl2 & 02 CCO.pdf]

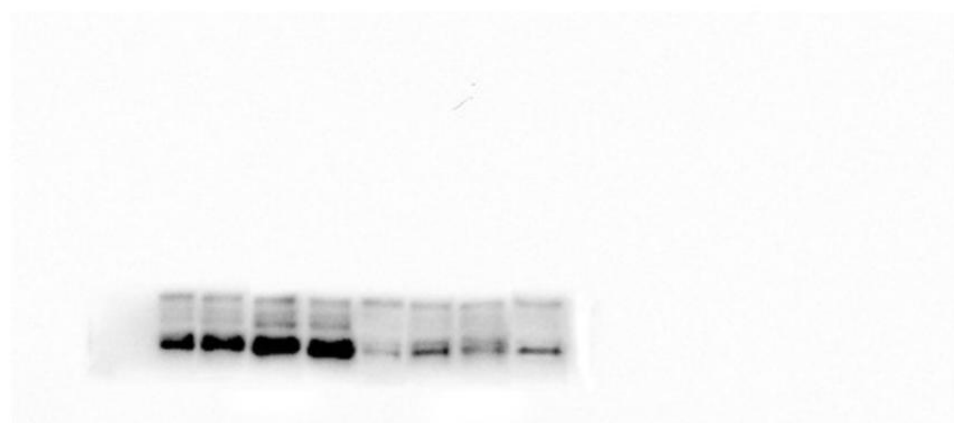

Supplement: Supplementary file 6 — Fig 7-Single Original Western blotting images [file 41419_2023_5623_MOESM6_ESM.zip › Fig 7-Single Original Western blotting images -/Figure 7B/02 Lepr (Fig 7B).pdf]

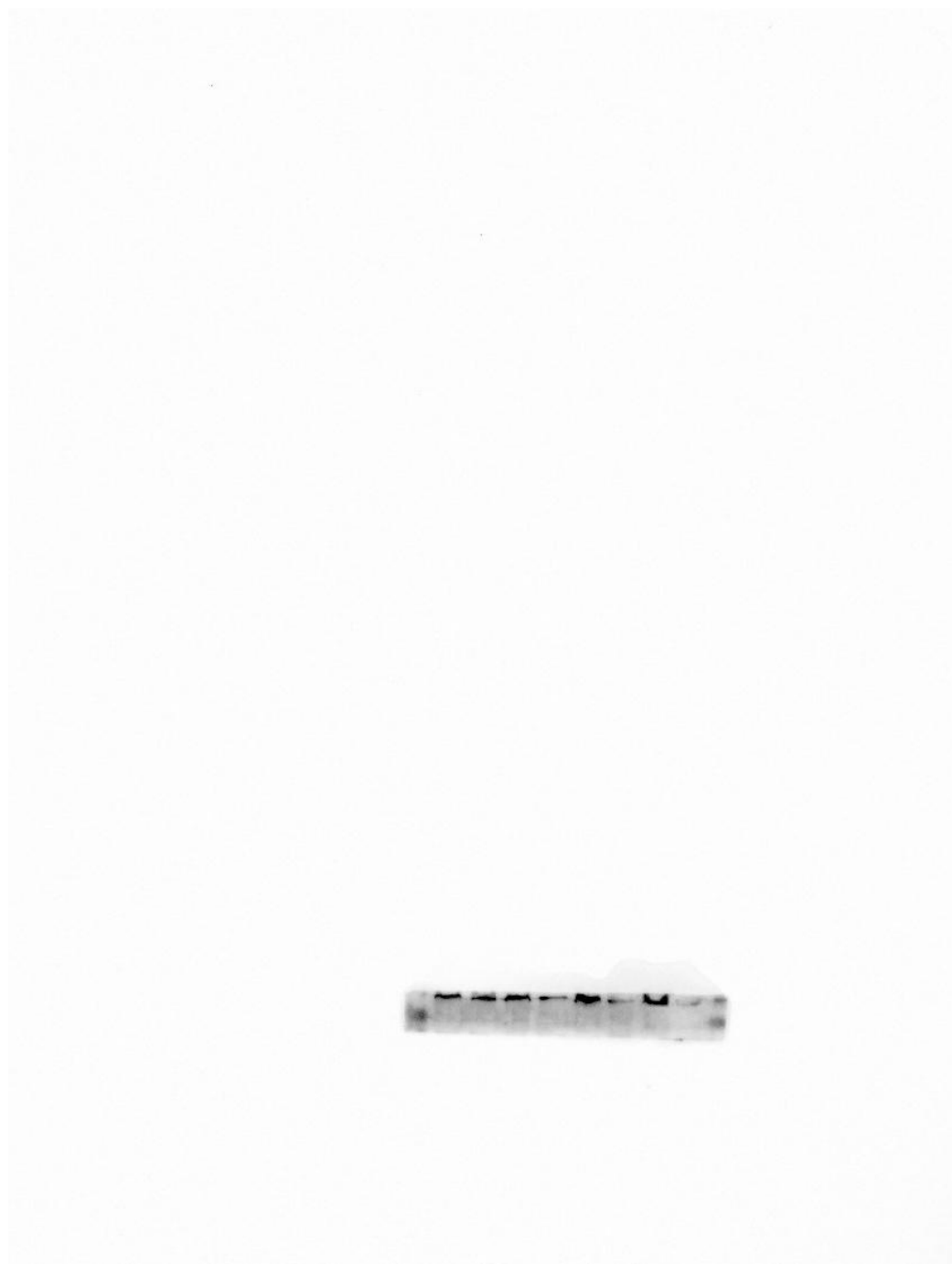

Supplement: Supplementary file 6 — Fig 7-Single Original Western blotting images [file 41419_2023_5623_MOESM6_ESM.zip › Fig 7-Single Original Western blotting images -/Figure 7B/02 pAMPK (Fig 7B).pdf]

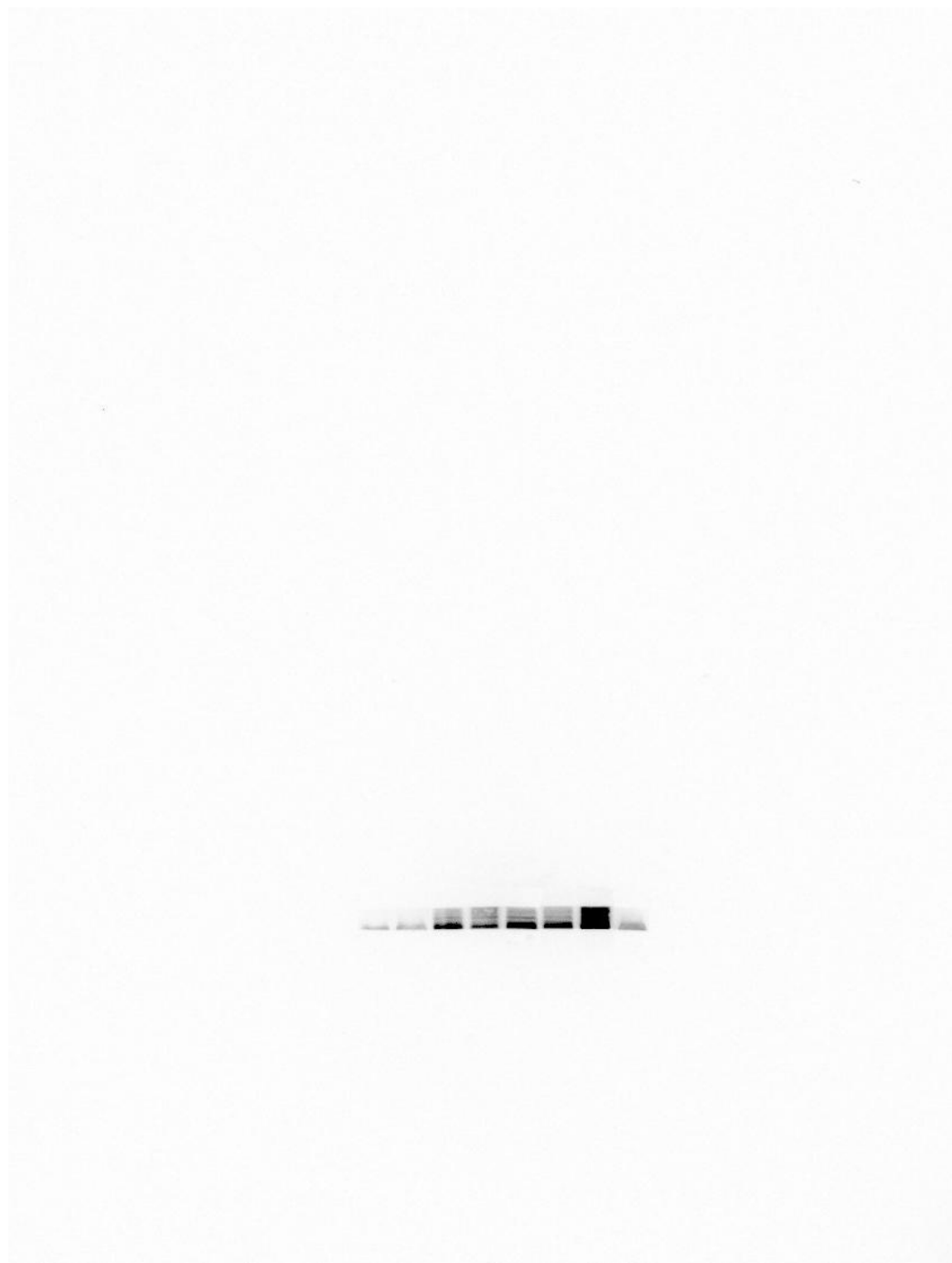

Supplement: Supplementary file 6 — Fig 7-Single Original Western blotting images [file 41419_2023_5623_MOESM6_ESM.zip › Fig 7-Single Original Western blotting images -/Figure 7B/03 Bax (Fig 7B).pdf]

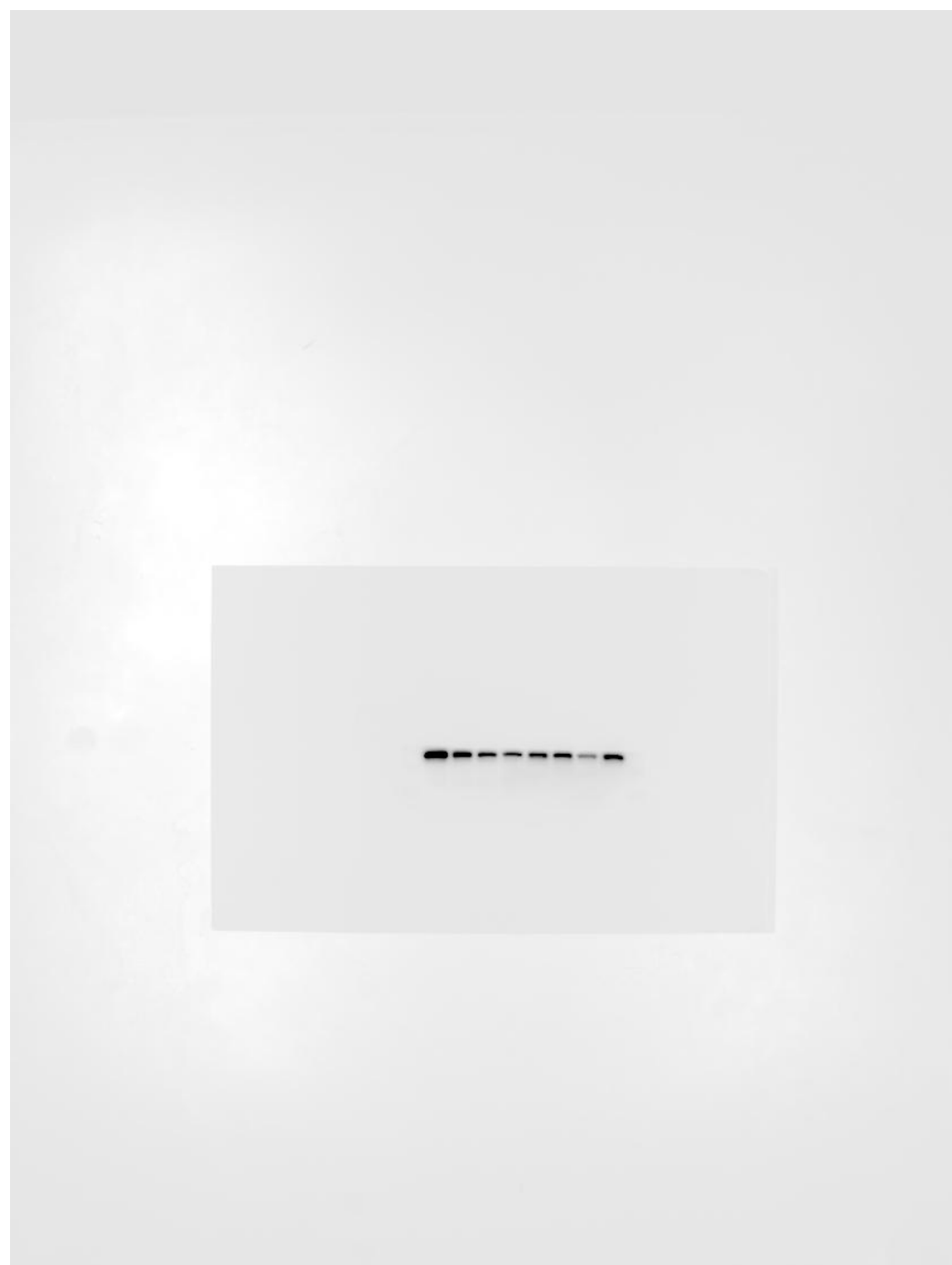

Supplement: Supplementary file 6 — Fig 7-Single Original Western blotting images [file 41419_2023_5623_MOESM6_ESM.zip › Fig 7-Single Original Western blotting images -/Figure 7B/03 Bcl2 (Fig 7B).pdf]

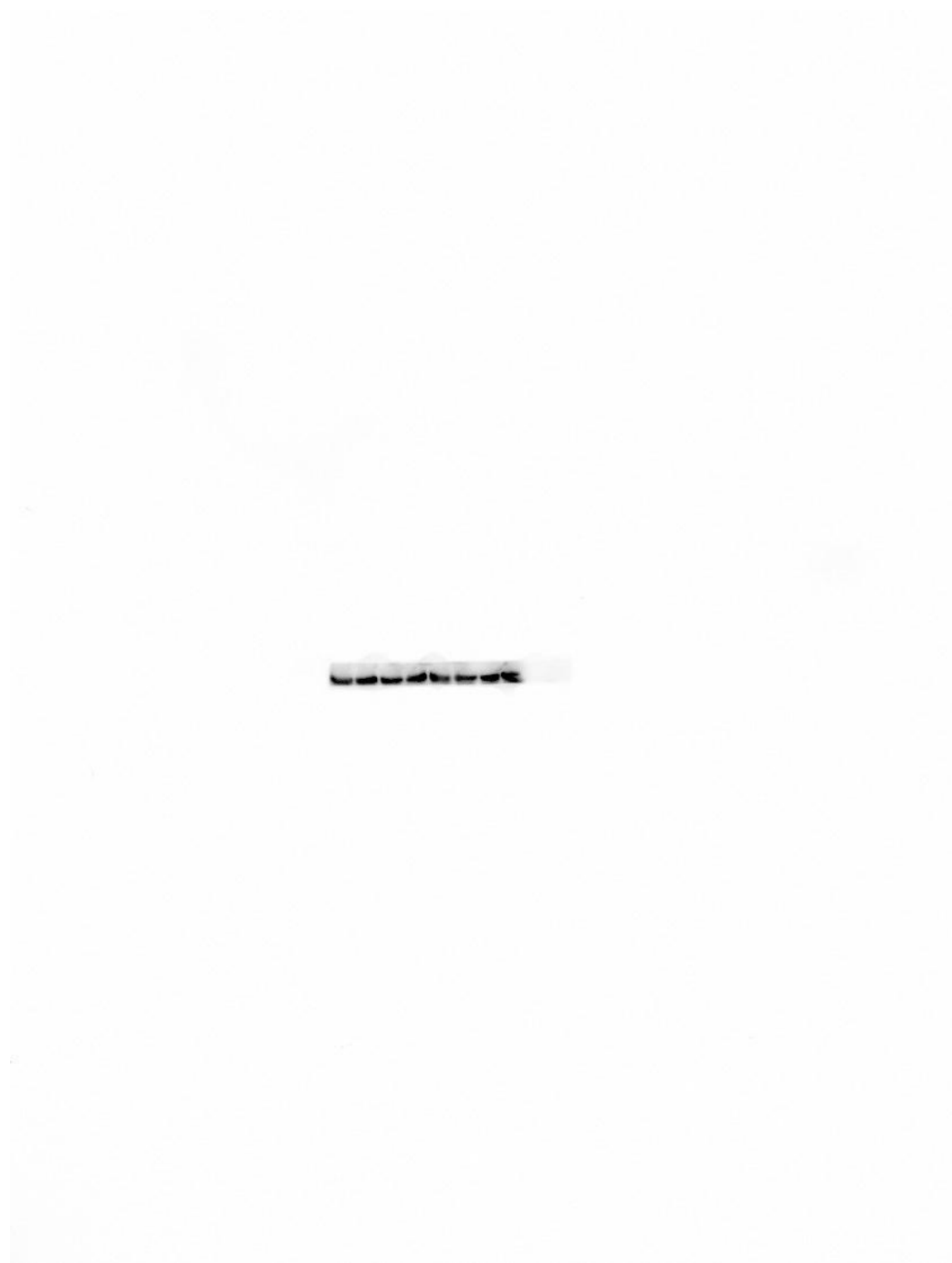

Supplement: Supplementary file 6 — Fig 7-Single Original Western blotting images [file 41419_2023_5623_MOESM6_ESM.zip › Fig 7-Single Original Western blotting images -/Figure 7B/03 beta-actin.pdf]

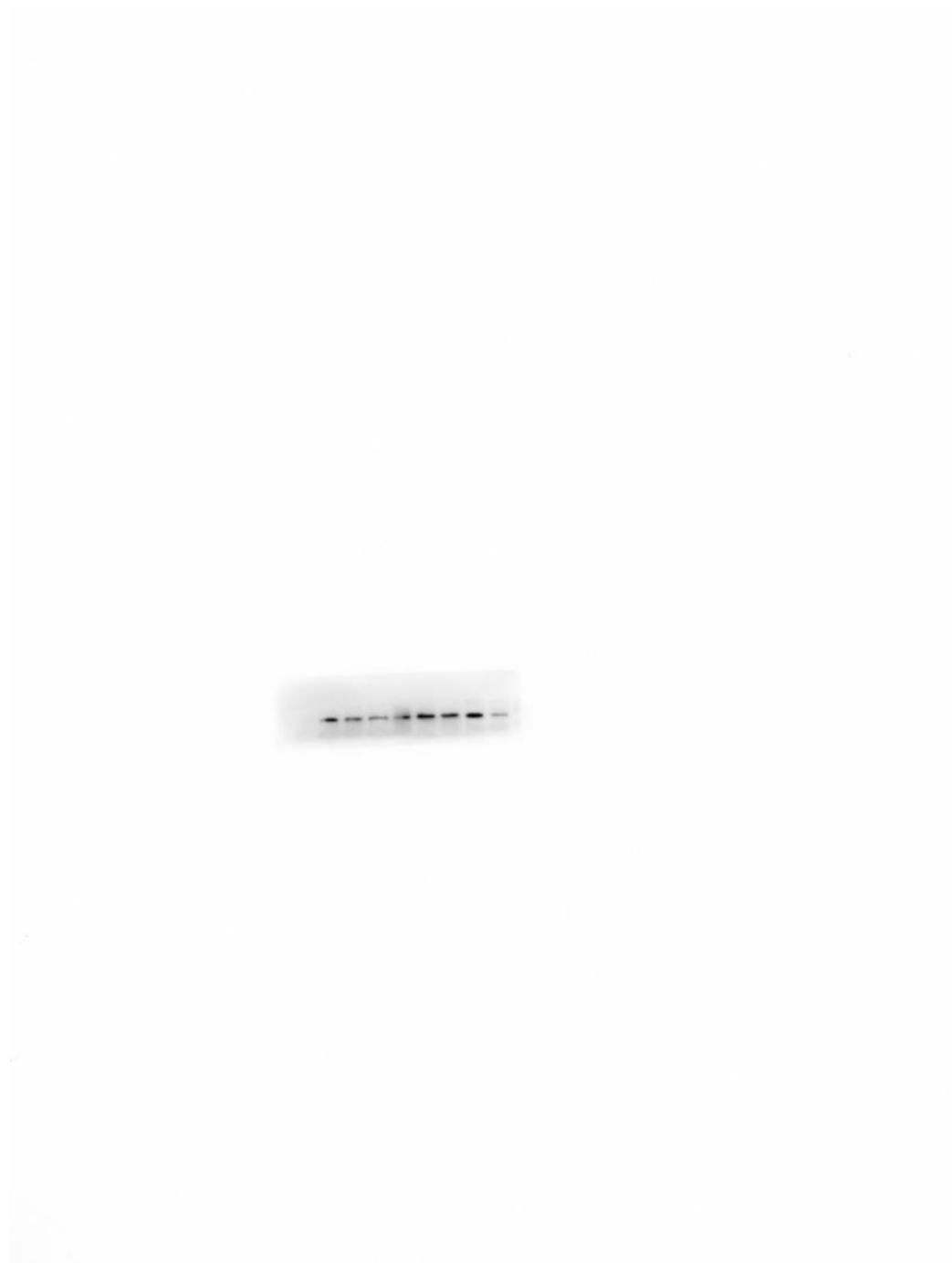

Supplement: Supplementary file 6 — Fig 7-Single Original Western blotting images [file 41419_2023_5623_MOESM6_ESM.zip › Fig 7-Single Original Western blotting images -/Figure 7B/03 CCO (Fig 7B).pdf]

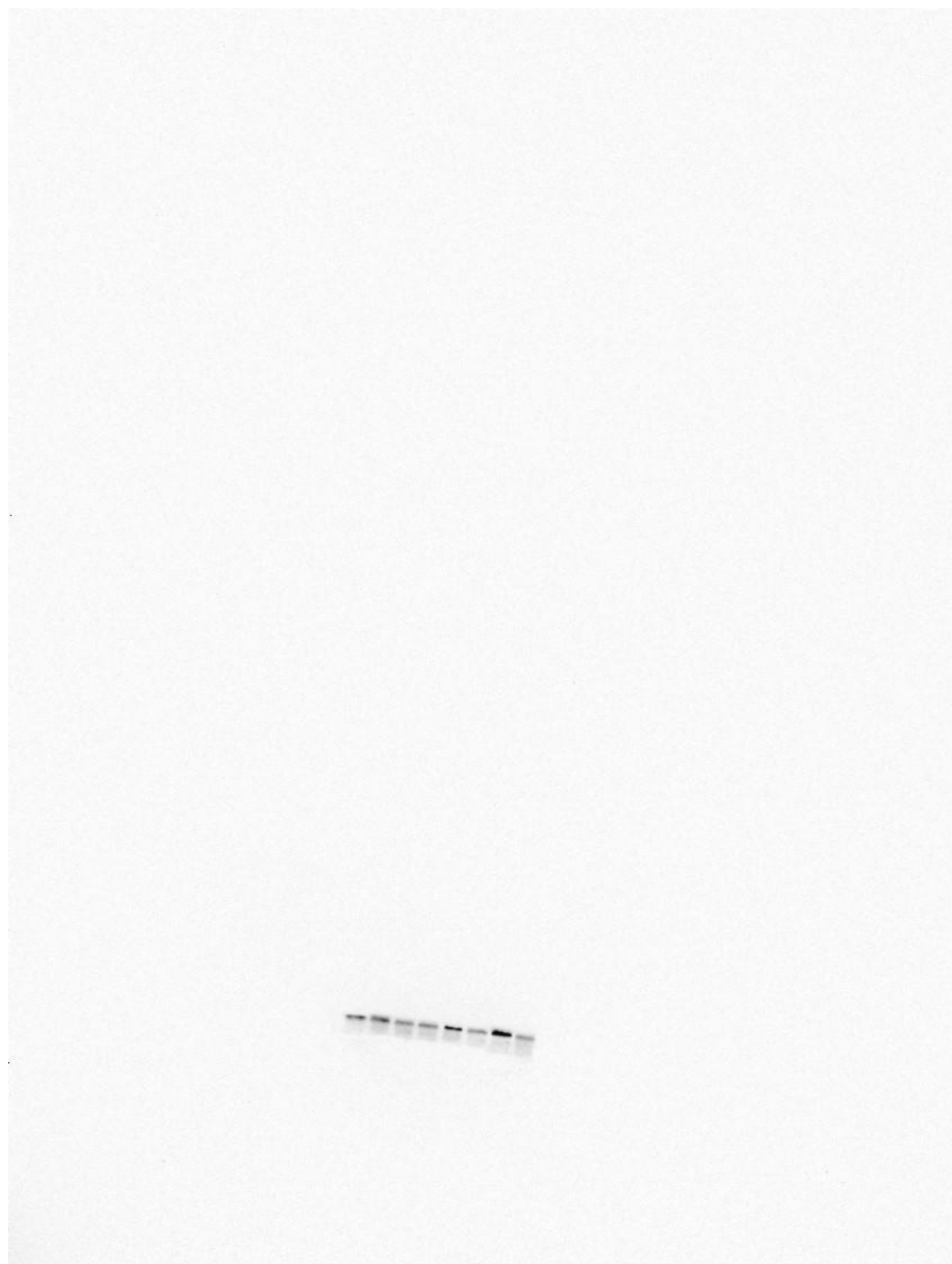

Supplement: Supplementary file 6 — Fig 7-Single Original Western blotting images [file 41419_2023_5623_MOESM6_ESM.zip › Fig 7-Single Original Western blotting images -/Figure 7B/03 Cleaved cas3 (Fig 7B).pdf]

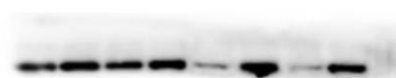

Supplement: Supplementary file 6 — Fig 7-Single Original Western blotting images [file 41419_2023_5623_MOESM6_ESM.zip › Fig 7-Single Original Western blotting images -/Figure 7B/03 Lepr (Fig 7B).pdf]

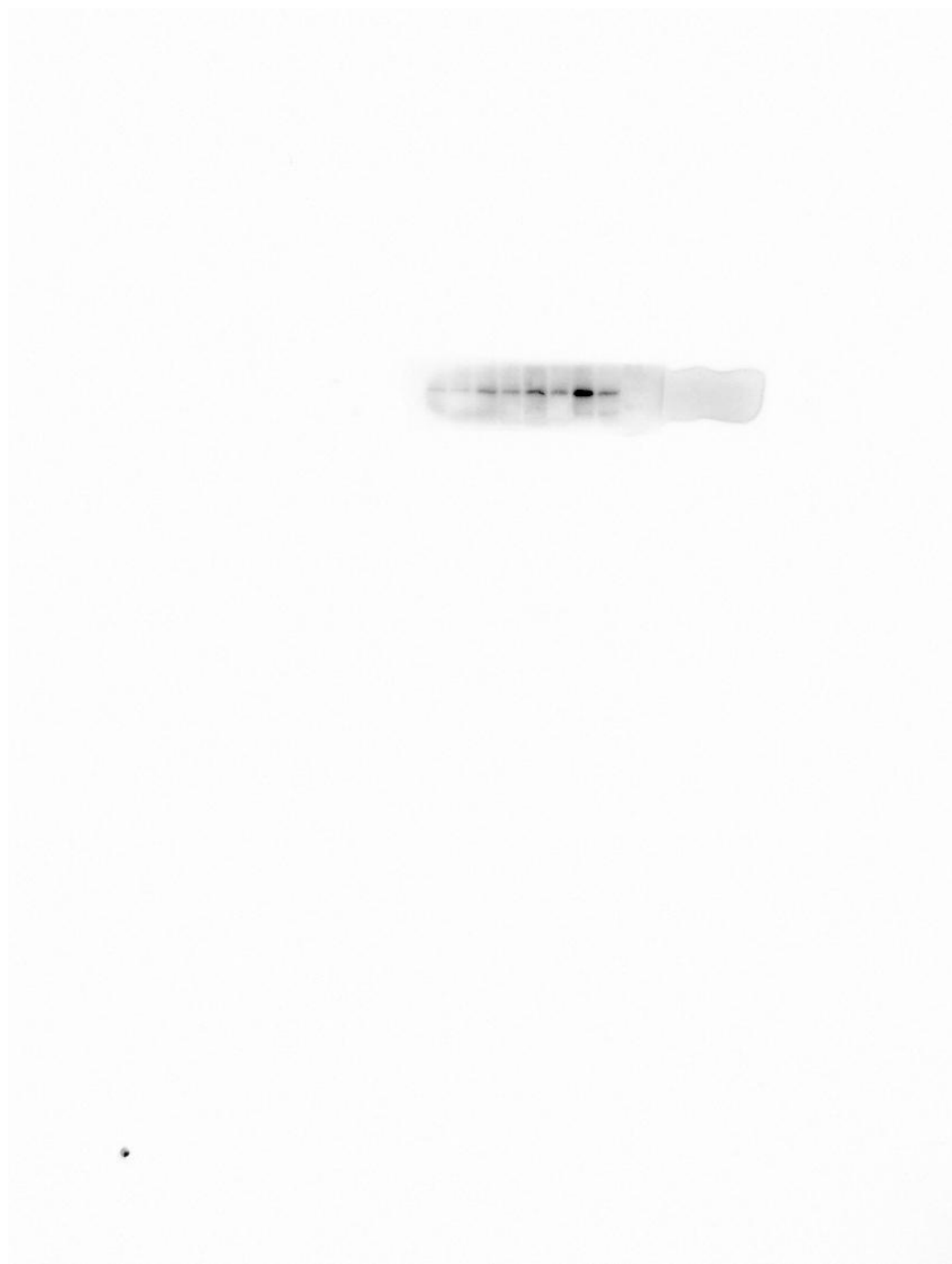

Supplement: Supplementary file 6 — Fig 7-Single Original Western blotting images [file 41419_2023_5623_MOESM6_ESM.zip › Fig 7-Single Original Western blotting images -/Figure 7B/03 pAMPK (Fig 7B).pdf]

IL-1 $\beta$

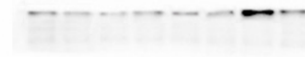

$\beta$ -actin

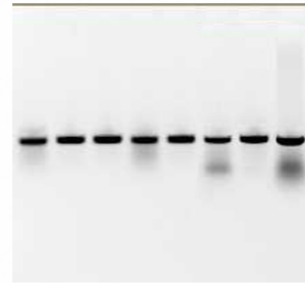

Caspase-11

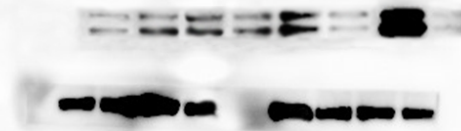

GSDMD-N

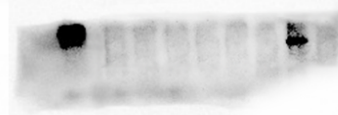

cleaved  
Caspase-9

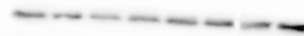

GSDMD

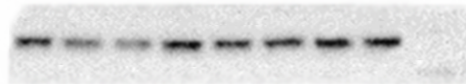

cleaved  
Caspase-8

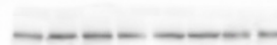

Supplement: Supplementary file 7 — Fig 8-Single Original Western blotting images [file 41419_2023_5623_MOESM7_ESM.zip › Fig 8-Single Original Western blotting images -/Fig 08 (original film) 01.pdf]

cleaved  
Caspase-1

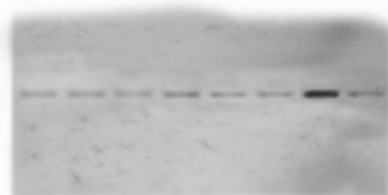

IL-18

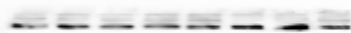

Caspase-5

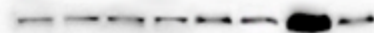

Supplement: Supplementary file 7 — Fig 8-Single Original Western blotting images [file 41419_2023_5623_MOESM7_ESM.zip › Fig 8-Single Original Western blotting images -/Fig 08 (original film) 02.pdf]

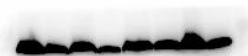

Supplement: Supplementary file 7 — Fig 8-Single Original Western blotting images [file 41419_2023_5623_MOESM7_ESM.zip › Fig 8-Single Original Western blotting images -/Figure 8C/01 beta-actin (Fig 8C).pdf]

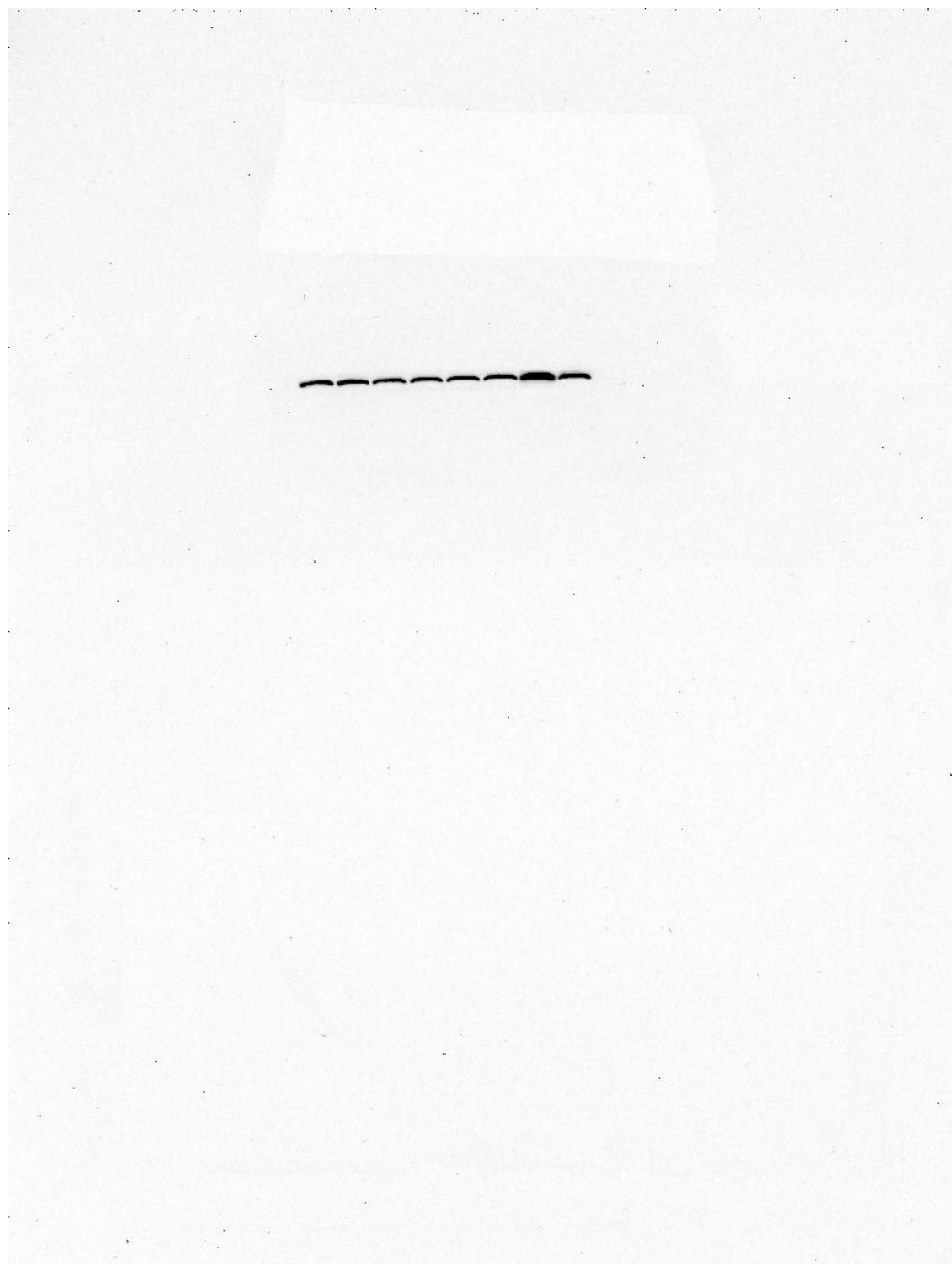

Supplement: Supplementary file 7 — Fig 8-Single Original Western blotting images [file 41419_2023_5623_MOESM7_ESM.zip › Fig 8-Single Original Western blotting images -/Figure 8C/01 Cas5 (Fig 8C).pdf]

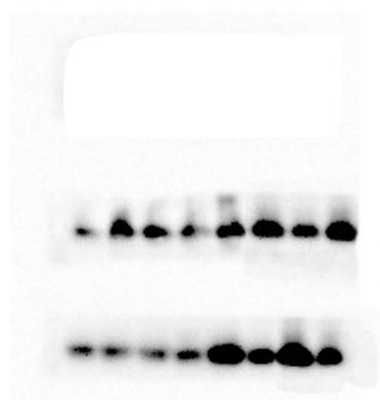

Supplement: Supplementary file 7 — Fig 8-Single Original Western blotting images [file 41419_2023_5623_MOESM7_ESM.zip › Fig 8-Single Original Western blotting images -/Figure 8C/01 Cas8 & 01 Cas9 (Fig 8C).pdf]

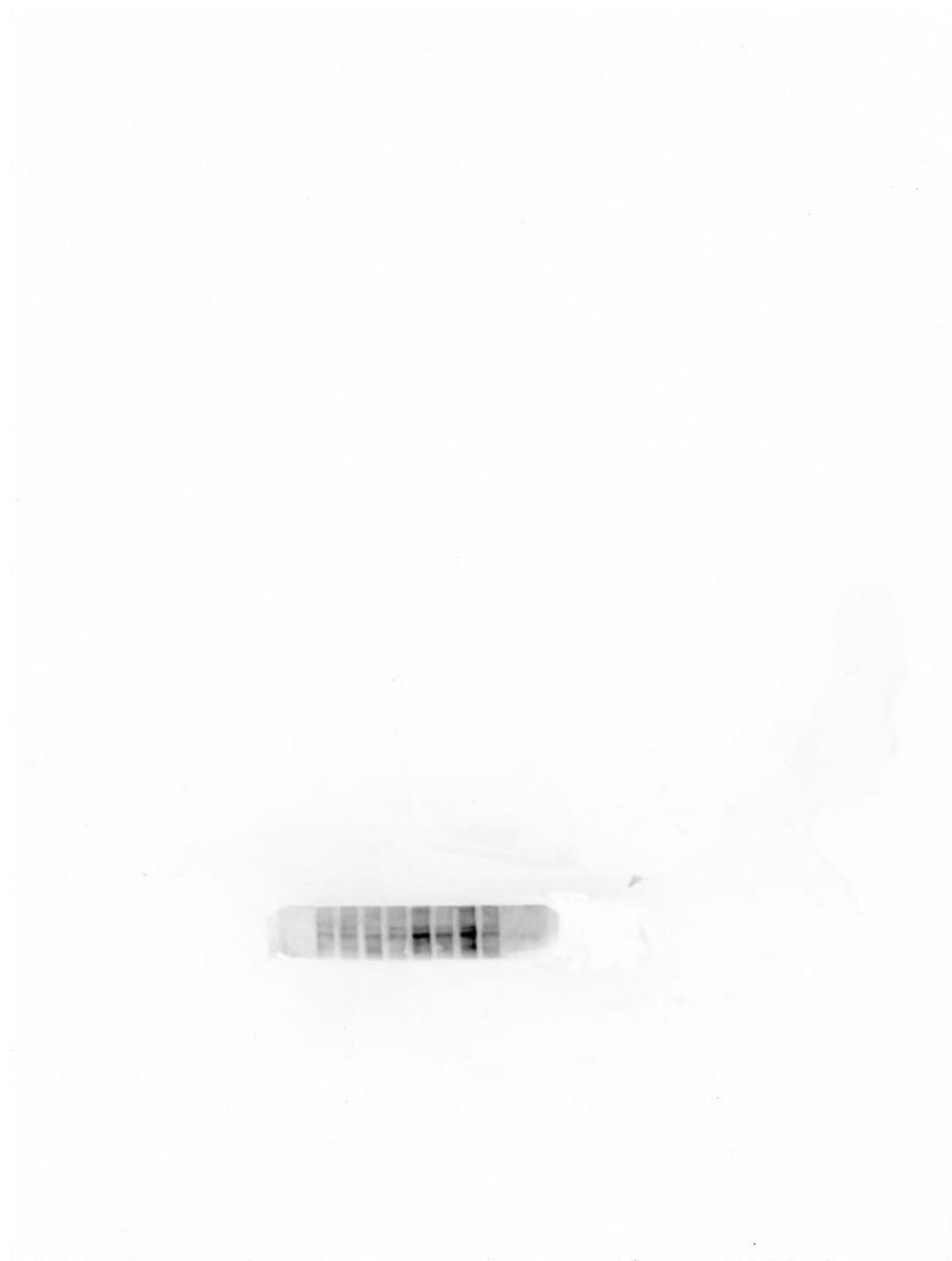

Supplement: Supplementary file 7 — Fig 8-Single Original Western blotting images [file 41419_2023_5623_MOESM7_ESM.zip › Fig 8-Single Original Western blotting images -/Figure 8C/01 Caspase-11 (Fig 8C).pdf]

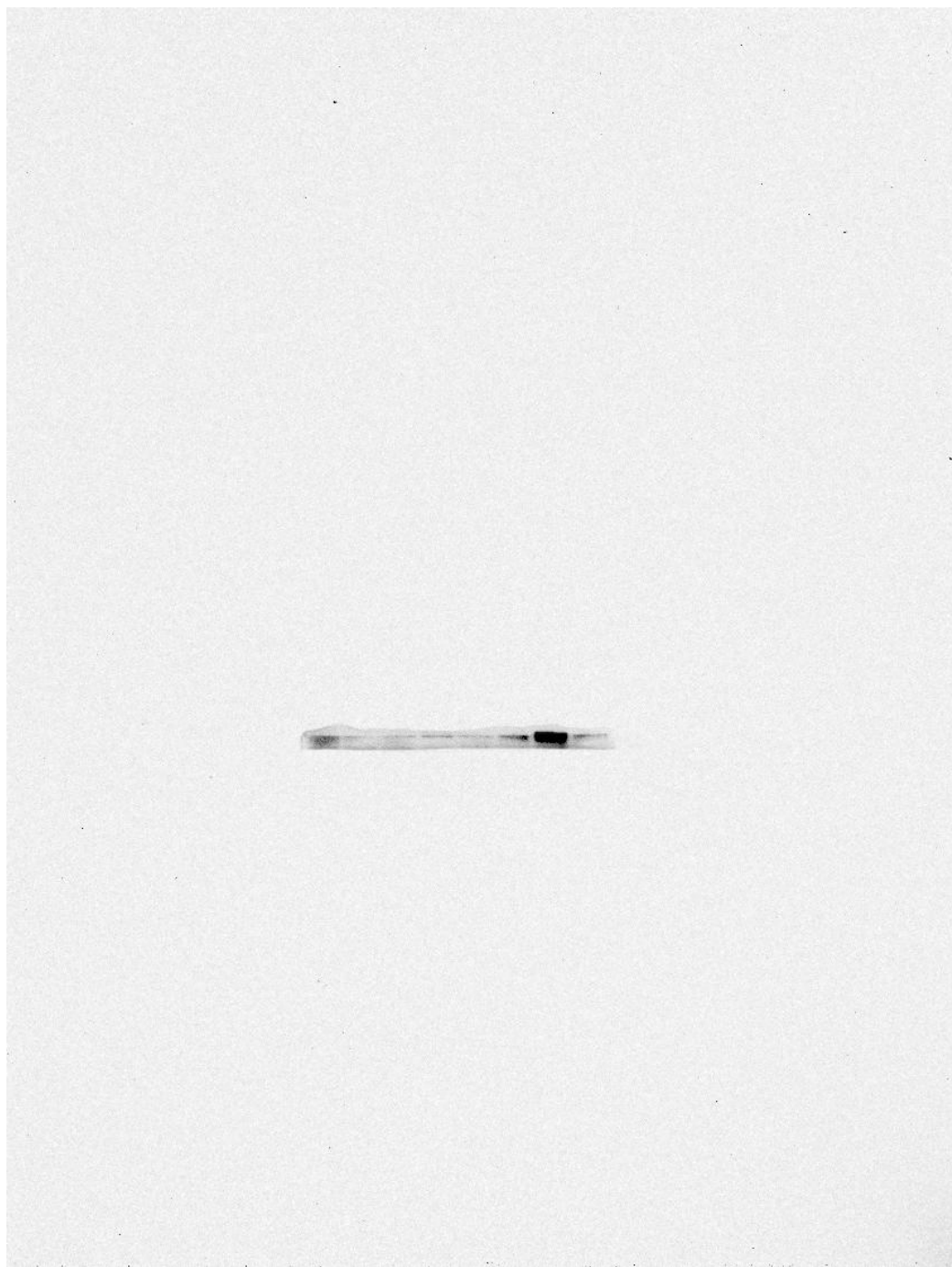

Supplement: Supplementary file 7 — Fig 8-Single Original Western blotting images [file 41419_2023_5623_MOESM7_ESM.zip › Fig 8-Single Original Western blotting images -/Figure 8C/01 Cleaved cas1 (Fig 8C).pdf]

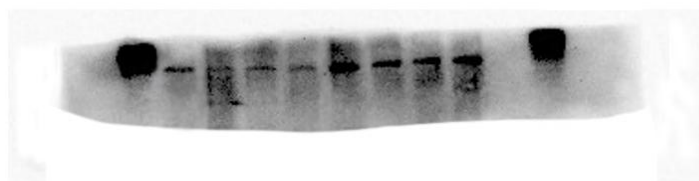

Supplement: Supplementary file 7 — Fig 8-Single Original Western blotting images [file 41419_2023_5623_MOESM7_ESM.zip › Fig 8-Single Original Western blotting images -/Figure 8C/01 GSDMD (Fig 8C).pdf]

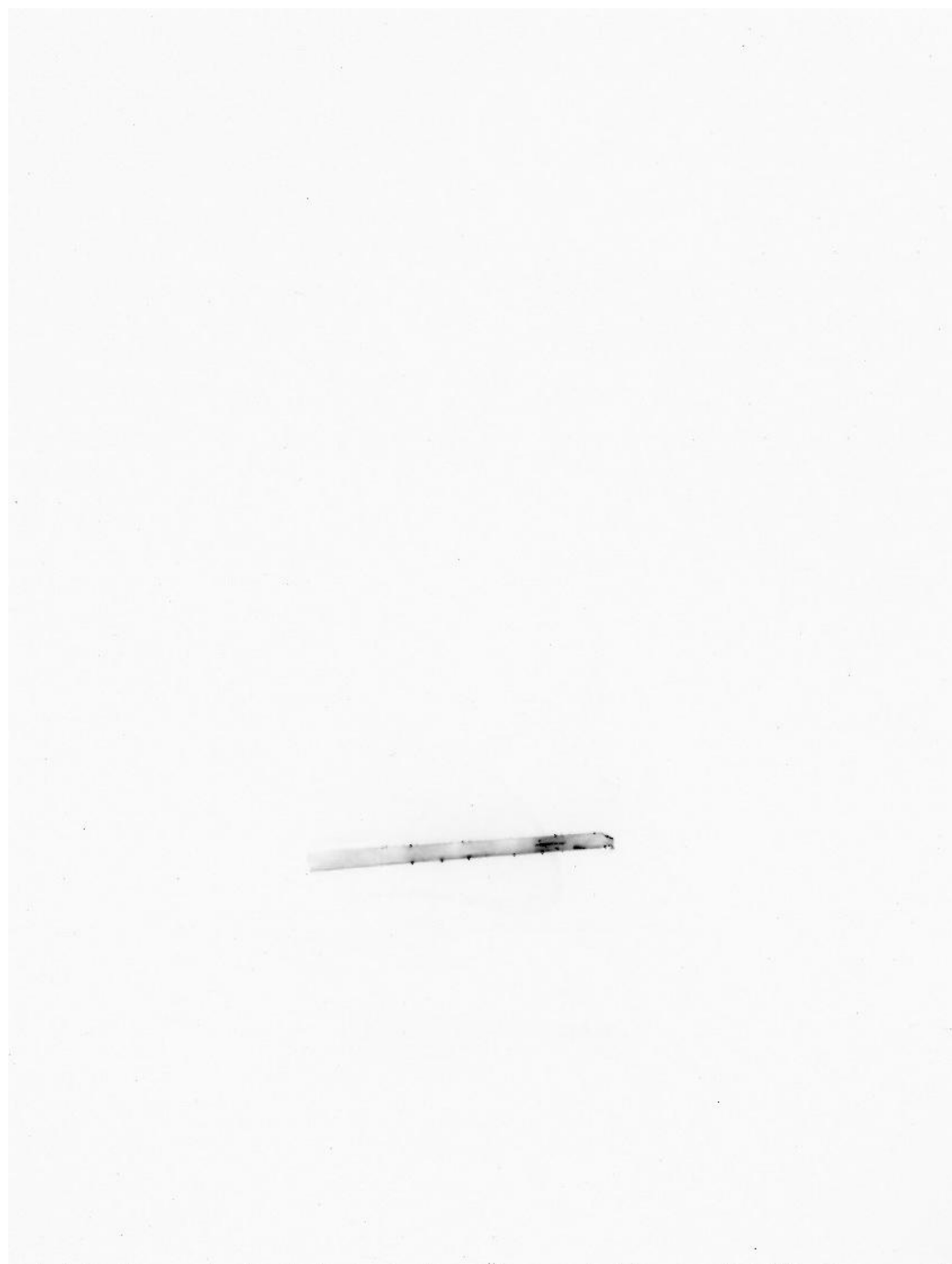

Supplement: Supplementary file 7 — Fig 8-Single Original Western blotting images [file 41419_2023_5623_MOESM7_ESM.zip › Fig 8-Single Original Western blotting images -/Figure 8C/01 GSDME-N (Fig 8C).pdf]

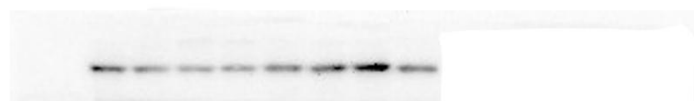

Supplement: Supplementary file 7 — Fig 8-Single Original Western blotting images [file 41419_2023_5623_MOESM7_ESM.zip › Fig 8-Single Original Western blotting images -/Figure 8C/01 IL-18 (Fig 8C).pdf]

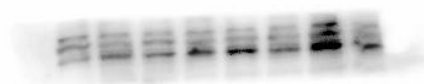

Supplement: Supplementary file 7 — Fig 8-Single Original Western blotting images [file 41419_2023_5623_MOESM7_ESM.zip › Fig 8-Single Original Western blotting images -/Figure 8C/01 IL-1beta (Fig 8C).pdf]

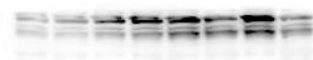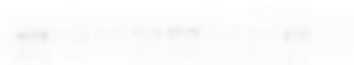

Supplement: Supplementary file 7 — Fig 8-Single Original Western blotting images [file 41419_2023_5623_MOESM7_ESM.zip › Fig 8-Single Original Western blotting images -/Figure 8C/02 Cas11 (Fig 8C).pdf]

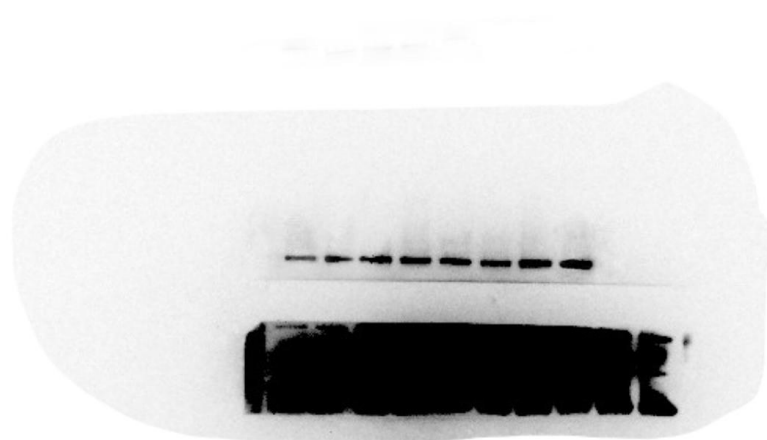

Supplement: Supplementary file 7 — Fig 8-Single Original Western blotting images [file 41419_2023_5623_MOESM7_ESM.zip › Fig 8-Single Original Western blotting images -/Figure 8C/02 Cas9 (Fig 8C).pdf]

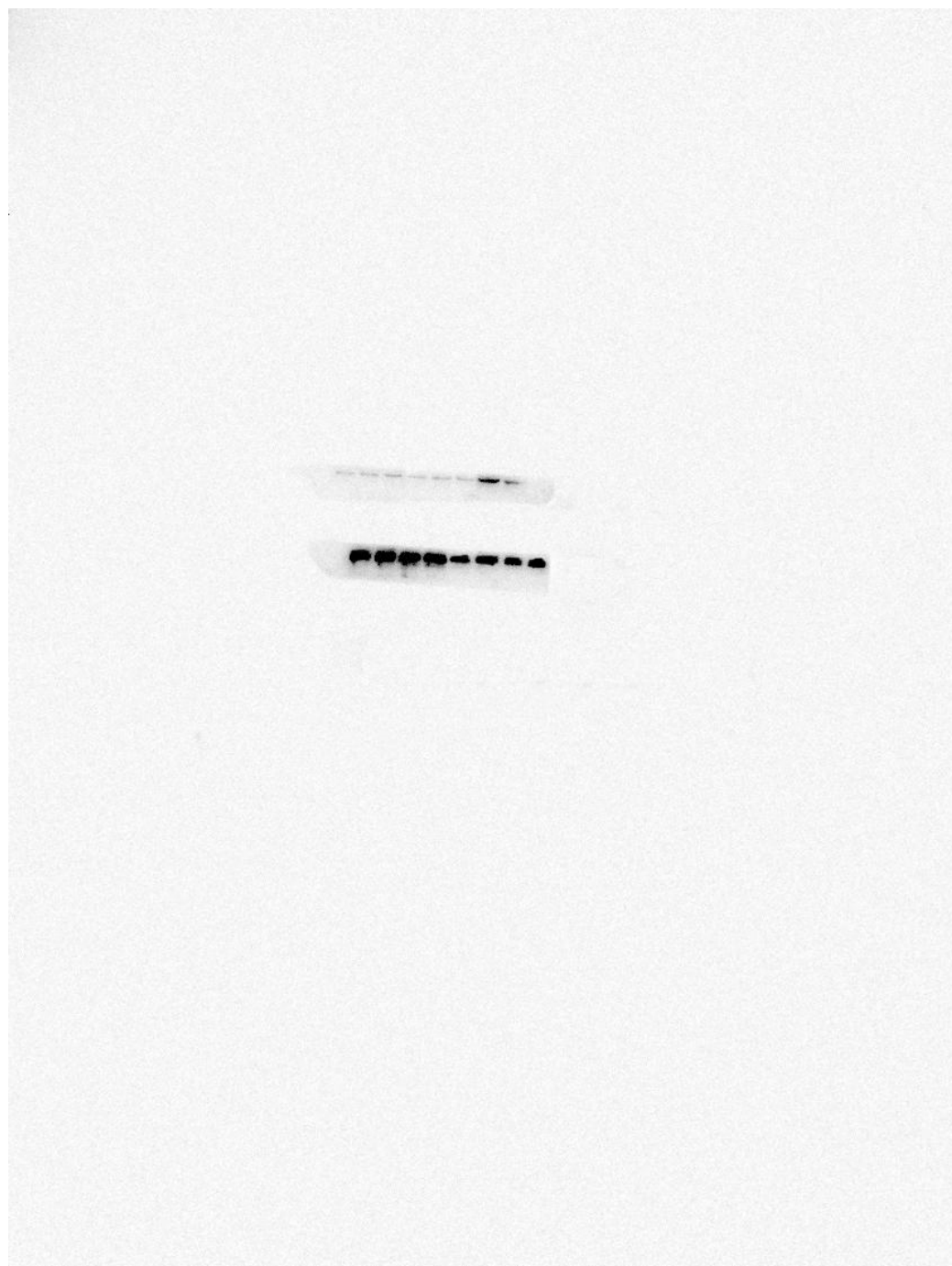

Supplement: Supplementary file 7 — Fig 8-Single Original Western blotting images [file 41419_2023_5623_MOESM7_ESM.zip › Fig 8-Single Original Western blotting images -/Figure 8C/02 Cleaved cas1 & 02 GSDMD (Fig 8C).pdf]

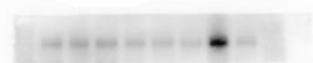

Supplement: Supplementary file 7 — Fig 8-Single Original Western blotting images [file 41419_2023_5623_MOESM7_ESM.zip › Fig 8-Single Original Western blotting images -/Figure 8C/02 GSDME-N (Fig 8C).pdf]

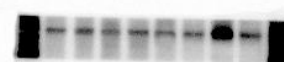

Supplement: Supplementary file 7 — Fig 8-Single Original Western blotting images [file 41419_2023_5623_MOESM7_ESM.zip › Fig 8-Single Original Western blotting images -/Figure 8C/02 IL-18 (Fig 8C).pdf]

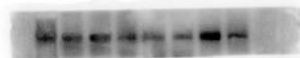

Supplement: Supplementary file 7 — Fig 8-Single Original Western blotting images [file 41419_2023_5623_MOESM7_ESM.zip › Fig 8-Single Original Western blotting images -/Figure 8C/02 IL-1beta (Fig 8C).pdf]

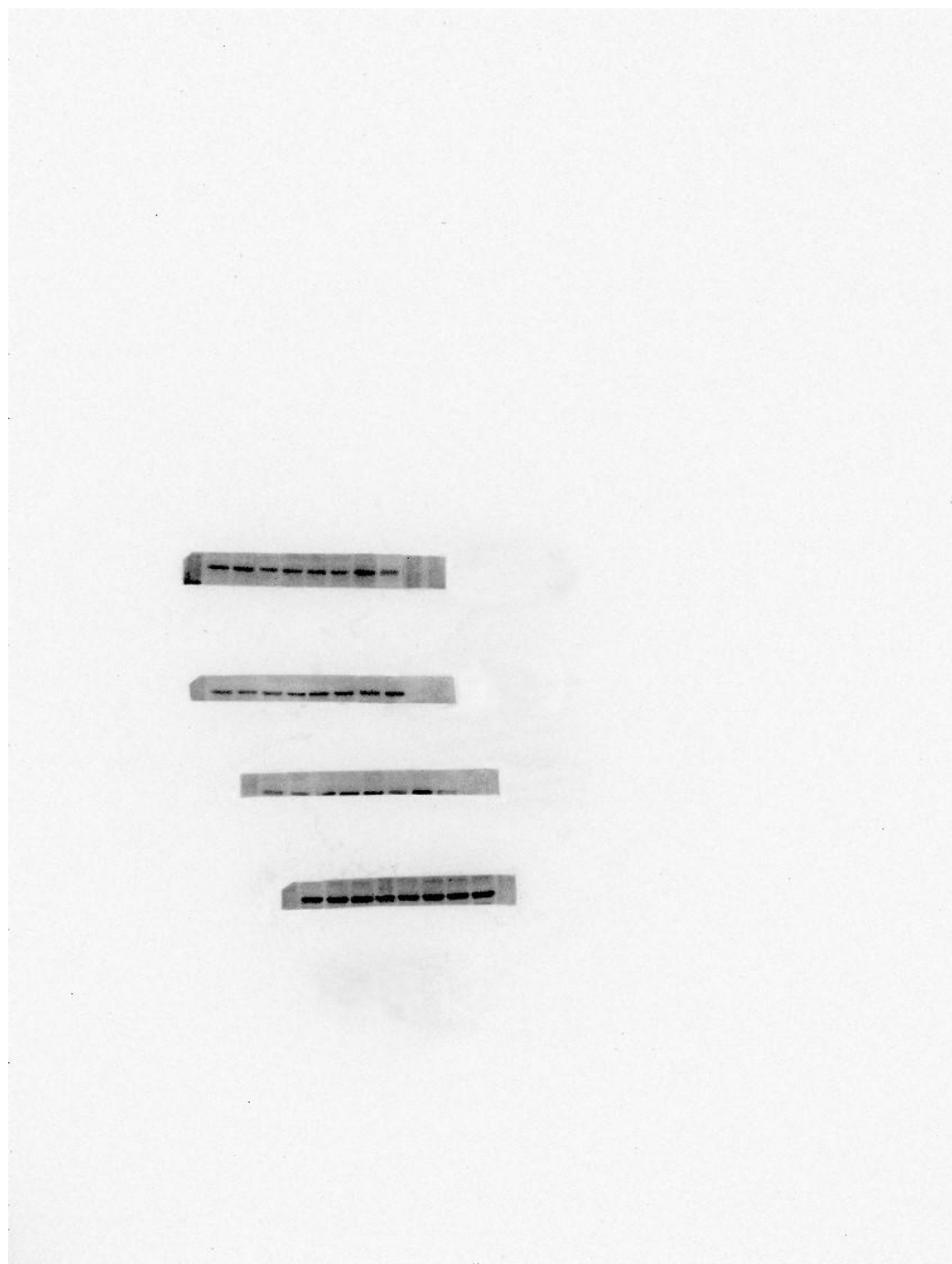

Supplement: Supplementary file 7 — Fig 8-Single Original Western blotting images [file 41419_2023_5623_MOESM7_ESM.zip › Fig 8-Single Original Western blotting images -/Figure 8C/03 Cas5 & 03 GSDMD & 03 IL-18 & 03 Cleaved cas9 (Fig 8C).pdf]

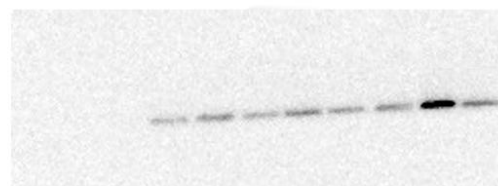

Supplement: Supplementary file 7 — Fig 8-Single Original Western blotting images [file 41419_2023_5623_MOESM7_ESM.zip › Fig 8-Single Original Western blotting images -/Figure 8C/03 Cleaved cas1 (Fig 8C).pdf]

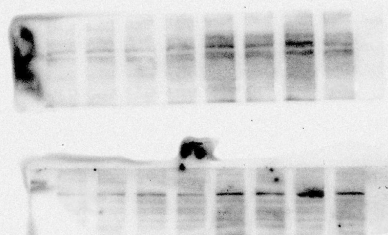

Supplement: Supplementary file 7 — Fig 8-Single Original Western blotting images [file 41419_2023_5623_MOESM7_ESM.zip › Fig 8-Single Original Western blotting images -/Figure 8C/03 Cleaved cas11 & 03 GSDME-N (Fig 8C).pdf]
