# Supplementary material for: Metformin induces pyroptosis in leptin receptor-defective hepatocytes via overactivation of the AMPK axis
Source: Cell Death Dis. 2023 Feb 3;14(2):82. doi: 10.1038/s41419-023-05623-4 (PMC9898507; doi:10.1038/s41419-023-05623-4)

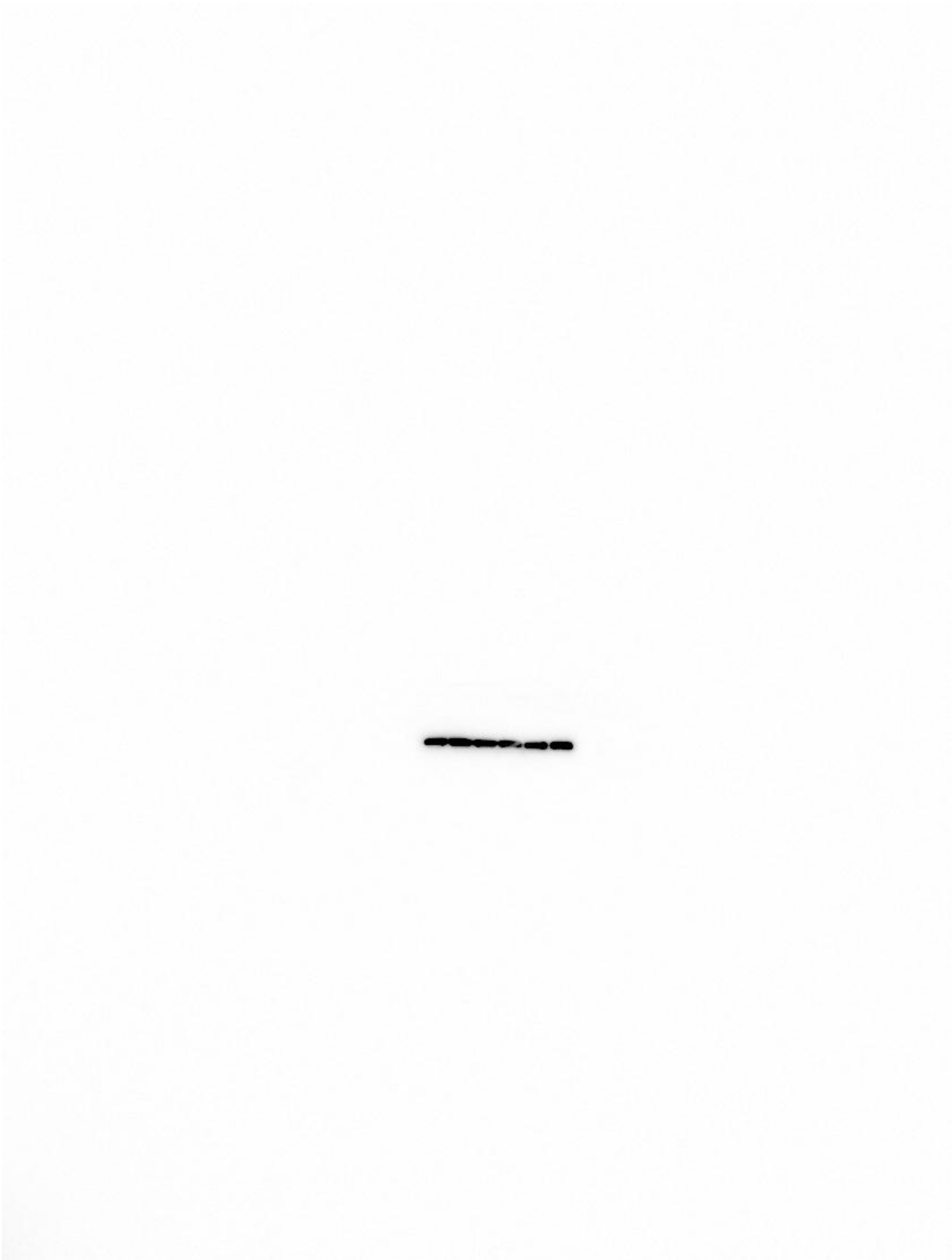

Supplement: Supplementary file 5 — Fig 5-Single Original Western blotting images [file 41419_2023_5623_MOESM5_ESM.zip › Fig 5-Single Original Western blotting images -/Figure 5A/03 beta-actin (Fig 5A 6 lanes).pdf]
