# Supplementary figures and images for: Metformin induces pyroptosis in leptin receptor-defective hepatocytes via overactivation of the AMPK axis (part 2 of 2)
Source: Cell Death Dis. 2023 Feb 3;14(2):82. doi: 10.1038/s41419-023-05623-4 (PMC9898507; doi:10.1038/s41419-023-05623-4)

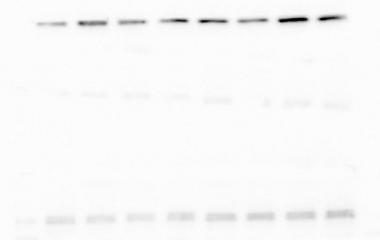

Supplement: Supplementary file 7 — Fig 8-Single Original Western blotting images [file 41419_2023_5623_MOESM7_ESM.zip › Fig 8-Single Original Western blotting images -/Figure 8C/03 IL-1 beta & 03 Cas8 (Fig 8C).pdf]

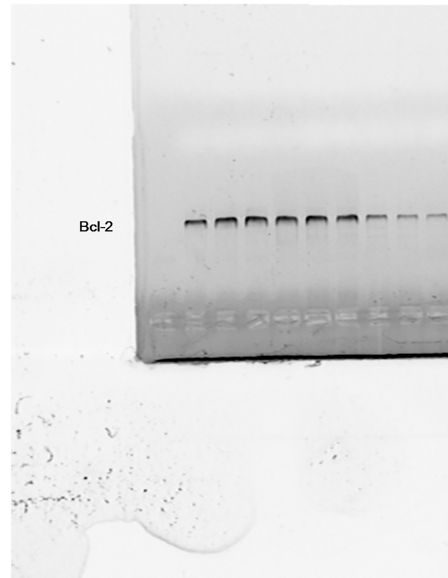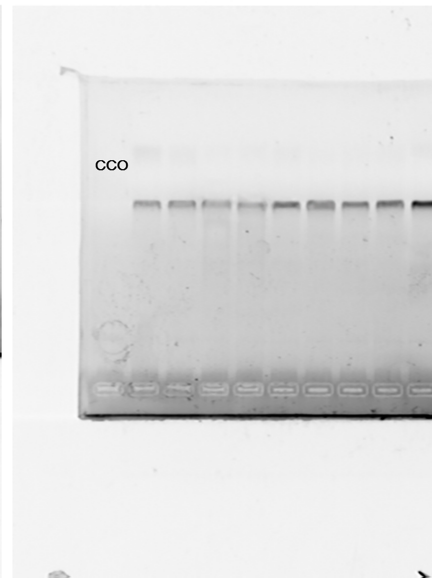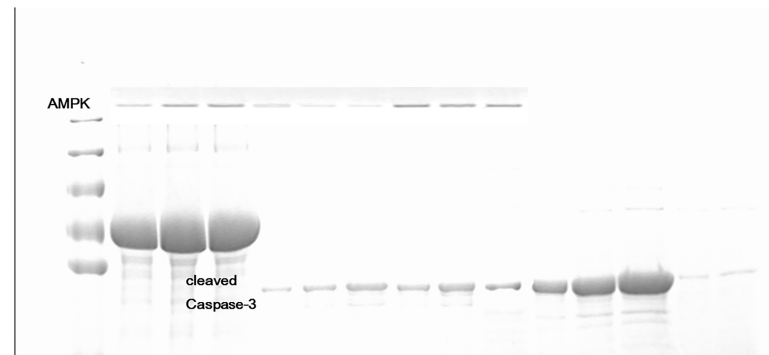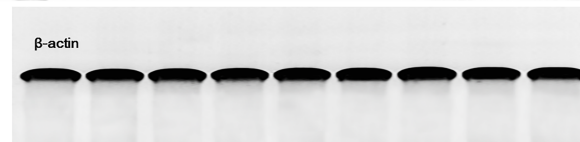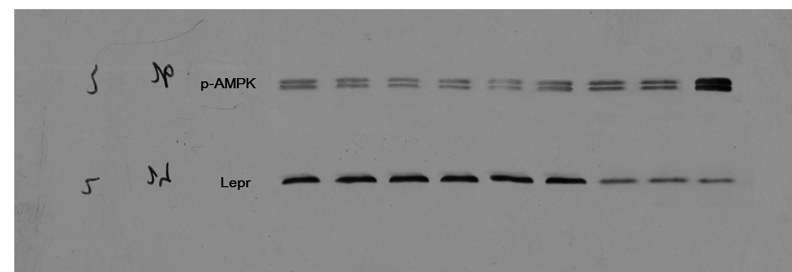

Supplement: Supplementary file 8 — Supplementary material 1-Fig 2A [file 41419_2023_5623_MOESM8_ESM.pdf]

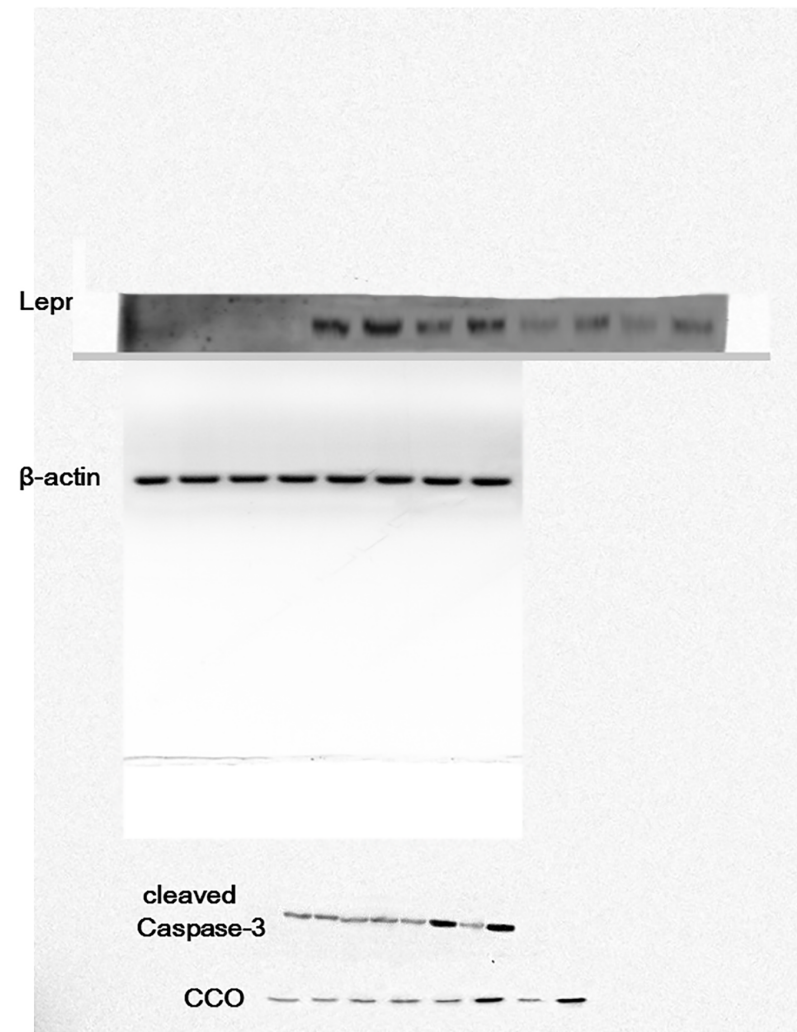

Supplement: Supplementary file 10 — Supplementary material 3-Fig 4A [file 41419_2023_5623_MOESM10_ESM.pdf]

$\beta$ -actin

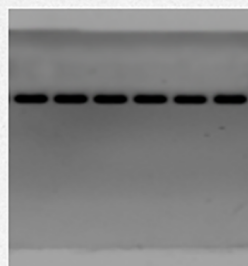

CCO

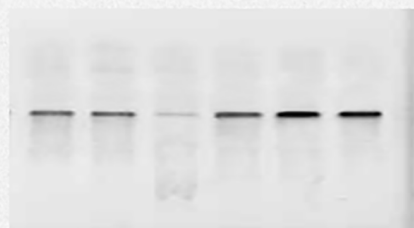

p-AMPK

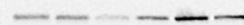

Lepr

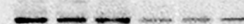

cleaved  
Caspase-3

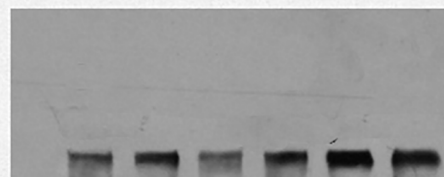

Supplement: Supplementary file 11 — Supplementary material 4-Fig 5A [file 41419_2023_5623_MOESM11_ESM.pdf]

cleaved  
Caspase-3

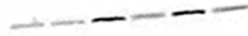

CCO

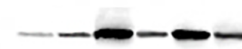

Lepr

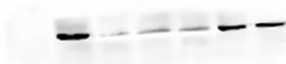

p-AMPK

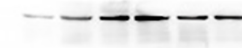

$\beta$ -actin

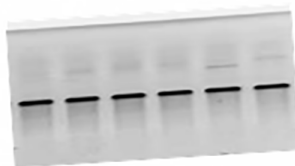

Supplement: Supplementary file 12 — Supplementary material 5-Fig 5C [file 41419_2023_5623_MOESM12_ESM.pdf]

Lepr

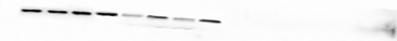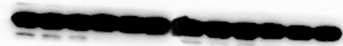

β-actin

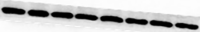

CCO

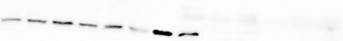

Bax

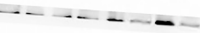

p-AMPK

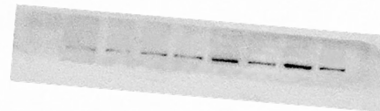

Supplement: Supplementary file 13 — Supplementary material 6-Fig 7-01 [file 41419_2023_5623_MOESM13_ESM.pdf]

Bcl-2

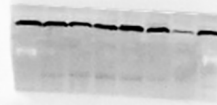

cleaved  
Caspase-3

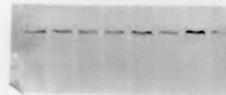

Supplement: Supplementary file 14 — Supplementary material 7-Fig 7-02 [file 41419_2023_5623_MOESM14_ESM.pdf]

IL-1 $\beta$

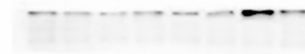

$\beta$ -actin

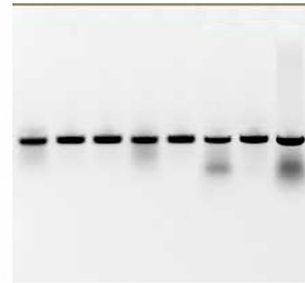

Caspase-11

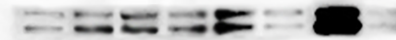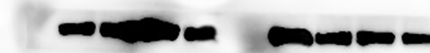

GSDMD-N

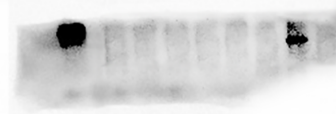

cleaved  
Caspase-9

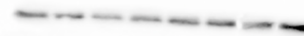

GSDMD

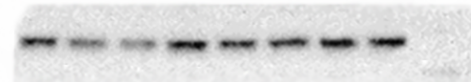

cleaved  
Caspase-8

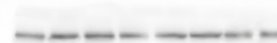

Supplement: Supplementary file 15 — Supplementary material 8-Fig 8-01 [file 41419_2023_5623_MOESM15_ESM.pdf]

cleaved  
Caspase-1

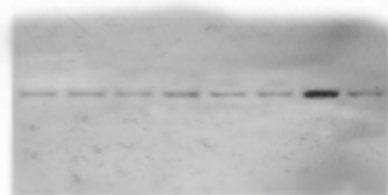

IL-18

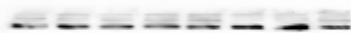

Caspase-5

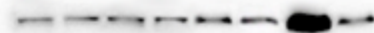

Supplement: Supplementary file 16 — Supplementary material 9-Fig 8-02 [file 41419_2023_5623_MOESM16_ESM.pdf]
