# Supplementary material for: Metformin induces pyroptosis in leptin receptor-defective hepatocytes via overactivation of the AMPK axis
Source: Cell Death Dis. 2023 Feb 3;14(2):82. doi: 10.1038/s41419-023-05623-4 (PMC9898507; doi:10.1038/s41419-023-05623-4)
Supplement: Supplementary file 9 — Supplementary material 2-Fig 2B [file 41419_2023_5623_MOESM9_ESM.pdf]

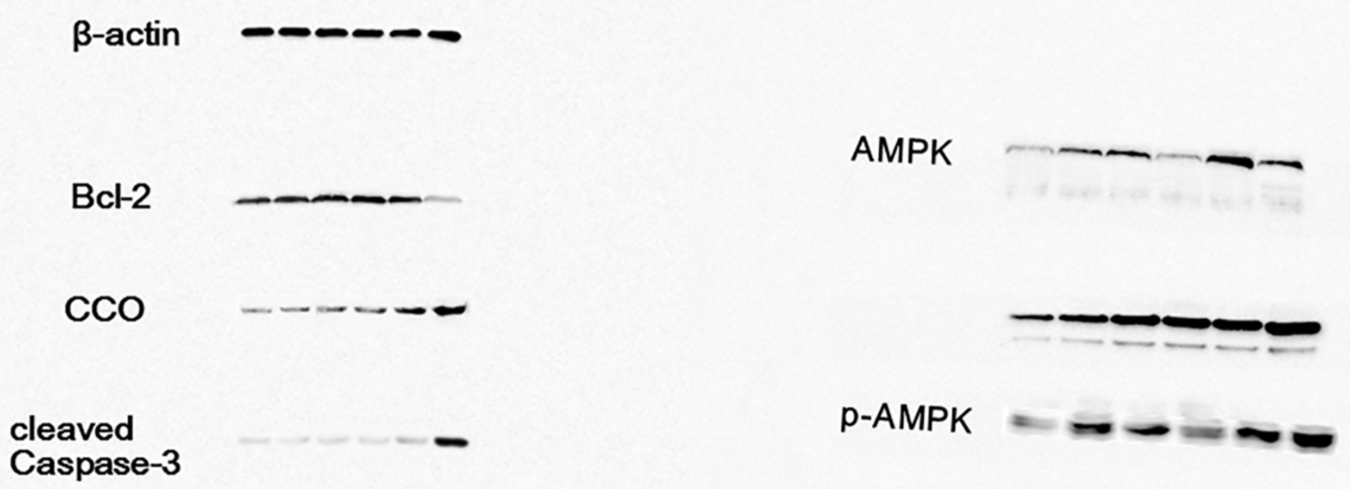
β-actin
Bcl-2
CCO
cleaved
Caspase-3
AMPK
p-AMPK
