# Supplementary material for: Metformin induces pyroptosis in leptin receptor-defective hepatocytes via overactivation of the AMPK axis
Source: Cell Death Dis. 2023 Feb 3;14(2):82. doi: 10.1038/s41419-023-05623-4 (PMC9898507; doi:10.1038/s41419-023-05623-4)
Supplement: Supplementary file 17 — Supplementary material [file 41419_2023_5623_MOESM17_ESM.docx]

**Supplemental Material-Fig 2- Original Western blotting images**

This file contains uncropped original Western blotting images of Figure 2, including AMPK, p-AMPK, Bcl-2, CCO, Cleaved caspase-3, Lepr and β-actin. Images were derived from the original films of at least three replicate Western blotting experiments.

**Supplemental Material-Fig 4- Original Western blotting images**

This file contains uncropped original Western blotting films of Figure 4, including CCO, Cleaved caspase-3, Lepr and β-actin. Images were derived from the original films of at least three replicate Western blotting experiments.

**Supplemental Material-Fig 5- Original Western blotting images**

This file contains uncropped original Western blotting films of Figure 5, including AMPK, p-AMPK, CCO, Cleaved caspase-3, Lepr and β-actin. Images were derived from the original films of at least three replicate Western blotting experiments.

**Supplemental Material-Fig 7- Original Western blotting images**

This file contains uncropped original Western blotting films of Figure 7, including AMPK, p-AMPK, CCO, Cleaved caspase-3, Bax, Lepr and β-actin. Images were derived from the original films of at least three replicate Western blotting experiments.

**Supplemental Material-Fig 8- Original Western blotting images**

This file contains uncropped original Western blotting films of Figure 8, including Caspase-5 Caspase-11, Cleaved caspase-1, GSDMD, GSDME-N, IL-1β, IL-18, Cleaved caspase-9, Cleaved caspase-8, Cleaved caspase-11and β-actin. Images were derived from the original films of at least three replicate Western blotting experiments.

**Supplemental Material-Fig 2A (Original films)**

The original film images of Western blotting in Figure 2A, including Bcl-2, CCO, AMPK, p-AMPK, Cleaved caspase-3, Lepr and β-actin.

**Supplemental Material-Fig 2B (Original films)**

The original film images of Western blotting in Figure 2B, including Bcl-2, CCO, AMPK, p-AMPK, Cleaved caspase-3, Lepr and β-actin.

**Supplemental Material-Fig 4A (Original films)**

The original film images of Western blotting in Figure 4A, including CCO, Cleaved caspase-3, Lepr and β-actin.

**Supplemental Material-Fig 5A (Original films)**

The original film images of Western blotting in Figure 5A, including CCO, p-AMPK, Cleaved caspase-3, Lepr and β-actin.

**Supplemental Material-Fig 5C (Original films)**

The original film images of Western blotting in Figure 5C, including CCO, p-AMPK, Cleaved caspase-3, Lepr and β-actin.

**Supplemental Material-Figure 7 (Original films)01**

The original film images of Western blotting in Figure 7, including Bax, CCO, p-AMPK, Lepr and β-actin.

**Supplemental Material-Figure 7 (Original films) 02**

The original film images of Western blotting in Figure 7, including Bcl-2 and Cleaved caspase-3.

**Supplemental Material-Fig 08 (original films) 01**

The original film images of Western blotting in Figure 8, including IL-1β, Caspase-11, GSDME-N, Cleaved caspase-9, GSDMD, Cleaved caspase-8 and β-actin.

**Supplemental Material-Fig 08 (original films) 02**

The original film images of Western blotting in Figure 8, including Cleaved caspase-1, IL-18 and Caspase-5.
