# Supplementary material for: Metformin induces pyroptosis in leptin receptor-defective hepatocytes via overactivation of the AMPK axis
Source: Cell Death Dis. 2023 Feb 3;14(2):82. doi: 10.1038/s41419-023-05623-4 (PMC9898507; doi:10.1038/s41419-023-05623-4)

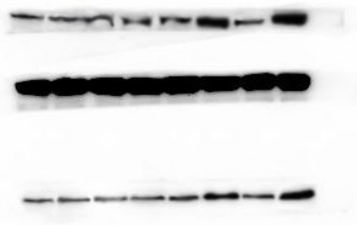

Supplement: Supplementary file 4 — Fig 4- Single Original Western blotting images [file 01 CCO & beta-actin & Cleaved cas3.pdf › Fig 4- Single Original Western blotting images -/Figure 4A/01 CCO & beta-actin & Cleaved cas3.pdf]
